# Benchmarking of protein descriptor sets in proteochemometric modeling (part 1): comparative study of 13 amino acid descriptor sets

**DOI:** 10.1186/1758-2946-5-41

**Published:** 2013-09-23

**Authors:** Gerard JP van Westen, Remco F Swier, Jörg K Wegner, Adriaan P IJzerman, Herman WT van Vlijmen, Andreas Bender

**Affiliations:** 1 Division of Medicinal Chemistry, Leiden / Amsterdam Center for Drug Research, Einsteinweg 55, Leiden 2333, CC, The Netherlands; 2 Tibotec BVBA, Turnhoutseweg 30, Beerse 2340, Belgium; 3 Unilever Centre for Molecular Science Informatics, Department of Chemistry, University of Cambridge, Lensfield Road, Cambridge CB2 1EW, United Kingdom; 4 Current affiliations: ChEMBL Group, European Molecular Biology Laboratory European Bioinformatics Institute (EMBL-EBI), Wellcome Trust Genome Campus, CB10 1SD Hinxton, United Kingdom

**Keywords:** GPCR, HIV, QSAM, Peptides, Amino acid index, Protein descriptor, Polypharmacology

## Abstract

**Background:**

While a large body of work exists on comparing and benchmarking of descriptors of molecular structures, a similar comparison of protein descriptor sets is lacking. Hence, in the current work a total of 13 different protein descriptor sets have been compared with respect to their behavior in perceiving similarities between amino acids. The descriptor sets included in the study are Z-scales (3 variants), VHSE, T-scales, ST-scales, MS-WHIM, FASGAI and BLOSUM, and a novel protein descriptor set termed ProtFP (4 variants). We investigate to which extent descriptor sets show collinear as well as orthogonal behavior via principal component analysis (PCA).

**Results:**

In describing amino acid similarities, MSWHIM, T-scales and ST-scales show related behavior, as do the VHSE, FASGAI, and ProtFP (PCA3) descriptor sets. Conversely, the ProtFP (PCA5), ProtFP (PCA8), Z-Scales (Binned), and BLOSUM descriptor sets show behavior that is distinct from one another as well as both of the clusters above. Generally, the use of more principal components (>3 per amino acid, per descriptor) leads to a significant differences in the way amino acids are described, despite that the later principal components capture less variation per component of the original input data.

**Conclusion:**

In this work a comparison is provided of how similar (and differently) currently available amino acids descriptor sets behave when converting structure to property space. The results obtained enable molecular modelers to select suitable amino acid descriptor sets for structure-activity analyses, *e.g.* those showing complementary behavior.

## Background

### Proteochemometric modeling

Proteochemometric (PCM) modeling uses statistical modeling techniques to model ligand–target interaction space [1-6]. Related to Quantitative Structure-Activity Relationship (QSAR) modeling but expanding on the concept, PCM modeling takes *both* ligand- and target space into account. Hence PCM techniques enable the models to extrapolate - within limits imposed by the data sets, descriptors, and modeling method - in both the chemical domain (to related ligands), and the biological domain (to related targets). Applications include receptor deorphanization, [7]–[10], virtual screening for compounds with a desired activity profile across members of a receptor / transporter family (e.g. the adenosine receptor family) [9,11], and the combined modeling of orthosteric and allosteric compounds (e.g. nucleoside and non-nucleoside HIV reverse transcriptase inhibitors) [6]. Given that ligand and target descriptors jointly form a PCM model, the target description is as important as the ligand description. Several publications are available using varying ligand descriptors [7,12,13], yet on the side of target description there is less literature available. Moreover, most previous PCM modeling work uses the same descriptor set, the Z-scales published by Sandberg et al.[14], obtained from the field of Quantitative Sequence-Activity Modeling (QSAM) [1,14-18]. Limited literature is available using different approaches for target description but these are in most cases physicochemical properties similar to the z-scales, [5,7,19]. Alternatively there are methods not relying on the target sequence (as is the case with QSAM descriptor sets) but also on structural features of the binding site [5,20-24]. However, the major strength of PCM is that no structural information is needed, yet a systematic investigation of suited protein descriptors is lacking in the literature.

### Utilization of Quantitative Sequence Activity Modeling (QSAM) derived descriptor sets

QSAM attempts to quantitatively model the binding affinity of small peptide drugs to macromolecular targets, similar to QSAR in the field of small molecules. In this context several descriptor sets for amino acids (AAs) have been developed [25]. The majority of these descriptor sets rely on a principal component analysis (PCA) of a large property matrix used to describe the individual AAs, reducing dimensionality while still describing typically over 80% of the variation present in the original set [14]. This leads to descriptor sets that can correlate peptide make-up with an output variable as long as this output variable can be described in terms of individual AA properties.

The QSAM derived Z-scales descriptor set, arguably the most widely used descriptor set in PCM modeling, was intended to be used in research for small peptide drugs. Hence, the set covers also non-natural AAs (which can also be said about the T-scales and ST-scales descriptors introduced later). Therefore, if the original matrix consists of over 167 AAs (ST-scales) of which only 20 are natural AAs (which are relevant in bioactivity modeling), then the principle components (PCs) derived from the PCA might not be the ones capturing most of the information that matters in our case. Hence this leads to potentially less resolution in the space we are particularly interested in generating accurate PCM models, namely the space formed by the natural amino acids [26].

In order to capture the current state-of-the-art in describing AA (and peptide) properties, and to potentially improve upon the current situation, in this work we have compared 9 previously published and four novel AA descriptor sets (referred to as ProtFP in the text) in order to evaluate how they describe AA (dis)similarities (see Methods for a detailed explanation).

### Amino acid descriptor sets considered in this study

In the current work individual descriptor sets are considered that can be subdivided in a number of broad classes (Table 1). Firstly, three descriptor sets, namely Z-scales ((using 3 PCs, 5 PCs, or binned denoted by (3), (5) or (Binned)) [14], VHSE [27], and ProtFP PCA (using 3, 5 or 8 PCs), are based on a PCA analysis of physicochemical properties. Secondly, ST-scales and T-scales consist of a PCA of mostly topological properties [26,28]. FASGAI, part of the third category of descriptor sets tested is based on a factor analysis of physicochemical properties [29]. Furthermore, we also tested two descriptor sets that are calculated in a very different manner compared to the first six, namely a descriptor set based on three dimensional electrostatic properties calculated per AA (MS-WHIM) [30]. Additionally, a descriptor set based on a VARIMAX analysis of physicochemical properties which were subsequently converted to indices based on the BLOSUM62 substitution matrix (BLOSUM) [31]. Finally, we tested a descriptor set only describing each AA by a single feature (ProtFP (Feature)), which is expected to display very dissimilar behavior from the others [11,32]. See Table 1 for a complete overview.

**Table 1:** Amino acid descriptor sets analyzed in the current study

**Descriptor set**	**Type**	**Derived by**	**# of components**	**Variance explained**	**AAs covered**
BLOSUM	Physicochemical and substitution matrix	VARIMAX	10	n/a	20
FASGAI	Physicochemical	Factor Analysis	6	84%	20
MSWHIM	3D electrostatic potential	PCA	3	61%	20
ProtFP (PCA3)	Physicochemical	PCA	3	75%	20
ProtFP (PCA5)	Physicochemical	PCA	5	83%	20
ProtFP (PCA8)	Physicochemical	PCA	8	92%	20
ProtFP (Feature)	Feature based	Hashing	n/a	n/a	20
ST-scales	Topological	PCA	5	91%	167
T-scales	Topological	PCA	8	72%	135
VHSE	Physicochemical	PCA	8	77%	20
Z-scales (3)	Physicochemical	PCA	3	n/a	87
Z-scales (5)	Physicochemical	PCA	5	87%	87
Z-scales (Binned)	Physicochemical	PCA followed by binning	n/a	n/a	20

The first column contains the name of the descriptor set as used in the main text. Further listed are the type, dimensionality reduction, number of components and variance of the original matrix explained. The last column differentiates between descriptor sets only covering the natural amino acids or more. Not available is abbreviated by n/a.

While characterizing how similar descriptor sets perceive amino acid space is one important requirement to select the best descriptor set to use in PCM, another important quantification comes from benchmarking on PCM sets. These benchmarks are performed in the companion paper [33].

## Results and discussion

### PCA of final indices selection (for ProtFP (PCA))

**Figure 1:**
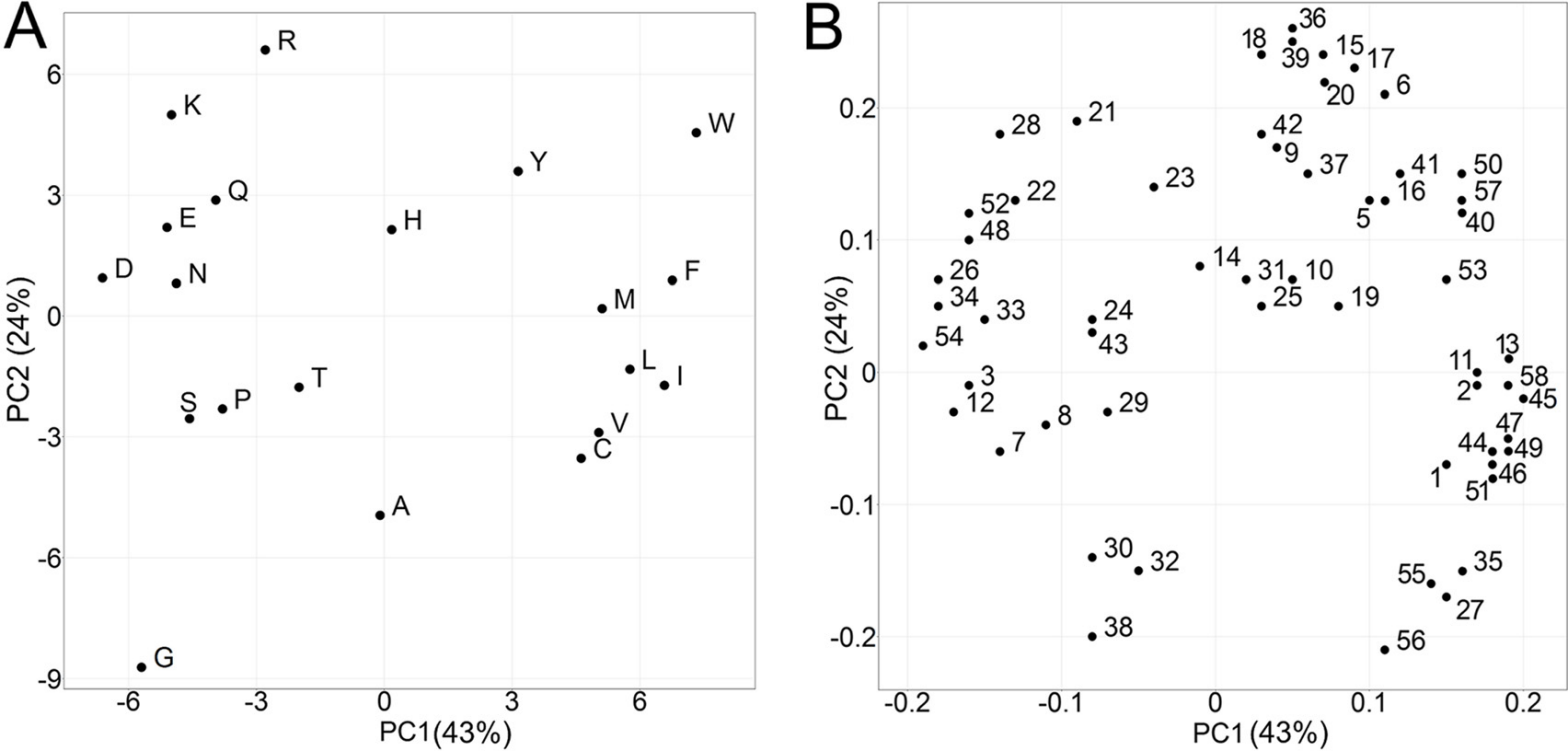
**Principal components resulting from the PCA on 58 AAindices making the ProtFP descriptor set. (A)** AAs that share physicochemical properties cluster together (see text for discussion of details). The amount of variance explained by each principal component is shown in brackets. **(B)** The corresponding loadings plot where the numbers correspond to Additional file 1: Table S1.

Before comparing the different descriptor the novel descriptor set to was analyzed get an idea of the descriptors abilities to characterize differences and similarities between the natural amino acids via PCA. Figure 1A shows the first two principle components of all 20 natural AAs when employing the ProtFP descriptor set. Overall, the plot shows a general clustering of AAs with similar properties with the first PC corresponding to hydrophobicity (F and I score high whereas D and E score low) and the second PC corresponding to size (W and K score high whereas G and A score low). While the type of information generally captured by the first components is also seen for other descriptor sets, noteworthy here is the clustering of Leucine and Isoleucine, which is intuitively correct due to their high chemical similarity. However this L – I clustering is not reproduced by all AA descriptor sets, like ST-scales (Additional file 1: Figure S1). Furthermore, both charged (D, E and R, K) and aromatic residues (F, H, Y, W) form sub-clusters as well. (The principle components, representing each AA in ProtFP space, can be found in Table 2.) Hence, overall the ProtFP descriptor set produces a clustering pattern that looks correct from a *chemical* point of view (which is relevant in structure-activity modeling like PCM).

**Table 2:** Principal components resulting from the AAindex selection

**Amino acid**	**PC1**	**PC2**	**PC3**	**PC4**	**PC5**	**PC6**	**PC7**	**PC8**	**Feature**
Variance explained	0.43	0.24	0.08	0.06	0.04	0.03	0.03	0.02	n/a
Total variance explained	0.43	0.67	0.75	0.81	0.85	0.88	0.90	0.92	n/a
G	−5.70	−8.72	4.18	−1.35	−0.31	2.91	0.32	−0.11	−176196525
A	−0.10	−4.94	−2.13	1.70	−0.39	1.06	−1.39	0.97	1169372512
C	4.62	−3.54	1.50	−1.26	3.27	−0.34	−0.47	−0.23	892384356
V	5.04	−2.90	−2.29	1.38	0.06	0.08	1.79	−0.38	−58134849
L	5.76	−1.33	−1.71	0.63	−1.70	0.71	−0.05	−0.51	−590269326
I	6.58	−1.73	−2.49	1.09	−0.34	−0.28	1.97	−0.92	−1784790725
M	5.11	0.19	−1.02	0.15	0.13	−0.30	−2.95	0.50	−188476976
F	6.76	0.88	0.89	−1.12	−0.49	−0.55	−0.87	1.05	−1561345091
W	7.33	4.55	2.77	−2.41	−1.08	1.04	0.23	0.59	−816166777
Y	3.14	3.59	2.45	−1.27	−0.06	−0.29	1.99	0.30	1237879003
H	0.17	2.14	1.20	0.71	1.16	−0.38	−1.85	−2.79	−1970548995
T	−2.00	−1.77	−0.70	1.02	1.06	−1.20	0.74	1.65	−266397547
P	−3.82	−2.31	3.45	1.00	−3.22	−3.54	−0.36	−0.30	−576206913
S	−4.57	−2.55	−0.67	1.11	0.99	−1.02	0.11	0.65	−1481898440
D	−6.61	0.94	−3.04	−4.58	0.48	−1.31	0.10	0.94	1957532765
N	−4.88	0.81	0.14	−0.14	1.23	−0.65	1.02	−1.94	−1593568836
E	−5.10	2.20	−3.59	−2.26	−2.14	1.35	−0.45	−1.31	558044215
Q	−3.95	2.88	−0.83	0.52	0.90	0.55	−0.08	0.64	−1986194934
K	−4.99	5.00	0.70	3.00	−1.23	1.41	0.19	0.87	268201585
R	−2.79	6.60	1.21	2.07	1.67	0.76	0.00	0.32	1636879004

Shown are all eight principal components and the variance explained by these principal components. In addition, the features obtained from the hashing of the AAindex selection are shown. This column represents the feature based ProtFP. Not available is abbreviated by n/a.

Figure 1B shows the loadings plot of the first 2 PCs that represent the ProtFP descriptor set. (For a complete list of indices used as input for the PCA please see Additional file 1: Table S1.) Here, some interesting observations can be made. For instance, reference 24 and 43 correspond to AAindex FAUJ880112 and MONM990201, respectively. While the former is a measure for negative charge, the latter is a measure for ‘averaged turn propensities in a transmembrane helix’. These two properties are close neighbors based on the first two components; however they have a relatively large distance in the third PC. This is interpretable in the following way: it is likely that charged residues, if present in a transmembrane region, initiates a turn and is therefore located at the edges of the TM region. Hence the clustering of these indices together can be rationally explained. References 36 and 39 are another interesting case. The former corresponds to AAindex LEVM760102 (Distance between C-alpha and centroid of side chain) and the latter corresponds to LEVM760105 (Radius of gyration of side chain). It is interesting to see that these two indices end up so close together in the first, second and third principal component. However, this is indeed expected as the maximal range of gyration can only be large if the maximal distance possible between C-alpha and side chain center is large and vice versa.

In conclusion, the division of the AA over the principal component space seems interpretable and in agreement with biochemical intuition; this applies both to the scores and the loadings plot of the PCA we performed. The next step is to compare the new descriptor set ProtFP to existing descriptor sets that have previously been published, both with respect to their ability to capture similarities of AAs and their relative performance in incorporating protein information relevant to bioactivity into SAR models.

### Comparison of descriptor set similarity matrices

The aim of the current study was to compare the behavior of AA descriptor sets, in order to investigate which descriptor sets agree on grouping AAs as similar, and which ones show largely orthogonal behavior. This provides a reference to select diverse descriptor sets when sampling several in a PCM project rather than needing to benchmark all of them.

**Figure 2:**
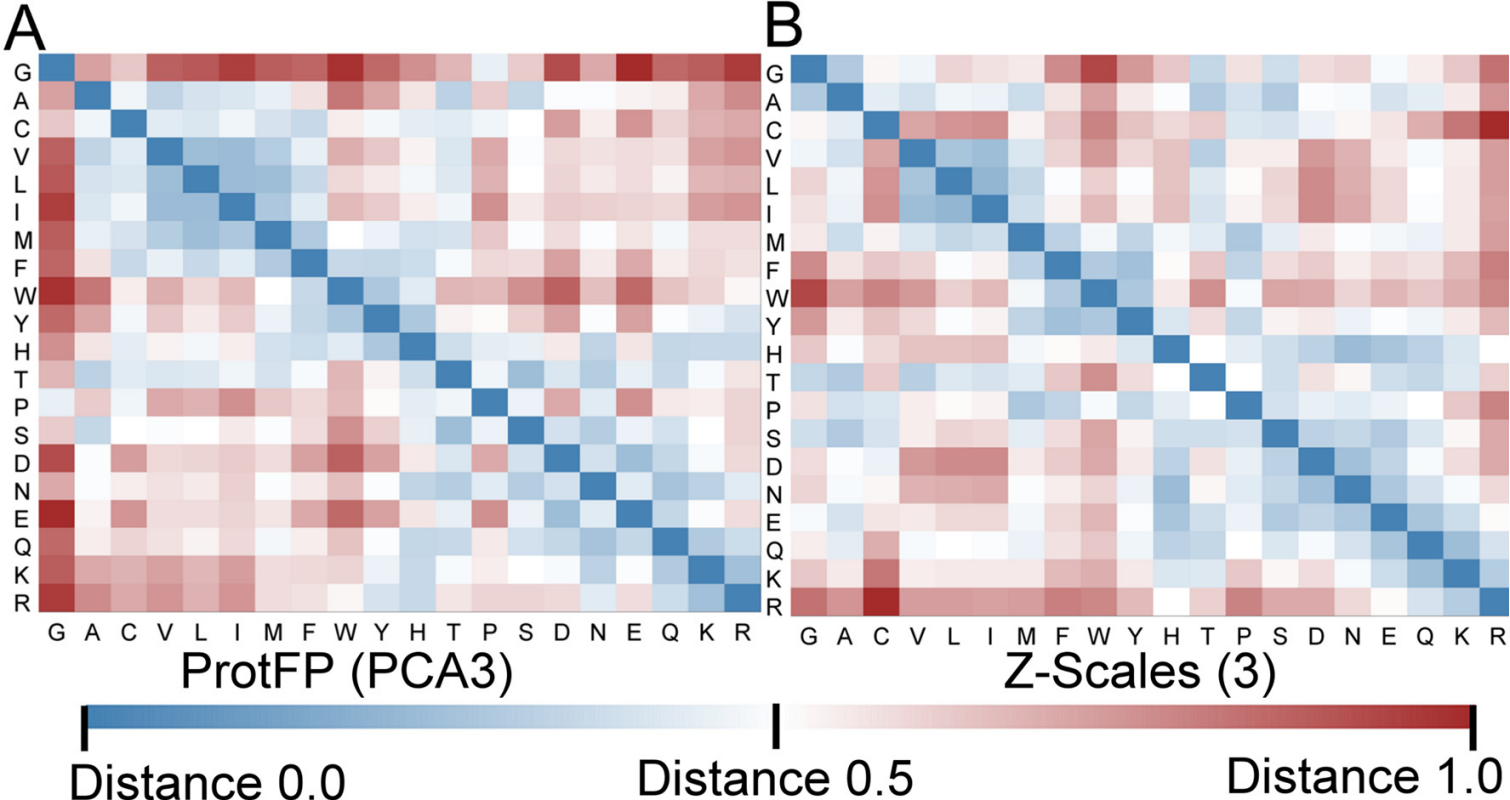
**Comparison of the distances between individual AA pairs. (A)** Amino acid similarity matrix derived from ProtFP (PCA3) descriptor set. **(B)** Corresponding analysis for Z-scales. In particular Histidine and Cysteine show a different distance spectrum when their similarity to the other AAs is compared (see text for detailed discussion).

To visualize similarities in behavior a Euclidian distance based similarity matrix of all 20 by 20 AAs was calculated and visualized in a heat map for each pair of numerical descriptor sets (12 sets). The comparison of ProtFP (PCA3) (using 3 principal components per amino acid as descriptor) with the frequently employed Z-scales (3) (again using 3 principal components or z-scales) is shown in Figure 2. (The analogous plots, as well as numerical descriptions of the similarity matrices of other AA descriptor sets, are provided in Additional file 1: Tables S2 to S13, as well as Additional file 1: Figures S2 to S13 for utilization by the reader in potential future studies).

Several clear differences are noteworthy when comparing the two descriptor sets. Firstly, the mean distances in the ProtFP (PCA3) heat map are larger compared to Z-scales (3), despite the fact that scaling that was applied (see Methods for details). This indicates that the ProtFP (PCA3) is more scattered through the PCA space than the Z-Scales (3).

Also for individual amino acids differences are apparent. Glycine is located further away from the rest of the amino acids in ProtFP (PCA3), compared to Z-scales (3). Conversely, Phenylalanine, Tryptophan, and Cysteine are located closer to the aliphatic and aromatic AAs, but further away from the charged residues. Finally, Histidine also displays a different profile as it has a central position between the charged residues and aromatic residues in ProtFP (PCA3), whereas it is closely located to the charged AAs in Z-scales. Interestingly both Cysteine and Histidine are residues that can exist in forms with slightly different physicochemical properties. Cysteine can be present as an individual amino acid or it can be part of a disulfide bridge (which leads to a shift in physicochemical properties compared to the unbridged form). Histidine on the other hand can be both a neutral and a protonated (positively charged) amino acid at physiological pH depending on the local amino acid environment. It stands to reason that it is no coincidence that these two amino acids display the largest differences.

In conclusion, both descriptor sets interpret the physicochemical space differently, and while both views can be rationalized, benchmark experiments are needed to determine which leads to more predictive models (which is what is provided in a follow-up study) [33].

### Differences between descriptor sets (PCA on all descriptor set principal components)

**Figure 3:**
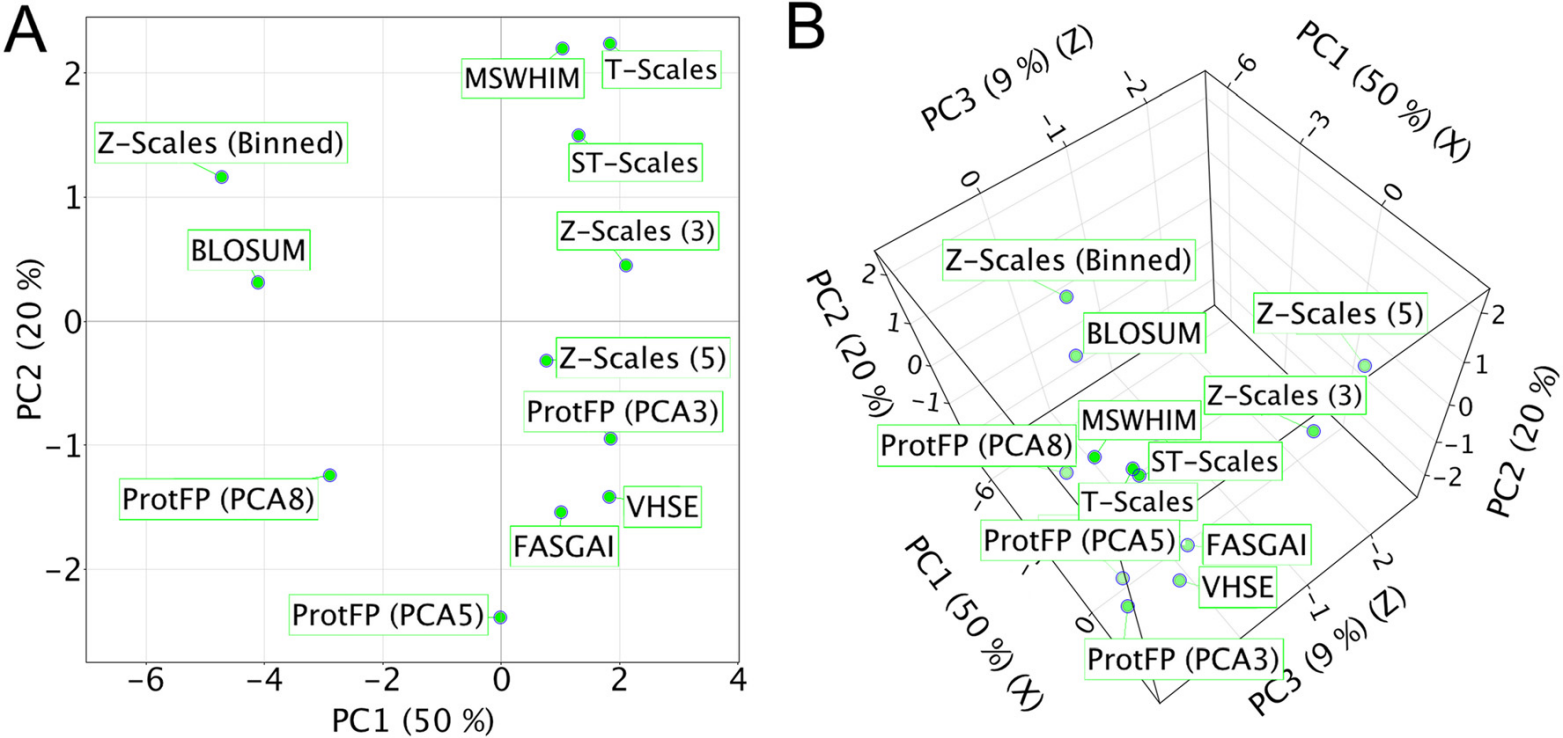
**PCA of the distances between the different descriptor sets.** Shown are the first two components **(A)**. T-scales, ST-scales and MSWHIM, and to a lesser extent Z-Scales (3) cluster together, as do FASGAI, VHSE, ProtFP (PCA3), and Z-Scales (5). Furthermore Z-Scales (Binned) and BLOSUM are nearest neigbors. ProtFP (PCA5) and (8) are seen to cluster away from the others. Furthermore. **(B)** When the first three PCs are displayed Z-scales (5) and ProtFP (PCA3) are seen to be distant from their cluster in the first two PCs.

In order to understand similarities between amino acid descriptors on a large scale, we performed a PCA on the inter-descriptor matrix of all numerical amino acid descriptor sets considered in the current study (12 versus 12 descriptor sets, excluding the feature based ProtFP (Feature), an analysis that we will refer to as the *full PCA analysis*. This analysis can be said to form the heart of the current study, and can express how similar, *on average*, two descriptor sets perceive any pair of AAs to be, and to establish how correlated their similarity perceptions are. Figure 3A shows the results of the PCA of the average distance between all descriptor sets; shown are the 2 first PCs (explaining 70% of the variance).

The first thing noteworthy in Figure 3A is that MSWHIM, T-scales and ST-scales cluster together (here in the upper right quadrant); similarly, VHSE, FASGAI and ProtFP (PCA3) form a second cluster (here in the lower right quadrant). The space between these two clusters is occupied by Z-scales (3) (upper right) and Z-scales (5) (lower right). ProtFP (PCA5) and ProtFP (PCA8) occupy the lower left quadrant but do not cluster. Finally Z-Scales (Binned) and BLOSUM behave distinctly from all descriptor sets above, and occupy the upper left quadrant. The distance between Z-scales (5) and Z-scales (Binned) is very large, which was not expected as one is constructed from the other. It could be speculated that the division into bins maximized separation between amino acids that only differ slightly on a continuous scale explaining the very different behavior. Figure 3B shows the results of the same PCA in three dimensions; now we observe that ProtFP (PCA3), Z-scales (3), and Z-scales (5) are in addition to dissimilarities in the first two dimensions also out of the plane of the other descriptor sets.

### Differences between descriptor sets (PCA on first two descriptor set principal components)

**Figure 4:**
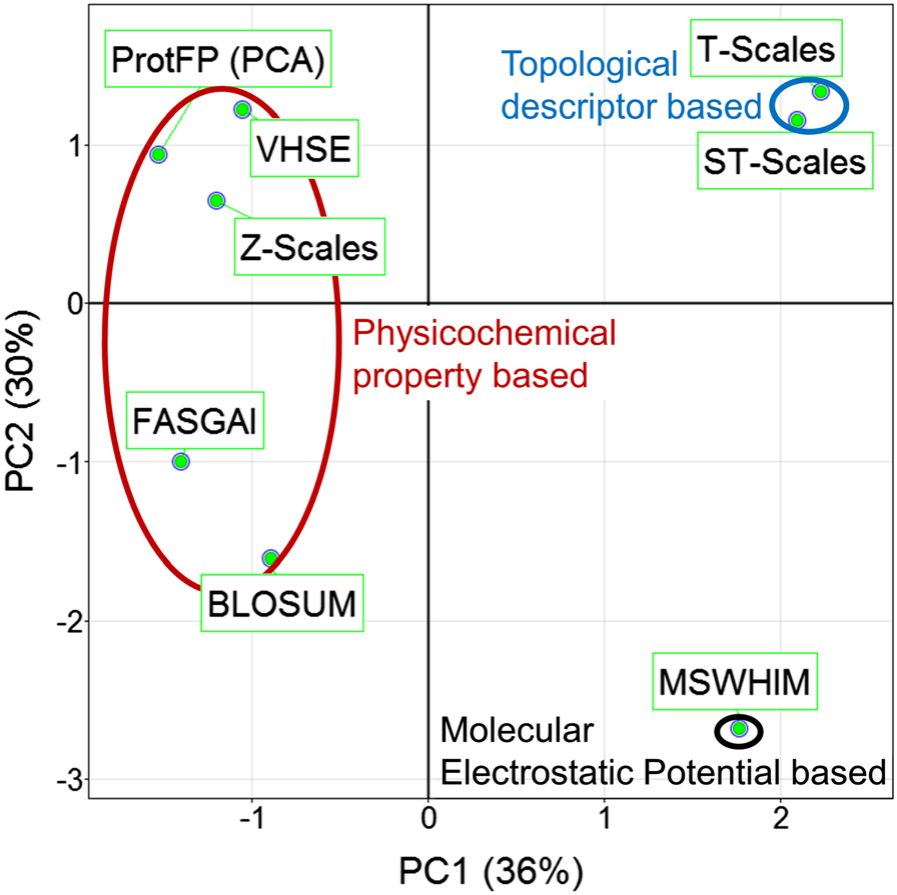
**PCA of the distances between the different descriptor sets using only the first two components per descriptor set.** Shown are the first two components that explain 66% of the variance. The descriptor sets are seen to cluster based on the way they are derived. In the top left physicochemical property based descriptor sets (all derived via PCA) are shown, in the bottom left the physicochemical property based descriptor sets that are derived differently are shown. The top right shows the descriptor sets derived from topological descriptors and the bottom right shows the only molecular electrostatic potential based descriptor.

The same calculation was repeated using only the absolute distance based on the *first two* PCs per descriptor set (in other words changing Z-Scales (3) to Z-Scales (2) and so forth), we will refer to this calculation as *limited PCA analysis*. The goal was to compare the descriptor sets based on the first two dimensions and thereby minimizing the differences generated by the inclusion of more dimensions (Additional file 1: Tables S14 – S21 and Figures S1, and S14 to S20). It was expected that this method would provide a more fundamental insight in descriptor set similarity. Since we only use the first PCs, the different versions of ProtFP (PCA) are identical as are the versions of Z-scales. Again shown are the first two PCs (Figure 4, which explain 66% of the variance). Surprisingly, all descriptor sets based on physicochemical properties are grouped and score negative on the first principal component. Moreover the descriptor sets form two clusters, one for the PCA derived descriptor sets (ProtFP (PCA), VHSE, and Z-scales), and one for the descriptors derived differently (FASGAI and BLOSUM). While it might seem surprising that the BLOSUM descriptor set and the FASGAI descriptor set are nearest neighbors in the first two principal components, there is a large distance between them in the 3rd principal component, accounting for the differences between them. Likewise, the two descriptors based on a topological description also cluster (T-scales and ST-scales). Finally, the MS-WHIM descriptor behaves most dissimilar to the others, likely due to the fact that this was the only descriptor constructed on an electrostatic potential.

The results indicate that the different descriptor sets indeed describe the AA space differently, although there are commonalities most often based on the way they are constructed. What can be observed overall is that the use of *more* principal components (>3 per AA for a particular descriptor set) leads to a significant shift in the way they describe the AA differences despite being generated from the same underlying matrix. This is true even while these principal components typically capture less variance of the original underlying matrix from which they were derived. One possible explanation is that the use of more than 3 principal components per AA might introduce less signal than noise, which is agreement with the small amount of variance captured by higher-order components. The *full PCA analysis* displays which descriptor sets cluster based on the first 2 principal components, hence this visualization could be used as a guideline to determine complementarity when selecting descriptors to be used in bioactivity modeling (*e.g.* by selecting one from each quadrant, which hence contain largely independent information). Another conclusion from the observations is that the descriptor sets introduced here (ProtFP (PCA3) – ProtFP (PCA8)) add novelty, as they characterize the AA space differently from the others.

As will be shown in a subsequent study, different AA descriptor sets capture different aspects of similarity and can all be used to construct bioactivity models. However given the target, different descriptor sets are found to perform well whereas other are found to perform sub par. The interested reader is referred to this companion study for details [33].

## Experimental

All experiments were performed on an Intel core i7 860 with 16 GB of memory, for further details please see Methods section below. Included in the supporting information (Additional file 2) is a pipeline pilot protocol that allows the transfer of single letter amino acids sequences into the here benchmarked descriptor sets.

## Conclusions

Given the large number of AA descriptor sets available there was a need for both a characterization of those descriptor sets with respect to their perception of similarities between AAs, and to benchmark them in bioactivity models. The former analysis has been presented here, and it was found that different clusters of amino acid descriptor sets emerge, as well as descriptors that behave differently from those clusters. As might be intuitive, when only considering the first two principal components, descriptor sets cluster the way they are derived, with Z-scales, VHSE and ProtFP (PCA) falling into one cluster, T-scales and ST-scales forming a second group of descriptor sets, and FASGAI, BLOSUM and MS-WHIM descriptor sets being somewhat distinct to the above groups.

Yet when considering the full descriptor sets, the clustering pattern shift significantly. This indicates that including more principal components changes descriptor behavior, while these principal components typically describe less variance than the first two components. The current work provides a guideline which descriptor can be considered complementary and should hence be sampled when creating novel PCM models. To determine implications for descriptor performance in bioactivity modeling the reader is referred to the companion study [33].

## Methods

A detailed outline of each descriptor set, illustrating the differences and similarities between all of them, is given below. For each descriptor set a short name used in the tables and figures is given in parentheses.

### Z-scales

Z-scales are based on physicochemical properties of the AAs including NMR data and thin-layer chromatography (TLC) data. Sandberg *et al.*[14] improved on the original Z-scales published by Hellberg *et al.*[15] by introducing two more Z-scales, bringing the total to five scales rather than three and using 26 properties derived for 87 AAs. The PCA mainly captures lipophilicity (Z1), bulk (Z2), polarity / charge (Z3). The fourth and fifth scale (Z4 and Z5) are more difficult to interpret relating to properties as electronegativity, heat of formation, electrophilicity and hardness. The total variance explained by these five components is 87%. In this study we employ the Z-scales using 5 scales (***Z-scales (5)***) and the Z-scales using 3 scales (***Z-scales (3)***), both of which have been used in previous work [9,10,34]. Furthermore, the 5 Z-scales were also binned into several classes per scale (***Z-scales (Binned)***). When an AA fell within one of these bins, the bin property was set ‘1’, otherwise it was set ‘0’. All natural amino acids were uniquely identifiable based on this classification. For instance Tryptophan is assigned a ‘1’for the following classes: Lipophilicity High, Size Large, Electronic Properties High, Electronegativity High and Electrophilicity Low, whereas Glycine is assigned a ‘1’ for the following: Lipophilicity Low, Size Small, Electronic Properties High, Electronegativity Medium Low and Electrophilicity Medium Low. The rationale was that these descriptors would be easier to interpret than descriptors derived from a PCA (see additional file 1: Table S22 for the classes) while at the same time also partially removing the ability of descriptors to interpolate between numerical property representations of amino acids.

### Vectors of hydrophobic, steric, and electronic properties

Originally published by Mei *et al.*, Vectors of Hydrophobic, Steric, and Electronic properties (***VHSE***) are obtained from 18 hydrophobic, 17 steric and 15 electronic properties, giving rise to a total of 50 physicochemical properties of the 20 natural AAs [27]. For each of these three categories a PCA was generated and resulted in Principal Components (PC) of two hydrophobic, two steric and four electronic properties with a total variance of 74.33%, 78.68% and 77.97%, respectively. These eight properties form the VHSE scales [27].

### T-scales

Published by Tian *et al*., the T-scale descriptor set (***T-scales***) is derived from several computer programs utilized to generate 67 common topological descriptors of 135 AAs [28]. These topological descriptors are based on the connectivity table of amino acids alone, and to not explicitly consider 3D properties of each structure. A PCA calculation of the five most representative descriptors was called the T scales. These five descriptors encompass 91.14% of the total variance of the data [28].

### ST-scales

Published by Yang *et al.*, the topological ST-scale (***ST-scales***) descriptor set extends the above T-scales by taking 827 properties into account which are mainly constitutional, topological, geometrical, hydrophobic, electronic, and steric properties of a total set of 167 AAs [35]. As opposed to T-scales, this descriptor set does employ 3D information about the amino acids; hence the molecular structures were first optimized, as some of the properties used are conformation-dependent. ST-scale utilizes eight PCs instead of the five PCs of T-scales and describes 71.5% of the total variance of the data [26].

### MS-WHIM

Previously published by Zaliani and Gancia, the MS-Whim (***MSWHIM***) descriptor set is derived from 36 electrostatic potential properties derived from the three-dimensional structure of the molecule [30]. These are calculated from 12 statistical parameters starting from x, y, z coordinates of the Connolly surface, which is a solvent-excluded surface (an inverse solvent-accessible surface) [36]. On these 36 parameters (3 coordinates by 12 parameters each) of the 20 natural AAs a PCA was performed which gave rise to a set of 3 principal components with a total variance of 61%, as well as a set of 7 principal components with a total of variance of 87%. However according to the loading plots, Zaliani and Gancia concluded that the most representative values were contained in the first three principal components and they hence chose to take only the first three principal components into account in their final descriptor set [30].

### Factor analysis scales of generalized amino acid information

Published by Guizhao and Zhiliang**,** the Factor Analysis Scales of Generalized AA Information (***FASGAI***) is derived from 335 physicochemical properties of the 20 natural AAs [29]. Contrary to the other descriptor sets a factor analysis is applied rather than a PCA. Factor analysis also simplifies large matrices of data like PCA does, however factor analysis computes a smaller number of factors that describe the *correlated* variables, whereas PCA searches for the parameters with the largest *variance*. After generating these factors, a PCA was applied to get the factors that would describe the data with the most variance. The PCA resulted in the FASGAI protein descriptor set of 6 principal components with a total variance of 83.5% [29].

### BLOSUM

Published by Georgiev, the BLOSUM matrix-derived amino acid descriptor set (***BLOSUM***) is the only AA descriptor set we employed that is not directly based on physical or chemical properties of the AAs, but on both physicochemical properties that have been subjected to a VARIMAX analyses and an alignment matrix of the 20 natural AAs, the BLOSUM62 matrix (for details see the work by Georgiev) [31,37]. This procedure renders scales analogous to the Z-scales. This descriptor set was added due to its fundamentally different nature and an anticipated complementarity in capturing AA properties, compared to other descriptor sets.

### Protein fingerprint (ProtFP)

In addition to the previously published descriptor sets, we also employed a novel AA descriptor set in this work which we termed ‘Protein Fingerprint’ (‘ProtFP’). ProtFP is based on a selection of different AA properties obtained from the AAindex database [38]. However, the difference to descriptor sets mentioned previously is that the descriptor was obtained using recursive elimination of the most co-varying properties after starting with the full set of indices.. The final descriptor set comes in several flavors. The first ProtFP descriptor (described in more detail below) is based on a PCA of the remaining indices employing 3, 5 or 8 principal components (***ProtFP (PCA3)***, ***ProtFP (PCA5)*** or ***ProtFP (PCA8)***), which allows for quantitative comparison of AAs. The second variation is based on a hashing approach of all indices values per AA (***ProtFP (Feature)***) resulting in a single feature per AA which we previously published on [11,32]. Given the novelty of the ProtFP descriptor sets, their derivation is described in more detail in the following.

### Selection of AAindices (for ProtFP)

The ProtFP descriptor set was constructed from a large initial selection of indices obtained from the AAindex database for all 20 naturally occurring AAs. This is a principal difference to several other AA descriptor sets, where also non-natural AAs were taken into account [38]. Covariance between indices was determined *via* PCA while indices were linearly scaled to a range between 0 and 1 rather than using the raw indices. The analysis was performed using the Pipeline Pilot implementation, version 6.1.5, of R-statistics and the ‘prcomp’ package, with the options of ‘mean centering’ and ‘scaling’ enabled [39,40]. Indices showing highest covariance were removed, while at the same time a number of largely independent physicochemical parameters were maintained. The final reduced selection consisted of 58 AAindices, which are hence (a) based on the relevant natural amino acids only, (b) largely independent (since those indices with larges covariance were removed). The final amino acid indices employed in the construction of the ProtFP descriptor set are listed in Additional file 1: Table S1.

### PCA of final indices selection (for ProtFP (PCA))

In order to obtain descriptors at lower dimensionality PCA was performed on the final set of 58 amino acid properties. The analysis was performed using default parameters, requiring a minimum explained variance of 75%, but forcing a minimum of 8 principal components (PCs) to be able to compare the descriptor sets head to head with all others. The first three PCs explained 75% of the variance, 5 PCs explained 83%, and 8 PCs explained 92%. In subsequent experiments three versions were used: the first three PCs (***ProtFP (PCA3)***), the first 5 PCs (***ProtFP (PCA5)***) or all eight PCs (***ProtFP (PCA8)***). See Table 2 for the final principal components.

### Distance between descriptor sets

**Figure 5:**
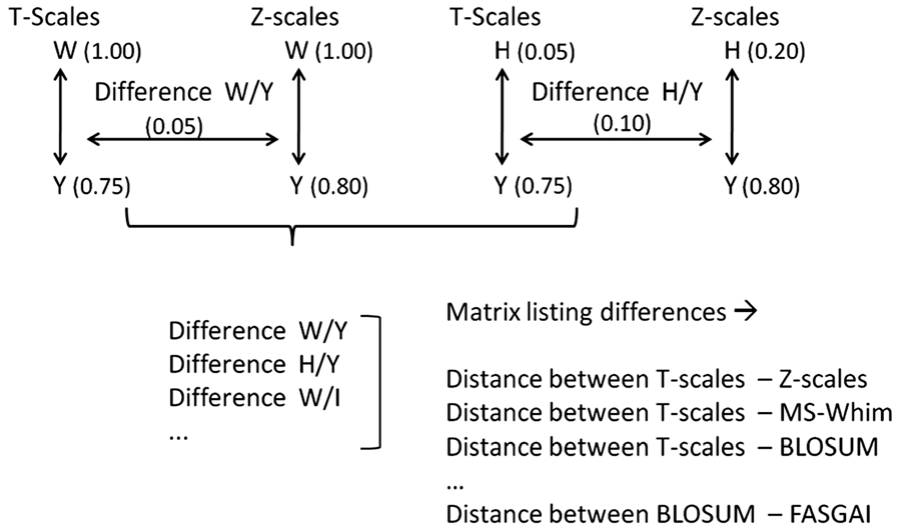
**Approach used to characterize the similarity of amino acid descriptor sets.** After normalization of all descriptor sets, the difference between a pair of descriptor sets was calculated. This difference was obtained as the difference between the distance separating a pair of AAs when represented by descriptor set 1, and the distance of the same pair when represented by descriptor set 2. This was done for all descriptor set pairs. Finally, the average difference was obtained and a full matrix was constructed, hence giving a measure of how similar different amino acid descriptors perceive amino acid structures to be.

To compare the characteristics of different descriptor sets and their behavior in describing particular AAs as similar and dissimilar, the average ‘difference in distances’ was calculated for each possible pair of descriptor sets (see Figure 5 for a scheme of the performed calculations). This value was obtained as follows. Firstly, a full similarity matrix was calculated for each possible AA pair using each descriptor set, thus consisting of 20*20 fields per descriptor set. The distances in this matrix were scaled linearly to a range between 0 (most similar) and 1 (most dissimilar). Subsequently, for each possible pair of *descriptor sets* the *difference* between the *Euclidian distances of each AA pair* was calculated, giving rise to a total of 400 inter-amino acid distance differences per descriptor set pair. (In other words, we evaluated how differently two descriptor sets judged the difference between two AAs. Given that 20 AAs exist, 400 distances exist between all AAs, for a single descriptor set – and the same number of *differences* of those distances for each descriptor set pair.)

Of the 400 distances obtained, the average distance and the standard deviation was calculated and subsequently employed as a measure for the distance between amino acid descriptor sets (*i.e.*, if the average distance is high, two amino acid descriptor sets perceive similarities between amino acids in a very different way). The more different those distances are for different descriptor sets, the more different the particular descriptor sets considered behave. We employed a total of 12 descriptor sets for this amino acid descriptor set comparison, since the feature based ProtFP descriptor set (***ProtFP (Feature)***) merely uses presence or absence of features and hence could not be included in the numerical distance calculation as all distances would be 1 (maximal). In the end, a inter descriptor set distance matrix of 12*12 distances between descriptor sets was obtained which was subject to PCA with the aim to visualize the individual distances between descriptor sets in a graphical way. (Conceptually, this work is similar to an analysis of chemical descriptors from the ligand side which was performed previously and given the importance of also comparing descriptors from the protein side the current work hence complements this study [41]).

## Abbreviations

AA: Amino acid; ACE: Angiotensin-converting enzyme; CV: Cross validated; FASGAI: Factor analysis scales of generalized AA information; PC: Principal component; PCA: Principal component analysis; PCM: Proteochemometric; ProtFP: Protein fingerprint; QSAM: Quantitative sequence-activity modeling; QSAR: Quantitative structure-activity relationship; Sens: Sensitivity; TLC: Thin layer chromatography; VHSE: Vectors of hydrophobic, steric, and electronic properties.

## Competing interests

The authors declare that they have no competing interests.

## Authors’ contributions

GJPvW conceived of the study, participated in its design, carried out calculations, and drafted the manuscript. RFS carried out calculations, and helped draft the manuscript. JKW participated in study design. APIJ helped draft the manuscript. HWTvV participated in study design and drafted the manuscript. AB drafted the manuscript and participated in study design. All authors read and approved the final manuscript.

## Supplementary Material

Additional file 1 PDF document containing 22 additional tables and 20 additional figures that support the main text. Click here for file

Additional file 2 Archive file containing a pipeline pilot component to transfer single letter amino acids sequences into the here benchmarked descriptor sets and an example protocol. Click here for file
