# Supplementary material for: Benchmarking of protein descriptor sets in proteochemometric modeling (part 1): comparative study of 13 amino acid descriptor sets
Source: J Cheminform. 2013 Sep 23;5:41. doi: 10.1186/1758-2946-5-41 (PMC3848949; doi:10.1186/1758-2946-5-41)
Supplement: Additional file 1 — PDF document containing 22 additional tables and 20 additional figures that support the main text. [file 1758-2946-5-41-S1.pdf]

Benchmarking of Protein Descriptor Sets in Proteochemometric Modeling (Part 1)

Comparative Study of 13 Amino Acid Descriptors

Additional File 1

Gerard J.P. van Westen [1], *Remco F. Swier* [1], *Jörg K. Wegner* [2], *Adriaan P. IJzerman* [1], *Herman W.T. van Vlijmen* [1,2], *Andreas Bender* [1,3]

[1] Division of Medicinal Chemistry,Leiden / Amsterdam Center for Drug Research, Einsteinweg 55, 2333 CC, Leiden, The Netherlands

[2] Tibotec BVBA, Turnhoutseweg 30, 2340 Beerse, Belgium

[3] Unilever Centre for Molecular Science Informatics, Department of Chemistry, University of Cambridge, Lensfield Road, Cambridge, CB2 1EW, United Kingdom

Table of Contents:

Supporting Table S1. List of Indices used along with a description of the index. 4
Supporting table S2. Scaled distance matrix ProtFP_PCA (3). 6
Supporting table S3. Scaled distance matrix ProtFP_PCA (5). 7
Supporting table S4. Scaled distance matrix ProtFP_PCA (8). 8
Supporting table S5. Scaled distance matrix Z-Scales (3). 9
Supporting table S6. Scaled distance matrix Z-Scales (5). 10
Supporting table S7. Scaled distance matrix Z-Scales (Binned). 11
Supporting table S8. Scaled distance matrix VHSE. 12
Supporting table S9. Scaled distance matrix ST-Scales. 13
Supporting table S10. Scaled distance matrix T-Scales. 14
Supporting table S11. Scaled distance matrix MSWHIM. 15
Supporting table S12. Scaled distance matrix FASGAI. 16
Supporting table S13. Scaled distance matrix BLOSUM. 17
Supporting table S14. Scaled distance matrix ProtFP_PCA using 2 PCs. 18
Supporting table S15. Scaled distance matrix Z-Scales using 2 PCs. 19
Supporting table S16. Scaled distance matrix VHSE using 2 PCs. 20
Supporting table S17. Scaled distance matrix ST-Scales using 2 PCs. 21
Supporting table S18. Scaled distance matrix T-Scales using 2 PCs. 22
Supporting table S19. Scaled distance matrix MSWHIM using 2 PCs. 23
Supporting table S20. Scaled distance matrix FASGAI using 2 PCs. 24
Supporting table S21. Scaled distance matrix BLOSUM using 2 PCs. 25
Supporting Table S22. The binned Z-Scales 26
Supporting figure S1. Plot of the first two PCs in the ST-scales descriptor set. 27
Supporting Figure S2. Heat map of the ProtFP_PCA (3) descriptor set 28
Supporting Figure S3. Heat map of the ProtFP_PCA (5) descriptor set 29
Supporting Figure S4. Heat map of the ProtFP_PCA (8) descriptor set 30
Supporting Figure S5. Heat map of the Z-Scales (3) descriptor set 31
Supporting Figure S6. Heat map of the Z-Scales (5) descriptor set 32
Supporting Figure S7. Heat map of the Z-Scales (Binned) descriptor set 33
Supporting figure S8. Heat map of the VHSE descriptor set 34
Supporting figure S9. Heat map of the ST-Scales descriptor set 35
Supporting figure S10. Heat map of the T-Scales descriptor set 36
Supporting figure S11. Heat map of the MSWHIM descriptor set 37

Supporting figure S12. Heat map of the FASGAI descriptor set 38
Supporting figure S13. Heat map of the BLOSUM descriptor set 39
Supporting figure S14. Plot of the first two PCs in the ProtFP_PCA descriptor set. 40
Supporting figure S15. Plot of the first two PCs in the Z-scales descriptor set............... 41
Supporting figure S16. Plot of the first two PCs in the VHSE descriptor set.................. 42
Supporting figure S17. Plot of the first two PCs in the T-scales descriptor set............... 43
Supporting figure S18. Plot of the first two PCs in the MSWHIM descriptor set........... 44
Supporting figure S19. Plot of the first two PCs in the FASGAI descriptor set.............. 45
Supporting figure S20. Plot of the first two PCs in the BLOSUM descriptor set. 46

Supporting Table S1. List of Indices used along with a description of the index.

Number	Identifier	Descriptor
1	ARGP820103	Membrane-buried preference parameters
2	BAEK050101	Linker index
3	BHAR880101	Average flexibility indices
4	CASG920101	Hydrophobicity scale from native protein structures
5	CHAM810101	Steric parameter
6	CHAM820101	Polarizability parameter
7	CHAM830101	The Chou-Fasman parameter of the coil conformation
8	CHAM830107	A parameter of charge transfer capability
9	CHAM830108	A parameter of charge transfer donor capability
10	CHOP780201	Normalized frequency of alpha-helix
11	CHOP780202	Normalized frequency of beta-sheet
12	CHOP780203	Normalized frequency of beta-turn
13	CIDH920105	Normalized average hydrophobicity scales
14	COSI940101	Electron-ion interaction potential values
15	FASG760101	Molecular weight
16	FAUJ880102	Smoothed upsilon steric parameter
17	FAUJ880103	Normalized van der Waals volume
18	FAUJ880104	STERIMOL length of the side chain
19	FAUJ880105	STERIMOL minimum width of the side chain
20	FAUJ880106	STERIMOL maximum width of the side chain
21	FAUJ880109	Number of hydrogen bond donors
22	FAUJ880110	Number of full nonbonding orbitals
23	FAUJ880111	Positive charge
24	FAUJ880112	Negative charge
25	FAUJ880113	pK-a(RCOOH)
26	GRAR740102	Polarity
27	JANJ780102	Percentage of buried residues
28	JANJ780103	Percentage of exposed residues
29	JOND920102	Relative mutability
30	JUNJ780101	Sequence frequency
31	KLEP840101	Net charge
32	KOEP990101	Alpha-helix propensity derived from designed sequences
33	KOEP990102	Beta-sheet propensity derived from designed sequences
34	KRIW790101	Side chain interaction parameter
35	KYTJ820101	Hydropathy index
36	LEVM760102	Distance between C-alpha and centroid of side chain
37	LEVM760103	Side chain angle theta(AAR)
38	LEVM760104	Side chain torsion angle phi(AAAR)
39	LEVM760105	Radius of gyration of side chain
40	LEVM760106	van der Waals parameter R0

41	LEVM760107	van der Waals parameter epsilon
42	MITS020101	Amphiphilicity index
43	MONM990201	Averaged turn propensities in a transmembrane helix
44	NISK800101	8 A contact number
45	NISK860101	14 A contact number
46	PONP800101	Surrounding hydrophobicity in folded form
47	PONP930101	Hydrophobicity scales
48	RACS770103	Side chain orientational preference
49	RADA880108	Mean polarity
50	ROSG850101	Mean area buried on transfer
51	ROSG850102	Mean fractional area loss
52	ROSM880102	Side chain hydropathy, corrected for solvation
53	TAKK010101	Side-chain contribution to protein stability
54	VINM940101	Normalized flexibility parameters (B-values), average
55	WARP780101	Average interactions per side chain atom
56	WOLR810101	Hydration potential
57	ZHOH040102	Relative stability scale extracted from mutation experiments
58	ZHOH040103	Buriability

Supporting table S2. Scaled distance matrix ProtFP_PCA (3).

Residue	G	A	C	V	L	I	M	F	W	Y	H	T	P	S	D	N	E	Q	K	R
G	0.00	0.72	0.63	0.87	0.89	0.95	0.88	0.86	0.98	0.85	0.76	0.67	0.44	0.62	0.92	0.70	1.00	0.85	0.88	0.96
A	0.72	0.00	0.46	0.33	0.39	0.40	0.44	0.57	0.82	0.71	0.56	0.32	0.62	0.34	0.49	0.49	0.53	0.54	0.70	0.77
C	0.63	0.46	0.00	0.42	0.40	0.46	0.39	0.35	0.54	0.47	0.43	0.42	0.46	0.50	0.73	0.54	0.75	0.60	0.68	0.70
V	0.87	0.33	0.42	0.00	0.24	0.23	0.30	0.44	0.69	0.63	0.53	0.40	0.70	0.49	0.59	0.57	0.58	0.57	0.72	0.75
L	0.89	0.39	0.40	0.24	0.00	0.23	0.24	0.35	0.59	0.55	0.47	0.41	0.68	0.50	0.60	0.55	0.58	0.53	0.67	0.68
I	0.95	0.40	0.46	0.23	0.23	0.00	0.28	0.42	0.66	0.62	0.54	0.46	0.76	0.55	0.62	0.61	0.60	0.59	0.73	0.75
M	0.88	0.44	0.39	0.30	0.24	0.28	0.00	0.29	0.50	0.45	0.38	0.39	0.63	0.49	0.58	0.49	0.56	0.47	0.58	0.58
F	0.86	0.57	0.35	0.44	0.35	0.42	0.29	0.00	0.35	0.34	0.36	0.48	0.59	0.58	0.72	0.55	0.70	0.54	0.59	0.57
W	0.98	0.82	0.54	0.69	0.59	0.66	0.50	0.35	0.00	0.28	0.42	0.67	0.66	0.76	0.87	0.65	0.85	0.64	0.60	0.52
Y	0.85	0.71	0.47	0.63	0.55	0.62	0.45	0.34	0.28	0.00	0.28	0.53	0.51	0.61	0.72	0.49	0.72	0.49	0.45	0.39
H	0.76	0.56	0.43	0.53	0.47	0.54	0.38	0.36	0.42	0.28	0.00	0.37	0.43	0.44	0.55	0.33	0.56	0.34	0.35	0.35
T	0.67	0.32	0.42	0.40	0.41	0.46	0.39	0.48	0.67	0.53	0.37	0.00	0.46	0.24	0.40	0.30	0.44	0.35	0.48	0.56
P	0.44	0.62	0.46	0.70	0.68	0.76	0.63	0.59	0.66	0.51	0.43	0.46	0.00	0.45	0.70	0.42	0.76	0.55	0.54	0.60
S	0.62	0.34	0.50	0.49	0.50	0.55	0.49	0.58	0.76	0.61	0.44	0.24	0.45	0.00	0.38	0.30	0.45	0.38	0.50	0.60
D	0.92	0.49	0.73	0.59	0.60	0.62	0.58	0.72	0.87	0.72	0.55	0.40	0.70	0.38	0.00	0.38	0.24	0.34	0.48	0.59
N	0.70	0.49	0.54	0.57	0.55	0.61	0.49	0.55	0.65	0.49	0.33	0.30	0.42	0.30	0.38	0.00	0.42	0.26	0.32	0.42
E	1.00	0.53	0.75	0.58	0.58	0.60	0.56	0.70	0.85	0.72	0.56	0.44	0.76	0.45	0.24	0.42	0.00	0.34	0.49	0.58
Q	0.85	0.54	0.60	0.57	0.53	0.59	0.47	0.54	0.64	0.49	0.34	0.35	0.55	0.38	0.34	0.26	0.34	0.00	0.29	0.36
K	0.88	0.70	0.68	0.72	0.67	0.73	0.58	0.59	0.60	0.45	0.35	0.48	0.54	0.50	0.48	0.32	0.49	0.29	0.00	0.25
R	0.96	0.77	0.70	0.75	0.68	0.75	0.58	0.57	0.52	0.39	0.35	0.56	0.60	0.60	0.59	0.42	0.58	0.36	0.25	0.00

Supporting table S3. Scaled distance matrix ProtFP_PCA (5).

Residue	G	A	C	V	L	I	M	F	W	Y	H	T	P	S	D	N	E	Q	K	R
G	0.00	0.73	0.74	0.84	0.85	0.90	0.81	0.78	0.90	0.77	0.75	0.68	0.61	0.65	0.91	0.68	0.94	0.81	0.93	0.98
A	0.73	0.00	0.72	0.30	0.41	0.37	0.44	0.60	0.87	0.72	0.56	0.36	0.68	0.37	0.82	0.53	0.68	0.53	0.66	0.75
C	0.74	0.72	0.00	0.64	0.78	0.68	0.56	0.59	0.76	0.61	0.53	0.54	0.99	0.60	0.84	0.57	1.00	0.66	0.97	0.76
V	0.84	0.30	0.64	0.00	0.33	0.22	0.30	0.48	0.76	0.64	0.51	0.39	0.77	0.46	0.84	0.56	0.72	0.53	0.70	0.72
L	0.85	0.41	0.78	0.33	0.00	0.28	0.33	0.40	0.63	0.58	0.57	0.52	0.65	0.58	0.83	0.63	0.61	0.59	0.66	0.78
I	0.90	0.37	0.68	0.22	0.28	0.00	0.28	0.45	0.71	0.62	0.53	0.45	0.79	0.53	0.84	0.60	0.69	0.56	0.70	0.74
M	0.81	0.44	0.56	0.30	0.33	0.28	0.00	0.31	0.55	0.43	0.38	0.38	0.73	0.47	0.73	0.47	0.64	0.44	0.63	0.60
F	0.78	0.60	0.59	0.48	0.40	0.45	0.31	0.00	0.35	0.31	0.44	0.53	0.68	0.61	0.76	0.55	0.69	0.55	0.70	0.68
W	0.90	0.87	0.76	0.76	0.63	0.71	0.55	0.35	0.00	0.31	0.59	0.76	0.75	0.83	0.85	0.71	0.78	0.71	0.80	0.76
Y	0.77	0.72	0.61	0.64	0.58	0.62	0.43	0.31	0.31	0.00	0.37	0.56	0.67	0.62	0.75	0.49	0.71	0.50	0.63	0.55
H	0.75	0.56	0.53	0.51	0.57	0.53	0.38	0.44	0.59	0.37	0.00	0.34	0.70	0.40	0.76	0.31	0.74	0.31	0.51	0.36
T	0.68	0.36	0.54	0.39	0.52	0.45	0.38	0.53	0.76	0.56	0.34	0.00	0.70	0.22	0.71	0.30	0.68	0.32	0.57	0.52
P	0.61	0.68	0.99	0.77	0.65	0.79	0.73	0.68	0.75	0.67	0.70	0.70	0.00	0.69	1.00	0.71	0.78	0.74	0.60	0.85
S	0.65	0.37	0.60	0.46	0.58	0.53	0.47	0.61	0.83	0.62	0.40	0.22	0.69	0.00	0.70	0.30	0.68	0.35	0.58	0.56
D	0.91	0.82	0.84	0.84	0.83	0.84	0.73	0.76	0.85	0.75	0.76	0.71	1.00	0.70	0.00	0.60	0.48	0.63	0.95	0.90
N	0.68	0.53	0.57	0.56	0.63	0.60	0.47	0.55	0.71	0.49	0.31	0.30	0.71	0.30	0.60	0.00	0.63	0.25	0.55	0.45
E	0.94	0.68	1.00	0.72	0.61	0.69	0.64	0.69	0.78	0.71	0.74	0.68	0.78	0.68	0.48	0.63	0.00	0.59	0.73	0.86
Q	0.81	0.53	0.66	0.53	0.59	0.56	0.44	0.55	0.71	0.50	0.31	0.32	0.74	0.35	0.63	0.25	0.59	0.00	0.47	0.38
K	0.93	0.66	0.97	0.70	0.66	0.70	0.63	0.70	0.80	0.63	0.51	0.57	0.60	0.58	0.95	0.55	0.73	0.47	0.00	0.46
R	0.98	0.75	0.76	0.72	0.78	0.74	0.60	0.68	0.76	0.55	0.36	0.52	0.85	0.56	0.90	0.45	0.86	0.38	0.46	0.00

Supporting table S4. Scaled distance matrix ProtFP_PCA (8).

Residue	G	A	C	V	L	I	M	F	W	Y	H	T	P	S	D	N	E	Q	K	R
G	0.00	0.75	0.79	0.86	0.82	0.94	0.96	0.86	0.84	0.84	0.95	0.86	1.00	0.78	0.99	0.82	0.89	0.80	0.87	0.92
A	0.75	0.00	0.70	0.58	0.48	0.67	0.49	0.58	0.81	0.82	0.80	0.54	0.88	0.48	0.82	0.78	0.72	0.52	0.63	0.71
C	0.79	0.70	0.00	0.66	0.71	0.71	0.62	0.57	0.72	0.65	0.65	0.60	0.97	0.56	0.78	0.61	0.93	0.62	0.92	0.70
V	0.86	0.58	0.66	0.00	0.40	0.22	0.74	0.62	0.74	0.58	0.79	0.52	0.89	0.52	0.83	0.58	0.75	0.57	0.71	0.70
L	0.82	0.48	0.71	0.40	0.00	0.40	0.55	0.48	0.59	0.62	0.69	0.64	0.80	0.59	0.82	0.65	0.57	0.56	0.63	0.71
I	0.94	0.67	0.71	0.22	0.40	0.00	0.77	0.65	0.74	0.58	0.78	0.61	0.89	0.60	0.86	0.58	0.74	0.64	0.76	0.75
M	0.96	0.49	0.62	0.74	0.55	0.77	0.00	0.41	0.69	0.80	0.64	0.66	0.87	0.61	0.80	0.80	0.76	0.57	0.75	0.69
F	0.86	0.58	0.57	0.62	0.48	0.65	0.41	0.00	0.41	0.51	0.74	0.54	0.76	0.57	0.70	0.73	0.76	0.53	0.69	0.65
W	0.84	0.81	0.72	0.74	0.59	0.74	0.69	0.41	0.00	0.41	0.83	0.76	0.91	0.79	0.82	0.79	0.77	0.64	0.71	0.68
Y	0.84	0.82	0.65	0.58	0.62	0.58	0.80	0.51	0.41	0.00	0.80	0.58	0.81	0.62	0.74	0.58	0.80	0.55	0.66	0.58
H	0.95	0.80	0.65	0.79	0.69	0.78	0.64	0.74	0.83	0.80	0.00	0.85	0.87	0.71	0.95	0.51	0.76	0.67	0.82	0.66
T	0.86	0.54	0.60	0.52	0.64	0.61	0.66	0.54	0.76	0.58	0.85	0.00	0.78	0.27	0.65	0.63	0.85	0.42	0.63	0.58
P	1.00	0.88	0.97	0.89	0.80	0.89	0.87	0.76	0.91	0.81	0.87	0.78	0.00	0.72	0.96	0.81	0.96	0.86	0.86	0.95
S	0.78	0.48	0.56	0.52	0.59	0.60	0.61	0.57	0.79	0.62	0.71	0.27	0.72	0.00	0.63	0.51	0.75	0.37	0.60	0.55
D	0.99	0.82	0.78	0.83	0.82	0.86	0.80	0.70	0.82	0.74	0.95	0.65	0.96	0.63	0.00	0.72	0.66	0.61	0.92	0.86
N	0.82	0.78	0.61	0.58	0.65	0.58	0.80	0.73	0.79	0.58	0.51	0.63	0.81	0.51	0.72	0.00	0.66	0.51	0.72	0.58
E	0.89	0.72	0.93	0.75	0.57	0.74	0.76	0.76	0.77	0.80	0.76	0.85	0.96	0.75	0.66	0.66	0.00	0.62	0.74	0.82
Q	0.80	0.52	0.62	0.57	0.56	0.64	0.57	0.53	0.64	0.55	0.67	0.42	0.86	0.37	0.61	0.51	0.62	0.00	0.43	0.34
K	0.87	0.63	0.92	0.71	0.63	0.76	0.75	0.69	0.71	0.66	0.82	0.63	0.86	0.60	0.92	0.72	0.74	0.43	0.00	0.43
R	0.92	0.71	0.70	0.70	0.71	0.75	0.69	0.65	0.68	0.58	0.66	0.58	0.95	0.55	0.86	0.58	0.82	0.34	0.43	0.00

Supporting table S5. Scaled distance matrix Z-Scales (3).

Residue	G	A	C	V	L	I	M	F	W	Y	H	T	P	S	D	N	E	Q	K	R
G	0.00	0.29	0.52	0.46	0.60	0.57	0.55	0.77	0.93	0.74	0.63	0.34	0.57	0.37	0.58	0.60	0.48	0.54	0.63	0.83
A	0.29	0.00	0.43	0.38	0.46	0.45	0.36	0.56	0.72	0.55	0.49	0.29	0.38	0.29	0.49	0.48	0.39	0.43	0.55	0.75
C	0.52	0.43	0.00	0.71	0.75	0.76	0.53	0.63	0.79	0.64	0.59	0.62	0.40	0.39	0.45	0.52	0.56	0.69	0.82	1.00
V	0.46	0.38	0.71	0.00	0.27	0.24	0.40	0.60	0.74	0.59	0.64	0.31	0.54	0.54	0.74	0.68	0.55	0.48	0.54	0.73
L	0.60	0.46	0.75	0.27	0.00	0.21	0.34	0.49	0.61	0.49	0.64	0.41	0.51	0.60	0.77	0.69	0.59	0.50	0.55	0.73
I	0.57	0.45	0.76	0.24	0.21	0.00	0.37	0.53	0.66	0.53	0.65	0.39	0.53	0.59	0.77	0.70	0.59	0.49	0.55	0.72
M	0.55	0.36	0.53	0.40	0.34	0.37	0.00	0.32	0.47	0.33	0.47	0.42	0.28	0.43	0.55	0.49	0.47	0.45	0.55	0.73
F	0.77	0.56	0.63	0.60	0.49	0.53	0.32	0.00	0.29	0.25	0.51	0.63	0.33	0.58	0.62	0.54	0.59	0.57	0.66	0.80
W	0.93	0.72	0.79	0.74	0.61	0.66	0.47	0.29	0.00	0.28	0.55	0.76	0.48	0.71	0.70	0.60	0.67	0.63	0.69	0.78
Y	0.74	0.55	0.64	0.59	0.49	0.53	0.33	0.25	0.28	0.00	0.40	0.58	0.34	0.53	0.55	0.45	0.49	0.46	0.55	0.66
H	0.63	0.49	0.59	0.64	0.64	0.65	0.47	0.51	0.55	0.40	0.00	0.50	0.43	0.37	0.32	0.23	0.27	0.32	0.40	0.50
T	0.34	0.29	0.62	0.31	0.41	0.39	0.42	0.63	0.76	0.58	0.50	0.00	0.50	0.37	0.57	0.52	0.37	0.34	0.41	0.61
P	0.57	0.38	0.40	0.54	0.51	0.53	0.28	0.33	0.48	0.34	0.43	0.50	0.00	0.38	0.44	0.42	0.45	0.50	0.63	0.79
S	0.37	0.29	0.39	0.54	0.60	0.59	0.43	0.58	0.71	0.53	0.37	0.37	0.38	0.00	0.32	0.34	0.29	0.40	0.52	0.70
D	0.58	0.49	0.45	0.74	0.77	0.77	0.55	0.62	0.70	0.55	0.32	0.57	0.44	0.32	0.00	0.25	0.33	0.48	0.58	0.70
N	0.60	0.48	0.52	0.68	0.69	0.70	0.49	0.54	0.60	0.45	0.23	0.52	0.42	0.34	0.25	0.00	0.28	0.39	0.48	0.59
E	0.48	0.39	0.56	0.55	0.59	0.59	0.47	0.59	0.67	0.49	0.27	0.37	0.45	0.29	0.33	0.28	0.00	0.27	0.36	0.51
Q	0.54	0.43	0.69	0.48	0.50	0.49	0.45	0.57	0.63	0.46	0.32	0.34	0.50	0.40	0.48	0.39	0.27	0.00	0.24	0.39
K	0.63	0.55	0.82	0.54	0.55	0.55	0.55	0.66	0.69	0.55	0.40	0.41	0.63	0.52	0.58	0.48	0.36	0.24	0.00	0.30
R	0.83	0.75	1.00	0.73	0.73	0.72	0.73	0.80	0.78	0.66	0.50	0.61	0.79	0.70	0.70	0.59	0.51	0.39	0.30	0.00

Supporting table S6. Scaled distance matrix Z-Scales (5).

Residue	G	A	C	V	L	I	M	F	W	Y	H	T	P	S	D	N	E	Q	K	R
G	0.00	0.39	0.60	0.42	0.57	0.52	0.57	0.70	0.96	0.69	0.74	0.31	0.66	0.38	0.58	0.63	0.49	0.51	0.62	0.80
A	0.39	0.00	0.75	0.41	0.42	0.44	0.54	0.60	0.88	0.67	0.63	0.40	0.37	0.30	0.50	0.45	0.52	0.42	0.54	0.75
C	0.60	0.75	0.00	0.78	0.88	0.84	0.57	0.65	0.80	0.61	0.79	0.68	0.84	0.66	0.73	0.85	0.71	0.84	0.89	1.00
V	0.42	0.41	0.78	0.00	0.28	0.22	0.48	0.56	0.83	0.59	0.75	0.29	0.61	0.50	0.69	0.67	0.54	0.44	0.54	0.71
L	0.57	0.42	0.88	0.28	0.00	0.21	0.50	0.51	0.79	0.59	0.75	0.43	0.51	0.54	0.71	0.63	0.61	0.45	0.55	0.73
I	0.52	0.44	0.84	0.22	0.21	0.00	0.48	0.52	0.79	0.56	0.75	0.37	0.58	0.54	0.72	0.67	0.58	0.45	0.54	0.71
M	0.57	0.54	0.57	0.48	0.50	0.48	0.00	0.32	0.46	0.36	0.50	0.52	0.56	0.58	0.72	0.69	0.66	0.58	0.54	0.67
F	0.70	0.60	0.65	0.56	0.51	0.52	0.32	0.00	0.42	0.27	0.58	0.60	0.52	0.59	0.67	0.63	0.64	0.58	0.62	0.73
W	0.96	0.88	0.80	0.83	0.79	0.79	0.46	0.42	0.00	0.43	0.57	0.86	0.78	0.88	0.94	0.88	0.91	0.83	0.72	0.75
Y	0.69	0.67	0.61	0.59	0.59	0.56	0.36	0.27	0.43	0.00	0.59	0.57	0.65	0.61	0.66	0.66	0.58	0.56	0.59	0.66
H	0.74	0.63	0.79	0.75	0.75	0.75	0.50	0.58	0.57	0.59	0.00	0.71	0.59	0.64	0.71	0.60	0.74	0.61	0.44	0.49
T	0.31	0.40	0.68	0.29	0.43	0.37	0.52	0.60	0.86	0.57	0.71	0.00	0.64	0.38	0.55	0.57	0.37	0.35	0.49	0.65
P	0.66	0.37	0.84	0.61	0.51	0.58	0.56	0.52	0.78	0.65	0.59	0.64	0.00	0.45	0.55	0.42	0.66	0.54	0.63	0.81
S	0.38	0.30	0.66	0.50	0.54	0.54	0.58	0.59	0.88	0.61	0.64	0.38	0.45	0.00	0.30	0.34	0.34	0.36	0.55	0.72
D	0.58	0.50	0.73	0.69	0.71	0.72	0.72	0.67	0.94	0.66	0.71	0.55	0.55	0.30	0.00	0.29	0.34	0.45	0.66	0.79
N	0.63	0.45	0.85	0.67	0.63	0.67	0.69	0.63	0.88	0.66	0.60	0.57	0.42	0.34	0.29	0.00	0.44	0.38	0.55	0.68
E	0.49	0.52	0.71	0.54	0.61	0.58	0.66	0.64	0.91	0.58	0.74	0.37	0.66	0.34	0.34	0.44	0.00	0.33	0.57	0.68
Q	0.51	0.42	0.84	0.44	0.45	0.45	0.58	0.58	0.83	0.56	0.61	0.35	0.54	0.36	0.45	0.38	0.33	0.00	0.36	0.51
K	0.62	0.54	0.89	0.54	0.55	0.54	0.54	0.62	0.72	0.59	0.44	0.49	0.63	0.55	0.66	0.55	0.57	0.36	0.00	0.28
R	0.80	0.75	1.00	0.71	0.73	0.71	0.67	0.73	0.75	0.66	0.49	0.65	0.81	0.72	0.79	0.68	0.68	0.51	0.28	0.00

Supporting table S7. Scaled distance matrix Z-Scales (Binned).

Residue	G	A	C	V	L	I	M	F	W	Y	H	T	P	S	D	N	E	Q	K	R
G	0.00	0.57	0.79	0.51	0.73	0.76	0.66	0.66	0.78	0.68	0.66	0.65	0.91	0.65	0.65	0.58	0.65	0.83	0.77	0.69
A	0.57	0.00	0.73	0.68	0.78	0.81	0.79	0.79	0.82	0.73	0.79	0.67	0.83	0.78	0.78	0.55	0.86	0.77	0.89	0.89
C	0.79	0.73	0.00	0.87	0.78	0.80	0.71	0.69	0.69	0.67	0.80	0.79	0.86	0.69	0.79	0.81	0.87	0.78	0.79	0.90
V	0.51	0.68	0.87	0.00	0.54	0.69	0.67	0.67	0.78	0.69	0.83	0.54	0.98	0.82	0.82	0.76	0.64	0.75	0.85	0.78
L	0.73	0.78	0.78	0.54	0.00	0.44	0.64	0.75	0.78	0.68	0.74	0.76	0.97	0.63	0.73	0.75	0.72	0.66	0.77	0.85
I	0.76	0.81	0.80	0.69	0.44	0.00	0.67	0.77	0.80	0.70	0.77	0.87	0.99	0.65	0.75	0.78	0.84	0.78	0.64	0.74
M	0.66	0.79	0.71	0.67	0.64	0.67	0.00	0.58	0.57	0.69	0.63	0.78	0.92	0.64	0.74	0.76	0.75	0.84	0.85	0.66
F	0.66	0.79	0.69	0.67	0.75	0.77	0.58	0.00	0.59	0.57	0.65	0.78	0.82	0.75	0.74	0.76	0.75	0.84	0.74	0.68
W	0.78	0.82	0.69	0.78	0.78	0.80	0.57	0.59	0.00	0.40	0.55	0.88	0.94	0.78	0.77	0.79	0.85	0.87	0.88	0.68
Y	0.68	0.73	0.67	0.69	0.68	0.70	0.69	0.57	0.40	0.00	0.67	0.87	0.93	0.76	0.75	0.70	0.84	0.85	0.86	0.78
H	0.66	0.79	0.80	0.83	0.74	0.77	0.63	0.65	0.55	0.67	0.00	0.85	0.91	0.56	0.56	0.68	0.75	0.76	0.70	0.55
T	0.65	0.67	0.79	0.54	0.76	0.87	0.78	0.78	0.88	0.87	0.85	0.00	1.00	0.75	0.75	0.86	0.55	0.53	0.88	0.81
P	0.91	0.83	0.86	0.98	0.97	0.99	0.92	0.82	0.94	0.93	0.91	1.00	0.00	0.91	0.83	0.71	0.90	0.92	0.83	1.00
S	0.65	0.78	0.69	0.82	0.63	0.65	0.64	0.75	0.78	0.76	0.56	0.75	0.91	0.00	0.39	0.67	0.63	0.65	0.69	0.78
D	0.65	0.78	0.79	0.82	0.73	0.75	0.74	0.74	0.77	0.75	0.56	0.75	0.83	0.39	0.00	0.56	0.51	0.54	0.58	0.77
N	0.58	0.55	0.81	0.76	0.75	0.78	0.76	0.76	0.79	0.70	0.68	0.86	0.71	0.67	0.56	0.00	0.67	0.77	0.70	0.79
E	0.65	0.86	0.87	0.64	0.72	0.84	0.75	0.75	0.85	0.84	0.75	0.55	0.90	0.63	0.51	0.67	0.00	0.53	0.69	0.70
Q	0.83	0.77	0.78	0.75	0.66	0.78	0.84	0.84	0.87	0.85	0.76	0.53	0.92	0.65	0.54	0.77	0.53	0.00	0.70	0.87
K	0.77	0.89	0.79	0.85	0.77	0.64	0.85	0.74	0.88	0.86	0.70	0.88	0.83	0.69	0.58	0.70	0.69	0.70	0.00	0.67
R	0.69	0.89	0.90	0.78	0.85	0.74	0.66	0.68	0.68	0.78	0.55	0.81	1.00	0.78	0.77	0.79	0.70	0.87	0.67	0.00

Supporting table S8. Scaled distance matrix VHSE.

Residue	G	A	C	V	L	I	M	F	W	Y	H	T	P	S	D	N	E	Q	K	R
G	0.00	0.58	0.80	0.81	0.75	0.86	0.75	0.92	1.00	0.90	0.75	0.77	0.91	0.67	0.83	0.59	0.68	0.71	0.78	0.93
A	0.58	0.00	0.47	0.30	0.37	0.39	0.41	0.56	0.75	0.64	0.50	0.32	0.63	0.31	0.64	0.46	0.54	0.43	0.63	0.80
C	0.80	0.47	0.00	0.52	0.56	0.59	0.34	0.46	0.71	0.58	0.48	0.34	0.71	0.31	0.61	0.49	0.64	0.41	0.69	0.78
V	0.81	0.30	0.52	0.00	0.29	0.20	0.40	0.49	0.69	0.62	0.57	0.33	0.61	0.43	0.73	0.61	0.63	0.50	0.71	0.85
L	0.75	0.37	0.56	0.29	0.00	0.22	0.32	0.37	0.50	0.46	0.54	0.41	0.65	0.48	0.71	0.55	0.58	0.46	0.62	0.76
I	0.86	0.39	0.59	0.20	0.22	0.00	0.40	0.44	0.60	0.56	0.60	0.40	0.63	0.51	0.76	0.64	0.65	0.53	0.70	0.84
M	0.75	0.41	0.34	0.40	0.32	0.40	0.00	0.26	0.46	0.37	0.43	0.35	0.67	0.38	0.63	0.45	0.57	0.34	0.58	0.69
F	0.92	0.56	0.46	0.49	0.37	0.44	0.26	0.00	0.33	0.28	0.50	0.43	0.68	0.51	0.66	0.56	0.63	0.43	0.63	0.69
W	1.00	0.75	0.71	0.69	0.50	0.60	0.46	0.33	0.00	0.23	0.63	0.66	0.84	0.72	0.80	0.65	0.73	0.55	0.64	0.66
Y	0.90	0.64	0.58	0.62	0.46	0.56	0.37	0.28	0.23	0.00	0.49	0.52	0.74	0.56	0.65	0.50	0.60	0.39	0.52	0.54
H	0.75	0.50	0.48	0.57	0.54	0.60	0.43	0.50	0.63	0.49	0.00	0.41	0.61	0.40	0.52	0.33	0.48	0.31	0.32	0.40
T	0.77	0.32	0.34	0.33	0.41	0.40	0.35	0.43	0.66	0.52	0.41	0.00	0.54	0.20	0.51	0.43	0.49	0.31	0.59	0.69
P	0.91	0.63	0.71	0.61	0.65	0.63	0.67	0.68	0.84	0.74	0.61	0.54	0.00	0.59	0.73	0.74	0.68	0.65	0.74	0.78
S	0.67	0.31	0.31	0.43	0.48	0.51	0.38	0.51	0.72	0.56	0.40	0.20	0.59	0.00	0.47	0.36	0.46	0.30	0.59	0.69
D	0.83	0.64	0.61	0.73	0.71	0.76	0.63	0.66	0.80	0.65	0.52	0.51	0.73	0.47	0.00	0.42	0.31	0.47	0.70	0.71
N	0.59	0.46	0.49	0.61	0.55	0.64	0.45	0.56	0.65	0.50	0.33	0.43	0.74	0.36	0.42	0.00	0.34	0.25	0.42	0.52
E	0.68	0.54	0.64	0.63	0.58	0.65	0.57	0.63	0.73	0.60	0.48	0.49	0.68	0.46	0.31	0.34	0.00	0.40	0.58	0.65
Q	0.71	0.43	0.41	0.50	0.46	0.53	0.34	0.43	0.55	0.39	0.31	0.31	0.65	0.30	0.47	0.25	0.40	0.00	0.40	0.49
K	0.78	0.63	0.69	0.71	0.62	0.70	0.58	0.63	0.64	0.52	0.32	0.59	0.74	0.59	0.70	0.42	0.58	0.40	0.00	0.26
R	0.93	0.80	0.78	0.85	0.76	0.84	0.69	0.69	0.66	0.54	0.40	0.69	0.78	0.69	0.71	0.52	0.65	0.49	0.26	0.00

Supporting table S9. Scaled distance matrix ST-Scales.

Residue	G	A	C	V	L	I	M	F	W	Y	H	T	P	S	D	N	E	Q	K	R
G	0.00	0.39	0.71	0.69	0.77	0.88	0.86	0.83	1.00	0.92	0.95	0.81	0.72	0.61	0.83	0.81	0.78	0.73	0.74	0.77
A	0.39	0.00	0.52	0.41	0.56	0.59	0.73	0.73	0.83	0.85	0.77	0.49	0.48	0.37	0.61	0.58	0.62	0.56	0.61	0.69
C	0.71	0.52	0.00	0.35	0.46	0.48	0.42	0.64	0.80	0.79	0.71	0.49	0.37	0.49	0.59	0.54	0.58	0.50	0.50	0.61
V	0.69	0.41	0.35	0.00	0.29	0.26	0.51	0.55	0.75	0.72	0.70	0.35	0.27	0.42	0.51	0.46	0.53	0.45	0.47	0.65
L	0.77	0.56	0.46	0.29	0.00	0.26	0.39	0.37	0.65	0.55	0.60	0.47	0.31	0.51	0.49	0.43	0.43	0.36	0.32	0.56
I	0.88	0.59	0.48	0.26	0.26	0.00	0.52	0.54	0.70	0.70	0.66	0.37	0.34	0.52	0.49	0.45	0.52	0.46	0.48	0.68
M	0.86	0.73	0.42	0.51	0.39	0.52	0.00	0.43	0.69	0.56	0.60	0.65	0.46	0.64	0.58	0.53	0.47	0.40	0.33	0.49
F	0.83	0.73	0.64	0.55	0.37	0.54	0.43	0.00	0.59	0.36	0.64	0.73	0.49	0.73	0.69	0.64	0.56	0.49	0.37	0.56
W	1.00	0.83	0.80	0.75	0.65	0.70	0.69	0.59	0.00	0.64	0.31	0.74	0.68	0.71	0.66	0.63	0.52	0.53	0.51	0.47
Y	0.92	0.85	0.79	0.72	0.55	0.70	0.56	0.36	0.64	0.00	0.69	0.78	0.63	0.77	0.67	0.64	0.57	0.55	0.55	0.68
H	0.95	0.77	0.71	0.70	0.60	0.66	0.60	0.64	0.31	0.69	0.00	0.67	0.63	0.60	0.54	0.51	0.38	0.42	0.45	0.40
T	0.81	0.49	0.49	0.35	0.47	0.37	0.65	0.73	0.74	0.78	0.67	0.00	0.42	0.30	0.34	0.33	0.49	0.46	0.61	0.74
P	0.72	0.48	0.37	0.27	0.31	0.34	0.46	0.49	0.68	0.63	0.63	0.42	0.00	0.45	0.54	0.49	0.52	0.46	0.45	0.61
S	0.61	0.37	0.49	0.42	0.51	0.52	0.64	0.73	0.71	0.77	0.60	0.30	0.45	0.00	0.32	0.31	0.40	0.39	0.55	0.62
D	0.83	0.61	0.59	0.51	0.49	0.49	0.58	0.69	0.66	0.67	0.54	0.34	0.54	0.32	0.00	0.15	0.28	0.31	0.53	0.62
N	0.81	0.58	0.54	0.46	0.43	0.45	0.53	0.64	0.63	0.64	0.51	0.33	0.49	0.31	0.15	0.00	0.25	0.26	0.47	0.58
E	0.78	0.62	0.58	0.53	0.43	0.52	0.47	0.56	0.52	0.57	0.38	0.49	0.52	0.40	0.28	0.25	0.00	0.17	0.35	0.42
Q	0.73	0.56	0.50	0.45	0.36	0.46	0.40	0.49	0.53	0.55	0.42	0.46	0.46	0.39	0.31	0.26	0.17	0.00	0.28	0.39
K	0.74	0.61	0.50	0.47	0.32	0.48	0.33	0.37	0.51	0.55	0.45	0.61	0.45	0.55	0.53	0.47	0.35	0.28	0.00	0.30
R	0.77	0.69	0.61	0.65	0.56	0.68	0.49	0.56	0.47	0.68	0.40	0.74	0.61	0.62	0.62	0.58	0.42	0.39	0.30	0.00

Supporting table S10. Scaled distance matrix T-Scales.

Residue	G	A	C	V	L	I	M	F	W	Y	H	T	P	S	D	N	E	Q	K	R
G	0.00	0.27	0.41	0.51	0.61	0.63	0.83	0.81	1.00	0.86	0.78	0.49	0.68	0.39	0.57	0.56	0.66	0.66	0.78	0.88
A	0.27	0.00	0.32	0.36	0.51	0.50	0.79	0.72	0.90	0.76	0.68	0.33	0.57	0.28	0.45	0.43	0.57	0.56	0.74	0.83
C	0.41	0.32	0.00	0.22	0.29	0.29	0.53	0.52	0.75	0.59	0.52	0.31	0.40	0.29	0.42	0.38	0.43	0.40	0.49	0.62
V	0.51	0.36	0.22	0.00	0.26	0.23	0.55	0.51	0.71	0.56	0.51	0.25	0.40	0.31	0.38	0.34	0.40	0.38	0.52	0.63
L	0.61	0.51	0.29	0.26	0.00	0.18	0.38	0.49	0.73	0.56	0.54	0.40	0.47	0.45	0.48	0.43	0.41	0.37	0.36	0.50
I	0.63	0.50	0.29	0.23	0.18	0.00	0.43	0.47	0.69	0.54	0.52	0.37	0.43	0.44	0.46	0.41	0.41	0.37	0.42	0.55
M	0.83	0.79	0.53	0.55	0.38	0.43	0.00	0.60	0.85	0.71	0.71	0.72	0.62	0.73	0.78	0.73	0.64	0.59	0.31	0.52
F	0.81	0.72	0.52	0.51	0.49	0.47	0.60	0.00	0.34	0.22	0.22	0.53	0.39	0.55	0.53	0.50	0.42	0.40	0.47	0.50
W	1.00	0.90	0.75	0.71	0.73	0.69	0.85	0.34	0.00	0.30	0.35	0.70	0.54	0.74	0.69	0.68	0.63	0.62	0.74	0.73
Y	0.86	0.76	0.59	0.56	0.56	0.54	0.71	0.22	0.30	0.00	0.22	0.54	0.49	0.57	0.50	0.48	0.40	0.39	0.56	0.52
H	0.78	0.68	0.52	0.51	0.54	0.52	0.71	0.22	0.35	0.22	0.00	0.48	0.38	0.50	0.47	0.45	0.40	0.40	0.57	0.58
T	0.49	0.33	0.31	0.25	0.40	0.37	0.72	0.53	0.70	0.54	0.48	0.00	0.47	0.22	0.24	0.22	0.35	0.35	0.62	0.67
P	0.68	0.57	0.40	0.40	0.47	0.43	0.62	0.39	0.54	0.49	0.38	0.47	0.00	0.48	0.57	0.54	0.56	0.54	0.61	0.74
S	0.39	0.28	0.29	0.31	0.45	0.44	0.73	0.55	0.74	0.57	0.50	0.22	0.48	0.00	0.28	0.26	0.38	0.39	0.62	0.68
D	0.57	0.45	0.42	0.38	0.48	0.46	0.78	0.53	0.69	0.50	0.47	0.24	0.57	0.28	0.00	0.16	0.27	0.29	0.62	0.61
N	0.56	0.43	0.38	0.34	0.43	0.41	0.73	0.50	0.68	0.48	0.45	0.22	0.54	0.26	0.16	0.00	0.25	0.26	0.58	0.58
E	0.66	0.57	0.43	0.40	0.41	0.41	0.64	0.42	0.63	0.40	0.40	0.35	0.56	0.38	0.27	0.25	0.00	0.16	0.45	0.41
Q	0.66	0.56	0.40	0.38	0.37	0.37	0.59	0.40	0.62	0.39	0.40	0.35	0.54	0.39	0.29	0.26	0.16	0.00	0.40	0.38
K	0.78	0.74	0.49	0.52	0.36	0.42	0.31	0.47	0.74	0.56	0.57	0.62	0.61	0.62	0.62	0.58	0.45	0.40	0.00	0.28
R	0.88	0.83	0.62	0.63	0.50	0.55	0.52	0.50	0.73	0.52	0.58	0.67	0.74	0.68	0.61	0.58	0.41	0.38	0.28	0.00

Supporting table S11. Scaled distance matrix MSWHIM.

Residue	G	A	C	V	L	I	M	F	W	Y	H	T	P	S	D	N	E	Q	K	R
G	0.00	0.31	0.38	0.52	0.53	0.56	0.59	0.62	0.69	0.64	0.56	0.50	0.45	0.45	0.39	0.32	0.41	0.61	0.63	0.80
A	0.31	0.00	0.26	0.32	0.35	0.36	0.40	0.59	0.67	0.64	0.58	0.35	0.31	0.31	0.60	0.34	0.50	0.52	0.56	0.74
C	0.38	0.26	0.00	0.34	0.28	0.31	0.36	0.55	0.64	0.58	0.59	0.41	0.32	0.40	0.66	0.37	0.45	0.47	0.43	0.60
V	0.52	0.32	0.34	0.00	0.29	0.25	0.26	0.61	0.67	0.69	0.63	0.28	0.27	0.29	0.83	0.48	0.68	0.48	0.63	0.77
L	0.53	0.35	0.28	0.29	0.00	0.22	0.24	0.53	0.61	0.58	0.60	0.38	0.29	0.41	0.83	0.46	0.58	0.41	0.46	0.59
I	0.56	0.36	0.31	0.25	0.22	0.00	0.23	0.57	0.65	0.64	0.64	0.36	0.30	0.39	0.87	0.50	0.64	0.44	0.53	0.65
M	0.59	0.40	0.36	0.26	0.24	0.23	0.00	0.51	0.58	0.59	0.58	0.33	0.27	0.39	0.90	0.48	0.67	0.38	0.57	0.68
F	0.62	0.59	0.55	0.61	0.53	0.57	0.51	0.00	0.23	0.24	0.29	0.53	0.45	0.61	0.83	0.41	0.57	0.26	0.68	0.72
W	0.69	0.67	0.64	0.67	0.61	0.65	0.58	0.23	0.00	0.27	0.28	0.57	0.51	0.67	0.88	0.47	0.65	0.31	0.78	0.81
Y	0.64	0.64	0.58	0.69	0.58	0.64	0.59	0.24	0.27	0.00	0.34	0.62	0.53	0.70	0.81	0.44	0.52	0.33	0.65	0.67
H	0.56	0.58	0.59	0.63	0.60	0.64	0.58	0.29	0.28	0.34	0.00	0.50	0.46	0.57	0.73	0.36	0.59	0.36	0.79	0.87
T	0.50	0.35	0.41	0.28	0.38	0.36	0.33	0.53	0.57	0.62	0.50	0.00	0.25	0.24	0.79	0.41	0.69	0.43	0.73	0.87
P	0.45	0.31	0.32	0.27	0.29	0.30	0.27	0.45	0.51	0.53	0.46	0.25	0.00	0.30	0.75	0.35	0.58	0.35	0.61	0.74
S	0.45	0.31	0.40	0.29	0.41	0.39	0.39	0.61	0.67	0.70	0.57	0.24	0.30	0.00	0.73	0.42	0.69	0.53	0.75	0.91
D	0.39	0.60	0.66	0.83	0.83	0.87	0.90	0.83	0.88	0.81	0.73	0.79	0.75	0.73	0.00	0.53	0.50	0.86	0.84	1.00
N	0.32	0.34	0.37	0.48	0.46	0.50	0.48	0.41	0.47	0.44	0.36	0.41	0.35	0.42	0.53	0.00	0.41	0.41	0.61	0.75
E	0.41	0.50	0.45	0.68	0.58	0.64	0.67	0.57	0.65	0.52	0.59	0.69	0.58	0.69	0.50	0.41	0.00	0.60	0.46	0.57
Q	0.61	0.52	0.47	0.48	0.41	0.44	0.38	0.26	0.31	0.33	0.36	0.43	0.35	0.53	0.86	0.41	0.60	0.00	0.62	0.68
K	0.63	0.56	0.43	0.63	0.46	0.53	0.57	0.68	0.78	0.65	0.79	0.73	0.61	0.75	0.84	0.61	0.46	0.62	0.00	0.29
R	0.80	0.74	0.60	0.77	0.59	0.65	0.68	0.72	0.81	0.67	0.87	0.87	0.74	0.91	1.00	0.75	0.57	0.68	0.29	0.00

Supporting table S12. Scaled distance matrix FASGAI.

Residue	G	A	C	V	L	I	M	F	W	Y	H	T	P	S	D	N	E	Q	K	R
G	0.00	0.57	0.75	0.72	0.79	0.75	0.82	0.77	0.86	0.72	0.74	0.53	0.87	0.40	0.50	0.46	0.73	0.67	0.74	0.92
A	0.57	0.00	0.66	0.41	0.39	0.45	0.48	0.52	0.73	0.66	0.53	0.37	0.84	0.37	0.48	0.50	0.40	0.45	0.50	0.72
C	0.75	0.66	0.00	0.60	0.81	0.62	0.47	0.60	0.62	0.63	0.39	0.50	0.97	0.57	0.65	0.54	0.72	0.51	0.66	0.70
V	0.72	0.41	0.60	0.00	0.35	0.23	0.44	0.36	0.60	0.52	0.52	0.36	0.94	0.49	0.70	0.60	0.64	0.47	0.49	0.57
L	0.79	0.39	0.81	0.35	0.00	0.31	0.50	0.40	0.67	0.62	0.64	0.51	0.91	0.58	0.71	0.69	0.58	0.58	0.58	0.75
I	0.75	0.45	0.62	0.23	0.31	0.00	0.38	0.24	0.48	0.43	0.50	0.42	0.92	0.53	0.68	0.60	0.63	0.49	0.52	0.63
M	0.82	0.48	0.47	0.44	0.50	0.38	0.00	0.33	0.43	0.50	0.31	0.46	0.90	0.56	0.58	0.54	0.48	0.39	0.52	0.64
F	0.77	0.52	0.60	0.36	0.40	0.24	0.33	0.00	0.34	0.34	0.43	0.44	0.87	0.55	0.63	0.55	0.60	0.45	0.53	0.64
W	0.86	0.73	0.62	0.60	0.67	0.48	0.43	0.34	0.00	0.29	0.45	0.60	0.99	0.69	0.66	0.57	0.70	0.53	0.63	0.71
Y	0.72	0.66	0.63	0.52	0.62	0.43	0.50	0.34	0.29	0.00	0.45	0.47	0.92	0.56	0.61	0.47	0.69	0.45	0.51	0.59
H	0.74	0.53	0.39	0.52	0.64	0.50	0.31	0.43	0.45	0.45	0.00	0.38	0.82	0.46	0.47	0.38	0.46	0.25	0.41	0.51
T	0.53	0.37	0.50	0.36	0.51	0.42	0.46	0.44	0.60	0.47	0.38	0.00	0.73	0.23	0.47	0.35	0.51	0.30	0.37	0.50
P	0.87	0.84	0.97	0.94	0.91	0.92	0.90	0.87	0.99	0.92	0.82	0.73	0.00	0.70	0.76	0.76	0.82	0.80	0.90	1.00
S	0.40	0.37	0.57	0.49	0.58	0.53	0.56	0.55	0.69	0.56	0.46	0.23	0.70	0.00	0.38	0.29	0.49	0.36	0.44	0.61
D	0.50	0.48	0.65	0.70	0.71	0.68	0.58	0.63	0.66	0.61	0.47	0.47	0.76	0.38	0.00	0.27	0.37	0.43	0.55	0.78
N	0.46	0.50	0.54	0.60	0.69	0.60	0.54	0.55	0.57	0.47	0.38	0.35	0.76	0.29	0.27	0.00	0.47	0.33	0.45	0.63
E	0.73	0.40	0.72	0.64	0.58	0.63	0.48	0.60	0.70	0.69	0.46	0.51	0.82	0.49	0.37	0.47	0.00	0.39	0.47	0.72
Q	0.67	0.45	0.51	0.47	0.58	0.49	0.39	0.45	0.53	0.45	0.25	0.30	0.80	0.36	0.43	0.33	0.39	0.00	0.26	0.42
K	0.74	0.50	0.66	0.49	0.58	0.52	0.52	0.53	0.63	0.51	0.41	0.37	0.90	0.44	0.55	0.45	0.47	0.26	0.00	0.32
R	0.92	0.72	0.70	0.57	0.75	0.63	0.64	0.64	0.71	0.59	0.51	0.50	1.00	0.61	0.78	0.63	0.72	0.42	0.32	0.00

Supporting table S13. Scaled distance matrix BLOSUM.

Residue	G	A	C	V	L	I	M	F	W	Y	H	T	P	S	D	N	E	Q	K	R
G	0.00	0.66	0.99	0.75	0.77	0.77	0.77	0.85	0.89	0.87	0.89	0.97	0.95	0.70	0.84	0.77	0.84	0.82	0.82	0.79
A	0.66	0.00	0.81	0.52	0.66	0.64	0.68	0.77	0.90	0.65	0.81	0.70	0.87	0.43	0.85	0.88	0.61	0.59	0.66	0.70
C	0.99	0.81	0.00	0.84	0.78	0.81	0.81	0.92	0.96	0.87	0.92	0.93	1.00	0.78	0.87	0.91	0.89	0.84	0.85	0.83
V	0.75	0.52	0.84	0.00	0.36	0.26	0.39	0.72	0.83	0.64	0.73	0.66	0.79	0.55	0.72	0.75	0.57	0.53	0.70	0.72
L	0.77	0.66	0.78	0.36	0.00	0.27	0.23	0.62	0.78	0.64	0.77	0.70	0.80	0.60	0.73	0.64	0.66	0.57	0.56	0.54
I	0.77	0.64	0.81	0.26	0.27	0.00	0.35	0.63	0.85	0.64	0.75	0.71	0.82	0.60	0.67	0.67	0.63	0.61	0.69	0.70
M	0.77	0.68	0.81	0.39	0.23	0.35	0.00	0.73	0.72	0.69	0.74	0.72	0.83	0.64	0.75	0.64	0.63	0.49	0.57	0.52
F	0.85	0.77	0.92	0.72	0.62	0.63	0.73	0.00	0.88	0.45	0.91	0.84	0.93	0.66	0.74	0.80	0.76	0.83	0.65	0.73
W	0.89	0.90	0.96	0.83	0.78	0.85	0.72	0.88	0.00	0.78	0.95	0.84	0.98	0.82	0.91	0.90	0.82	0.73	0.86	0.86
Y	0.87	0.65	0.87	0.64	0.64	0.64	0.69	0.45	0.78	0.00	0.60	0.76	0.85	0.60	0.79	0.80	0.65	0.66	0.67	0.69
H	0.89	0.81	0.92	0.73	0.77	0.75	0.74	0.91	0.95	0.60	0.00	0.90	0.92	0.76	0.90	0.71	0.77	0.67	0.86	0.77
T	0.97	0.70	0.93	0.66	0.70	0.71	0.72	0.84	0.84	0.76	0.90	0.00	0.88	0.42	0.85	0.67	0.77	0.74	0.76	0.80
P	0.95	0.87	1.00	0.79	0.80	0.82	0.83	0.93	0.98	0.85	0.92	0.88	0.00	0.84	0.88	0.93	0.87	0.85	0.80	0.85
S	0.70	0.43	0.78	0.55	0.60	0.60	0.64	0.66	0.82	0.60	0.76	0.42	0.84	0.00	0.68	0.59	0.57	0.57	0.56	0.61
D	0.84	0.85	0.87	0.72	0.73	0.67	0.75	0.74	0.91	0.79	0.90	0.85	0.88	0.68	0.00	0.69	0.49	0.68	0.75	0.85
N	0.77	0.88	0.91	0.75	0.64	0.67	0.64	0.80	0.90	0.80	0.71	0.67	0.93	0.59	0.69	0.00	0.80	0.75	0.71	0.65
E	0.84	0.61	0.89	0.57	0.66	0.63	0.63	0.76	0.82	0.65	0.77	0.77	0.87	0.57	0.49	0.80	0.00	0.33	0.59	0.68
Q	0.82	0.59	0.84	0.53	0.57	0.61	0.49	0.83	0.73	0.66	0.67	0.74	0.85	0.57	0.68	0.75	0.33	0.00	0.53	0.54
K	0.82	0.66	0.85	0.70	0.56	0.69	0.57	0.65	0.86	0.67	0.86	0.76	0.80	0.56	0.75	0.71	0.59	0.53	0.00	0.27
R	0.79	0.70	0.83	0.72	0.54	0.70	0.52	0.73	0.86	0.69	0.77	0.80	0.85	0.61	0.85	0.65	0.68	0.54	0.27	0.00

Supporting table S14. Scaled distance matrix ProtFP_PCA using 2 PCs.

Residue	A	R	Q	F	Y	W	S	H	G	K	P	E	D	T	C	M	L	I	N	V
A	0.00	0.64	0.47	0.49	0.50	0.66	0.27	0.38	0.35	0.59	0.24	0.47	0.47	0.20	0.28	0.39	0.38	0.40	0.40	0.30
R	0.64	0.00	0.21	0.60	0.36	0.56	0.51	0.29	0.84	0.14	0.48	0.27	0.37	0.45	0.68	0.54	0.63	0.67	0.34	0.66
Q	0.47	0.21	0.00	0.59	0.39	0.62	0.30	0.23	0.63	0.13	0.28	0.07	0.18	0.27	0.58	0.51	0.57	0.62	0.13	0.58
F	0.49	0.60	0.59	0.00	0.24	0.20	0.65	0.36	0.84	0.67	0.59	0.65	0.73	0.50	0.26	0.11	0.13	0.14	0.64	0.23
Y	0.50	0.36	0.39	0.24	0.00	0.23	0.54	0.18	0.81	0.44	0.49	0.46	0.55	0.41	0.39	0.21	0.30	0.34	0.47	0.36
W	0.66	0.56	0.62	0.20	0.23	0.00	0.76	0.41	1.00	0.67	0.71	0.69	0.79	0.62	0.46	0.27	0.33	0.35	0.70	0.43
S	0.27	0.51	0.30	0.65	0.54	0.76	0.00	0.37	0.33	0.41	0.06	0.26	0.22	0.15	0.51	0.54	0.57	0.60	0.18	0.52
H	0.38	0.29	0.23	0.36	0.18	0.41	0.37	0.00	0.66	0.32	0.32	0.29	0.38	0.24	0.39	0.28	0.36	0.40	0.29	0.38
G	0.35	0.84	0.63	0.84	0.81	1.00	0.33	0.66	0.00	0.74	0.36	0.58	0.52	0.41	0.62	0.74	0.73	0.74	0.51	0.65
K	0.59	0.14	0.13	0.67	0.44	0.67	0.41	0.32	0.74	0.00	0.40	0.15	0.24	0.40	0.69	0.59	0.67	0.71	0.23	0.68
P	0.24	0.48	0.28	0.59	0.49	0.71	0.06	0.32	0.36	0.40	0.00	0.25	0.24	0.09	0.46	0.49	0.52	0.55	0.18	0.47
E	0.47	0.27	0.07	0.65	0.46	0.69	0.26	0.29	0.58	0.15	0.25	0.00	0.11	0.27	0.62	0.56	0.62	0.66	0.08	0.61
D	0.47	0.37	0.18	0.73	0.55	0.79	0.22	0.38	0.52	0.24	0.24	0.11	0.00	0.29	0.66	0.63	0.68	0.72	0.09	0.66
T	0.20	0.45	0.27	0.50	0.41	0.62	0.15	0.24	0.41	0.40	0.09	0.27	0.29	0.00	0.38	0.39	0.43	0.46	0.21	0.39
C	0.28	0.68	0.58	0.26	0.39	0.46	0.51	0.39	0.62	0.69	0.46	0.62	0.66	0.38	0.00	0.20	0.13	0.13	0.58	0.03
M	0.39	0.54	0.51	0.11	0.21	0.27	0.54	0.28	0.74	0.59	0.49	0.56	0.63	0.39	0.20	0.00	0.09	0.13	0.54	0.17
L	0.38	0.63	0.57	0.13	0.30	0.33	0.57	0.36	0.73	0.67	0.52	0.62	0.68	0.43	0.13	0.09	0.00	0.04	0.60	0.10
I	0.40	0.67	0.62	0.14	0.34	0.35	0.60	0.40	0.74	0.71	0.55	0.66	0.72	0.46	0.13	0.13	0.04	0.00	0.63	0.10
N	0.40	0.34	0.13	0.64	0.47	0.70	0.18	0.29	0.51	0.23	0.18	0.08	0.09	0.21	0.58	0.54	0.60	0.63	0.00	0.58
V	0.30	0.66	0.58	0.23	0.36	0.43	0.52	0.38	0.65	0.68	0.47	0.61	0.66	0.39	0.03	0.17	0.10	0.10	0.58	0.00

Supporting table S15. Scaled distance matrix Z-Scales using 2 PCs.

Residue	A	R	Q	F	Y	W	S	H	G	K	P	E	D	T	C	M	L	I	N	V
A	0.00	0.57	0.31	0.60	0.54	0.76	0.24	0.47	0.24	0.37	0.31	0.38	0.48	0.05	0.09	0.36	0.45	0.41	0.47	0.28
R	0.57	0.00	0.26	0.76	0.59	0.78	0.37	0.12	0.66	0.20	0.55	0.22	0.16	0.53	0.48	0.68	0.85	0.83	0.10	0.78
Q	0.31	0.26	0.00	0.60	0.46	0.68	0.17	0.16	0.45	0.06	0.33	0.13	0.22	0.28	0.23	0.45	0.61	0.59	0.17	0.52
F	0.60	0.76	0.60	0.00	0.17	0.20	0.71	0.65	0.85	0.64	0.30	0.73	0.81	0.63	0.61	0.25	0.32	0.36	0.71	0.47
Y	0.54	0.59	0.46	0.17	0.00	0.23	0.59	0.49	0.78	0.49	0.23	0.59	0.65	0.55	0.52	0.26	0.40	0.43	0.55	0.50
W	0.76	0.78	0.68	0.20	0.23	0.00	0.82	0.69	1.00	0.71	0.44	0.81	0.86	0.78	0.75	0.43	0.51	0.56	0.76	0.66
S	0.24	0.37	0.17	0.71	0.59	0.82	0.00	0.29	0.29	0.19	0.42	0.15	0.25	0.19	0.16	0.52	0.65	0.62	0.27	0.51
H	0.47	0.12	0.16	0.65	0.49	0.69	0.29	0.00	0.59	0.10	0.43	0.18	0.18	0.44	0.39	0.56	0.73	0.72	0.07	0.67
G	0.24	0.66	0.45	0.85	0.78	1.00	0.29	0.59	0.00	0.48	0.56	0.43	0.52	0.22	0.26	0.61	0.67	0.62	0.56	0.47
K	0.37	0.20	0.06	0.64	0.49	0.71	0.19	0.10	0.48	0.00	0.39	0.10	0.16	0.34	0.29	0.51	0.68	0.65	0.10	0.59
P	0.31	0.55	0.33	0.30	0.23	0.44	0.42	0.43	0.56	0.39	0.00	0.47	0.55	0.34	0.31	0.13	0.30	0.29	0.48	0.30
E	0.38	0.22	0.13	0.73	0.59	0.81	0.15	0.18	0.43	0.10	0.47	0.00	0.11	0.33	0.29	0.58	0.74	0.71	0.13	0.62
D	0.48	0.16	0.22	0.81	0.65	0.86	0.25	0.18	0.52	0.16	0.55	0.11	0.00	0.44	0.40	0.68	0.83	0.81	0.11	0.73
T	0.05	0.53	0.28	0.63	0.55	0.78	0.19	0.44	0.22	0.34	0.34	0.33	0.44	0.00	0.05	0.40	0.50	0.45	0.43	0.33
C	0.09	0.48	0.23	0.61	0.52	0.75	0.16	0.39	0.26	0.29	0.31	0.29	0.40	0.05	0.00	0.39	0.50	0.46	0.39	0.35
M	0.36	0.68	0.45	0.25	0.26	0.43	0.52	0.56	0.61	0.51	0.13	0.58	0.68	0.40	0.39	0.00	0.17	0.18	0.60	0.24
L	0.45	0.85	0.61	0.32	0.40	0.51	0.65	0.73	0.67	0.68	0.30	0.74	0.83	0.50	0.50	0.17	0.00	0.06	0.77	0.21
I	0.41	0.83	0.59	0.36	0.43	0.56	0.62	0.72	0.62	0.65	0.29	0.71	0.81	0.45	0.46	0.18	0.06	0.00	0.75	0.15
N	0.47	0.10	0.17	0.71	0.55	0.76	0.27	0.07	0.56	0.10	0.48	0.13	0.11	0.43	0.39	0.60	0.77	0.75	0.00	0.69
V	0.28	0.78	0.52	0.47	0.50	0.66	0.51	0.67	0.47	0.59	0.30	0.62	0.73	0.33	0.35	0.24	0.21	0.15	0.69	0.00

Supporting table S16. Scaled distance matrix VHSE using 2 PCs.

Residue	A	R	Q	F	Y	W	S	H	G	K	P	E	D	T	C	M	L	I	N	V
A	0.00	0.76	0.42	0.55	0.69	0.87	0.22	0.26	0.14	0.56	0.24	0.51	0.55	0.20	0.14	0.26	0.43	0.37	0.40	0.16
R	0.76	0.00	0.36	0.78	0.53	0.76	0.62	0.50	0.82	0.20	0.59	0.27	0.21	0.57	0.89	0.80	0.79	0.80	0.38	0.82
Q	0.42	0.36	0.00	0.64	0.54	0.79	0.26	0.16	0.46	0.16	0.31	0.09	0.15	0.22	0.53	0.52	0.58	0.57	0.03	0.51
F	0.55	0.78	0.64	0.00	0.34	0.37	0.66	0.54	0.69	0.68	0.38	0.68	0.67	0.55	0.67	0.31	0.14	0.20	0.65	0.43
Y	0.69	0.53	0.54	0.34	0.00	0.25	0.70	0.53	0.81	0.50	0.46	0.54	0.50	0.58	0.83	0.55	0.43	0.47	0.57	0.64
W	0.87	0.76	0.79	0.37	0.25	0.00	0.92	0.76	1.00	0.75	0.65	0.79	0.75	0.80	1.00	0.66	0.50	0.56	0.81	0.77
S	0.22	0.62	0.26	0.66	0.70	0.92	0.00	0.17	0.21	0.41	0.28	0.34	0.40	0.12	0.30	0.43	0.56	0.52	0.23	0.36
H	0.26	0.50	0.16	0.54	0.53	0.76	0.17	0.00	0.33	0.30	0.16	0.25	0.29	0.07	0.39	0.37	0.46	0.43	0.15	0.34
G	0.14	0.82	0.46	0.69	0.81	1.00	0.21	0.33	0.00	0.61	0.36	0.54	0.60	0.26	0.10	0.40	0.56	0.51	0.43	0.29
K	0.56	0.20	0.16	0.68	0.50	0.75	0.41	0.30	0.61	0.00	0.41	0.08	0.01	0.37	0.69	0.63	0.66	0.65	0.18	0.63
P	0.24	0.59	0.31	0.38	0.46	0.65	0.28	0.16	0.36	0.41	0.00	0.38	0.40	0.16	0.38	0.22	0.29	0.26	0.31	0.23
E	0.51	0.27	0.09	0.68	0.54	0.79	0.34	0.25	0.54	0.08	0.38	0.00	0.07	0.31	0.62	0.60	0.64	0.63	0.11	0.59
D	0.55	0.21	0.15	0.67	0.50	0.75	0.40	0.29	0.60	0.01	0.40	0.07	0.00	0.36	0.68	0.62	0.65	0.64	0.17	0.63
T	0.20	0.57	0.22	0.55	0.58	0.80	0.12	0.07	0.26	0.37	0.16	0.31	0.36	0.00	0.32	0.34	0.45	0.42	0.21	0.30
C	0.14	0.89	0.53	0.67	0.83	1.00	0.30	0.39	0.10	0.69	0.38	0.62	0.68	0.32	0.00	0.36	0.53	0.47	0.51	0.24
M	0.26	0.80	0.52	0.31	0.55	0.66	0.43	0.37	0.40	0.63	0.22	0.60	0.62	0.34	0.36	0.00	0.17	0.12	0.52	0.12
L	0.43	0.79	0.58	0.14	0.43	0.50	0.56	0.46	0.56	0.66	0.29	0.64	0.65	0.45	0.53	0.17	0.00	0.06	0.59	0.29
I	0.37	0.80	0.57	0.20	0.47	0.56	0.52	0.43	0.51	0.65	0.26	0.63	0.64	0.42	0.47	0.12	0.06	0.00	0.57	0.23
N	0.40	0.38	0.03	0.65	0.57	0.81	0.23	0.15	0.43	0.18	0.31	0.11	0.17	0.21	0.51	0.52	0.59	0.57	0.00	0.50
V	0.16	0.82	0.51	0.43	0.64	0.77	0.36	0.34	0.29	0.63	0.23	0.59	0.63	0.30	0.24	0.12	0.29	0.23	0.50	0.00

Supporting table S17. Scaled distance matrix ST-Scales using 2 PCs.

Residue	A	R	Q	F	Y	W	S	H	G	K	P	E	D	T	C	M	L	I	N	V
A	0.00	0.79	0.51	0.64	0.85	0.94	0.19	0.65	0.31	0.55	0.23	0.56	0.36	0.19	0.18	0.57	0.31	0.30	0.37	0.16
R	0.79	0.00	0.28	0.26	0.15	0.42	0.61	0.15	0.72	0.24	0.56	0.25	0.43	0.72	0.62	0.25	0.50	0.70	0.42	0.72
Q	0.51	0.28	0.00	0.24	0.37	0.55	0.33	0.16	0.46	0.04	0.28	0.06	0.15	0.46	0.34	0.10	0.24	0.46	0.14	0.46
F	0.64	0.26	0.24	0.00	0.24	0.32	0.51	0.15	0.68	0.20	0.41	0.26	0.33	0.52	0.49	0.31	0.33	0.48	0.32	0.53
Y	0.85	0.15	0.37	0.24	0.00	0.28	0.69	0.21	0.83	0.32	0.62	0.35	0.51	0.75	0.69	0.37	0.55	0.71	0.50	0.76
W	0.94	0.42	0.55	0.32	0.28	0.00	0.82	0.42	1.00	0.51	0.72	0.56	0.65	0.79	0.81	0.60	0.64	0.72	0.65	0.81
S	0.19	0.61	0.33	0.51	0.69	0.82	0.00	0.48	0.22	0.37	0.11	0.37	0.19	0.25	0.04	0.38	0.19	0.33	0.19	0.23
H	0.65	0.15	0.16	0.15	0.21	0.42	0.48	0.00	0.62	0.11	0.42	0.15	0.30	0.57	0.48	0.18	0.35	0.55	0.29	0.57
G	0.31	0.72	0.46	0.68	0.83	1.00	0.22	0.62	0.00	0.51	0.33	0.48	0.35	0.45	0.25	0.47	0.40	0.54	0.35	0.42
K	0.55	0.24	0.04	0.20	0.32	0.51	0.37	0.11	0.51	0.00	0.32	0.07	0.19	0.48	0.37	0.12	0.26	0.48	0.18	0.48
P	0.23	0.56	0.28	0.41	0.62	0.72	0.11	0.42	0.33	0.32	0.00	0.33	0.14	0.19	0.08	0.36	0.08	0.24	0.14	0.18
E	0.56	0.25	0.06	0.26	0.35	0.56	0.37	0.15	0.48	0.07	0.33	0.00	0.19	0.51	0.38	0.05	0.29	0.52	0.19	0.51
D	0.36	0.43	0.15	0.33	0.51	0.65	0.19	0.30	0.35	0.19	0.14	0.19	0.00	0.33	0.19	0.22	0.12	0.36	0.01	0.32
T	0.19	0.72	0.46	0.52	0.75	0.79	0.25	0.57	0.45	0.48	0.19	0.51	0.33	0.00	0.21	0.55	0.22	0.11	0.33	0.03
C	0.18	0.62	0.34	0.49	0.69	0.81	0.04	0.48	0.25	0.37	0.08	0.38	0.19	0.21	0.00	0.40	0.17	0.29	0.19	0.19
M	0.57	0.25	0.10	0.31	0.37	0.60	0.38	0.18	0.47	0.12	0.36	0.05	0.22	0.55	0.40	0.00	0.33	0.56	0.22	0.54
L	0.31	0.50	0.24	0.33	0.55	0.64	0.19	0.35	0.40	0.26	0.08	0.29	0.12	0.22	0.17	0.33	0.00	0.23	0.12	0.22
I	0.30	0.70	0.46	0.48	0.71	0.72	0.33	0.55	0.54	0.48	0.24	0.52	0.36	0.11	0.29	0.56	0.23	0.00	0.36	0.14
N	0.37	0.42	0.14	0.32	0.50	0.65	0.19	0.29	0.35	0.18	0.14	0.19	0.01	0.33	0.19	0.22	0.12	0.36	0.00	0.32
V	0.16	0.72	0.46	0.53	0.76	0.81	0.23	0.57	0.42	0.48	0.18	0.51	0.32	0.03	0.19	0.54	0.22	0.14	0.32	0.00

Supporting table S18. Scaled distance matrix T-Scales using 2 PCs.

Residue	A	R	Q	F	Y	W	S	H	G	K	P	E	D	T	C	M	L	I	N	V
A	0.00	0.66	0.42	0.59	0.69	0.91	0.12	0.49	0.09	0.47	0.27	0.43	0.31	0.20	0.12	0.35	0.31	0.31	0.31	0.20
R	0.66	0.00	0.24	0.29	0.29	0.52	0.54	0.33	0.73	0.20	0.56	0.24	0.35	0.47	0.55	0.32	0.36	0.37	0.36	0.47
Q	0.42	0.24	0.00	0.27	0.34	0.60	0.31	0.22	0.50	0.05	0.35	0.01	0.12	0.23	0.31	0.08	0.12	0.14	0.12	0.24
F	0.59	0.29	0.27	0.00	0.10	0.34	0.48	0.09	0.68	0.27	0.38	0.27	0.33	0.39	0.48	0.30	0.31	0.29	0.33	0.39
Y	0.69	0.29	0.34	0.10	0.00	0.26	0.58	0.20	0.77	0.33	0.48	0.34	0.42	0.49	0.58	0.39	0.40	0.39	0.41	0.49
W	0.91	0.52	0.60	0.34	0.26	0.00	0.81	0.42	1.00	0.59	0.66	0.60	0.67	0.72	0.80	0.64	0.64	0.63	0.66	0.71
S	0.12	0.54	0.31	0.48	0.58	0.81	0.00	0.39	0.20	0.35	0.23	0.31	0.19	0.09	0.01	0.23	0.19	0.20	0.19	0.10
H	0.49	0.33	0.22	0.09	0.20	0.42	0.39	0.00	0.59	0.24	0.28	0.23	0.25	0.31	0.39	0.23	0.23	0.21	0.25	0.30
G	0.09	0.73	0.50	0.68	0.77	1.00	0.20	0.59	0.00	0.53	0.36	0.50	0.38	0.29	0.20	0.42	0.39	0.39	0.38	0.29
K	0.47	0.20	0.05	0.27	0.33	0.59	0.35	0.24	0.53	0.00	0.39	0.04	0.16	0.27	0.35	0.12	0.17	0.19	0.16	0.28
P	0.27	0.56	0.35	0.38	0.48	0.66	0.23	0.28	0.36	0.39	0.00	0.35	0.26	0.19	0.21	0.28	0.24	0.21	0.26	0.17
E	0.43	0.24	0.01	0.27	0.34	0.60	0.31	0.23	0.50	0.04	0.35	0.00	0.12	0.23	0.31	0.08	0.12	0.15	0.12	0.24
D	0.31	0.35	0.12	0.33	0.42	0.67	0.19	0.25	0.38	0.16	0.26	0.12	0.00	0.12	0.19	0.04	0.03	0.07	0.01	0.13
T	0.20	0.47	0.23	0.39	0.49	0.72	0.09	0.31	0.29	0.27	0.19	0.23	0.12	0.00	0.09	0.15	0.11	0.11	0.11	0.02
C	0.12	0.55	0.31	0.48	0.58	0.80	0.01	0.39	0.20	0.35	0.21	0.31	0.19	0.09	0.00	0.23	0.19	0.19	0.19	0.09
M	0.35	0.32	0.08	0.30	0.39	0.64	0.23	0.23	0.42	0.12	0.28	0.08	0.04	0.15	0.23	0.00	0.05	0.08	0.04	0.16
L	0.31	0.36	0.12	0.31	0.40	0.64	0.19	0.23	0.39	0.17	0.24	0.12	0.03	0.11	0.19	0.05	0.00	0.04	0.03	0.12
I	0.31	0.37	0.14	0.29	0.39	0.63	0.20	0.21	0.39	0.19	0.21	0.15	0.07	0.11	0.19	0.08	0.04	0.00	0.06	0.11
N	0.31	0.36	0.12	0.33	0.41	0.66	0.19	0.25	0.38	0.16	0.26	0.12	0.01	0.11	0.19	0.04	0.03	0.06	0.00	0.12
V	0.20	0.47	0.24	0.39	0.49	0.71	0.10	0.30	0.29	0.28	0.17	0.24	0.13	0.02	0.09	0.16	0.12	0.11	0.12	0.00

Supporting table S19. Scaled distance matrix MSWHIM using 2 PCs.

Residue	A	R	Q	F	Y	W	S	H	G	K	P	E	D	T	C	M	L	I	N	V
A	0.00	0.24	0.60	0.75	0.81	0.87	0.19	0.75	0.29	0.12	0.28	0.52	0.67	0.31	0.04	0.37	0.24	0.30	0.40	0.30
R	0.24	0.00	0.41	0.52	0.58	0.65	0.31	0.52	0.26	0.16	0.23	0.37	0.60	0.31	0.20	0.40	0.32	0.41	0.17	0.43
Q	0.60	0.41	0.00	0.22	0.35	0.32	0.54	0.29	0.65	0.56	0.36	0.64	0.93	0.41	0.56	0.46	0.50	0.56	0.38	0.61
F	0.75	0.52	0.22	0.00	0.13	0.13	0.73	0.09	0.72	0.68	0.55	0.62	0.90	0.62	0.71	0.68	0.69	0.77	0.41	0.81
Y	0.81	0.58	0.35	0.13	0.00	0.15	0.82	0.06	0.73	0.73	0.65	0.59	0.86	0.72	0.77	0.79	0.79	0.87	0.44	0.91
W	0.87	0.65	0.32	0.13	0.15	0.00	0.85	0.16	0.84	0.81	0.67	0.72	1.00	0.73	0.83	0.78	0.81	0.88	0.53	0.93
S	0.19	0.31	0.54	0.73	0.82	0.85	0.00	0.76	0.47	0.28	0.18	0.66	0.85	0.15	0.17	0.19	0.06	0.11	0.47	0.12
H	0.75	0.52	0.29	0.09	0.06	0.16	0.76	0.00	0.69	0.68	0.59	0.56	0.84	0.66	0.72	0.73	0.73	0.81	0.39	0.85
G	0.29	0.26	0.65	0.72	0.73	0.84	0.47	0.69	0.00	0.19	0.47	0.26	0.38	0.54	0.30	0.62	0.50	0.58	0.30	0.59
K	0.12	0.16	0.56	0.68	0.73	0.81	0.28	0.68	0.19	0.00	0.30	0.41	0.57	0.36	0.11	0.43	0.31	0.39	0.30	0.40
P	0.28	0.23	0.36	0.55	0.65	0.67	0.18	0.59	0.47	0.30	0.00	0.60	0.83	0.09	0.24	0.18	0.14	0.23	0.36	0.26
E	0.52	0.37	0.64	0.62	0.59	0.72	0.66	0.56	0.26	0.41	0.60	0.00	0.29	0.68	0.51	0.77	0.68	0.77	0.28	0.79
D	0.67	0.60	0.93	0.90	0.86	1.00	0.85	0.84	0.38	0.57	0.83	0.29	0.00	0.91	0.68	0.99	0.88	0.97	0.55	0.97
T	0.31	0.31	0.41	0.62	0.72	0.73	0.15	0.66	0.54	0.36	0.09	0.68	0.91	0.00	0.27	0.09	0.09	0.15	0.45	0.20
C	0.04	0.20	0.56	0.71	0.77	0.83	0.17	0.72	0.30	0.11	0.24	0.51	0.68	0.27	0.00	0.34	0.22	0.29	0.37	0.29
M	0.37	0.40	0.46	0.68	0.79	0.78	0.19	0.73	0.62	0.43	0.18	0.77	0.99	0.09	0.34	0.00	0.13	0.12	0.53	0.17
L	0.24	0.32	0.50	0.69	0.79	0.81	0.06	0.73	0.50	0.31	0.14	0.68	0.88	0.09	0.22	0.13	0.00	0.09	0.47	0.12
I	0.30	0.41	0.56	0.77	0.87	0.88	0.11	0.81	0.58	0.39	0.23	0.77	0.97	0.15	0.29	0.12	0.09	0.00	0.56	0.05
N	0.40	0.17	0.38	0.41	0.44	0.53	0.47	0.39	0.30	0.30	0.36	0.28	0.55	0.45	0.37	0.53	0.47	0.56	0.00	0.59
V	0.30	0.43	0.61	0.81	0.91	0.93	0.12	0.85	0.59	0.40	0.26	0.79	0.97	0.20	0.29	0.17	0.12	0.05	0.59	0.00

Supporting table S20. Scaled distance matrix FASGAI using 2 PCs.

Residue	A	R	Q	F	Y	W	S	H	G	K	P	E	D	T	C	M	L	I	N	V
A	0.00	0.40	0.31	0.31	0.44	0.29	0.48	0.16	0.81	0.43	0.77	0.44	0.52	0.34	0.30	0.23	0.27	0.36	0.57	0.31
R	0.40	0.00	0.09	0.65	0.53	0.56	0.38	0.25	0.74	0.07	0.67	0.27	0.22	0.38	0.60	0.61	0.67	0.73	0.35	0.68
Q	0.31	0.09	0.00	0.56	0.46	0.47	0.34	0.16	0.71	0.14	0.65	0.29	0.24	0.31	0.51	0.53	0.58	0.64	0.35	0.59
F	0.31	0.65	0.56	0.00	0.39	0.14	0.55	0.40	0.77	0.70	0.76	0.74	0.70	0.39	0.10	0.29	0.22	0.10	0.68	0.07
Y	0.44	0.53	0.46	0.39	0.00	0.25	0.22	0.38	0.39	0.59	0.37	0.74	0.44	0.14	0.29	0.60	0.57	0.49	0.35	0.46
W	0.29	0.56	0.47	0.14	0.25	0.00	0.41	0.33	0.63	0.61	0.62	0.70	0.57	0.25	0.05	0.38	0.33	0.24	0.54	0.21
S	0.48	0.38	0.34	0.55	0.22	0.41	0.00	0.35	0.37	0.44	0.32	0.63	0.24	0.16	0.46	0.68	0.69	0.65	0.14	0.61
H	0.16	0.25	0.16	0.40	0.38	0.33	0.35	0.00	0.71	0.30	0.66	0.38	0.36	0.25	0.35	0.38	0.43	0.47	0.42	0.42
G	0.81	0.74	0.71	0.77	0.39	0.63	0.37	0.71	0.00	0.80	0.07	1.00	0.55	0.47	0.67	0.99	0.96	0.86	0.40	0.84
K	0.43	0.07	0.14	0.70	0.59	0.61	0.44	0.30	0.80	0.00	0.74	0.22	0.27	0.45	0.65	0.63	0.70	0.77	0.41	0.72
P	0.77	0.67	0.65	0.76	0.37	0.62	0.32	0.66	0.07	0.74	0.00	0.94	0.48	0.43	0.66	0.96	0.94	0.85	0.33	0.82
E	0.44	0.27	0.29	0.74	0.74	0.70	0.63	0.38	1.00	0.22	0.94	0.00	0.48	0.60	0.72	0.59	0.68	0.79	0.62	0.74
D	0.52	0.22	0.24	0.70	0.44	0.57	0.24	0.36	0.55	0.27	0.48	0.48	0.00	0.33	0.62	0.75	0.78	0.79	0.15	0.74
T	0.34	0.38	0.31	0.39	0.14	0.25	0.16	0.25	0.47	0.45	0.43	0.60	0.33	0.00	0.30	0.53	0.53	0.49	0.29	0.45
C	0.30	0.60	0.51	0.10	0.29	0.05	0.46	0.35	0.67	0.65	0.66	0.72	0.62	0.30	0.00	0.35	0.30	0.19	0.59	0.16
M	0.23	0.61	0.53	0.29	0.60	0.38	0.68	0.38	0.99	0.63	0.96	0.59	0.75	0.53	0.35	0.00	0.10	0.27	0.78	0.24
L	0.27	0.67	0.58	0.22	0.57	0.33	0.69	0.43	0.96	0.70	0.94	0.68	0.78	0.53	0.30	0.10	0.00	0.18	0.80	0.16
I	0.36	0.73	0.64	0.10	0.49	0.24	0.65	0.47	0.86	0.77	0.85	0.79	0.79	0.49	0.19	0.27	0.18	0.00	0.77	0.05
N	0.57	0.35	0.35	0.68	0.35	0.54	0.14	0.42	0.40	0.41	0.33	0.62	0.15	0.29	0.59	0.78	0.80	0.77	0.00	0.73
V	0.31	0.68	0.59	0.07	0.46	0.21	0.61	0.42	0.84	0.72	0.82	0.74	0.74	0.45	0.16	0.24	0.16	0.05	0.73	0.00

Supporting table S21. Scaled distance matrix BLOSUM using 2 PCs.

Residue	A	R	Q	F	Y	W	S	H	G	K	P	E	D	T	C	M	L	I	N	V
A	0.00	0.37	0.41	0.66	0.76	0.93	0.23	0.66	0.35	0.36	0.22	0.47	0.45	0.06	0.30	0.28	0.38	0.40	0.47	0.32
R	0.37	0.00	0.03	0.71	0.69	0.86	0.17	0.36	0.04	0.07	0.23	0.12	0.15	0.32	0.64	0.55	0.68	0.72	0.13	0.66
Q	0.41	0.03	0.00	0.73	0.69	0.86	0.20	0.34	0.07	0.09	0.26	0.10	0.14	0.35	0.67	0.58	0.71	0.76	0.11	0.70
F	0.66	0.71	0.73	0.00	0.23	0.33	0.74	0.62	0.67	0.76	0.78	0.83	0.86	0.65	0.56	0.45	0.48	0.55	0.83	0.59
Y	0.76	0.69	0.69	0.23	0.00	0.18	0.76	0.48	0.65	0.75	0.81	0.78	0.83	0.73	0.74	0.60	0.67	0.75	0.79	0.77
W	0.93	0.86	0.86	0.33	0.18	0.00	0.94	0.62	0.83	0.92	0.99	0.95	1.00	0.90	0.89	0.76	0.81	0.88	0.96	0.91
S	0.23	0.17	0.20	0.74	0.76	0.94	0.00	0.52	0.16	0.13	0.06	0.24	0.22	0.18	0.53	0.47	0.59	0.62	0.24	0.55
H	0.66	0.36	0.34	0.62	0.48	0.62	0.52	0.00	0.36	0.42	0.58	0.39	0.45	0.61	0.83	0.70	0.83	0.89	0.40	0.86
G	0.35	0.04	0.07	0.67	0.65	0.83	0.16	0.36	0.00	0.09	0.22	0.16	0.19	0.29	0.60	0.51	0.64	0.69	0.17	0.63
K	0.36	0.07	0.09	0.76	0.75	0.92	0.13	0.42	0.09	0.00	0.18	0.11	0.11	0.30	0.64	0.56	0.69	0.73	0.12	0.66
P	0.22	0.23	0.26	0.78	0.81	0.99	0.06	0.58	0.22	0.18	0.00	0.28	0.25	0.18	0.52	0.48	0.59	0.62	0.28	0.54
E	0.47	0.12	0.10	0.83	0.78	0.95	0.24	0.39	0.16	0.11	0.28	0.00	0.06	0.42	0.75	0.66	0.80	0.84	0.01	0.77
D	0.45	0.15	0.14	0.86	0.83	1.00	0.22	0.45	0.19	0.11	0.25	0.06	0.00	0.40	0.74	0.67	0.80	0.83	0.06	0.76
T	0.06	0.32	0.35	0.65	0.73	0.90	0.18	0.61	0.29	0.30	0.18	0.42	0.40	0.00	0.34	0.30	0.41	0.44	0.42	0.36
C	0.30	0.64	0.67	0.56	0.74	0.89	0.53	0.83	0.60	0.64	0.52	0.75	0.74	0.34	0.00	0.14	0.11	0.10	0.75	0.03
M	0.28	0.55	0.58	0.45	0.60	0.76	0.47	0.70	0.51	0.56	0.48	0.66	0.67	0.30	0.14	0.00	0.13	0.19	0.67	0.17
L	0.38	0.68	0.71	0.48	0.67	0.81	0.59	0.83	0.64	0.69	0.59	0.80	0.80	0.41	0.11	0.13	0.00	0.07	0.80	0.12
I	0.40	0.72	0.76	0.55	0.75	0.88	0.62	0.89	0.69	0.73	0.62	0.84	0.83	0.44	0.10	0.19	0.07	0.00	0.84	0.09
N	0.47	0.13	0.11	0.83	0.79	0.96	0.24	0.40	0.17	0.12	0.28	0.01	0.06	0.42	0.75	0.67	0.80	0.84	0.00	0.78
V	0.32	0.66	0.70	0.59	0.77	0.91	0.55	0.86	0.63	0.66	0.54	0.77	0.76	0.36	0.03	0.17	0.12	0.09	0.78	0.00

Supporting Table S22. The binned Z-Scales

Amino Acid	Lipophilicity (Z1)				Size (Z2)				Polarity (Z3)			Electronegativity (Z4)				Electrophilicity (Z5)			
	--	-	+	++	--	-	+	++	-	+	++	--	-	+	++	--	-	+	++
G	1	0	0	0	1	0	0	0	0	0	1	0	1	0	0	0	1	0	0
A	0	0	1	0	1	0	0	0	0	0	1	0	1	0	0	0	0	0	1
C	0	0	1	0	0	1	0	0	0	0	1	0	0	1	0	1	0	0	0
V	0	0	0	1	1	0	0	0	0	1	0	0	1	0	0	0	1	0	0
L	0	0	0	1	0	1	0	0	0	1	0	0	1	0	0	0	0	1	0
I	0	0	0	1	0	1	0	0	1	0	0	0	1	0	0	0	0	1	0
M	0	0	0	1	0	1	0	0	0	0	1	0	0	0	1	0	1	0	0
F	0	0	0	1	0	0	0	1	0	0	1	0	0	1	0	0	1	0	0
W	0	0	0	1	0	0	0	1	0	0	1	0	0	0	1	1	0	0	0
Y	0	0	0	1	0	0	0	1	0	0	1	0	1	0	0	1	0	0	0
H	1	0	0	0	0	0	0	1	0	0	1	0	0	0	1	0	0	1	0
T	0	0	1	0	1	0	0	0	0	1	0	1	0	0	0	0	1	0	0
P	0	1	0	0	0	0	1	0	0	0	1	0	0	1	0	0	0	0	1
S	1	0	0	0	0	1	0	0	0	0	1	1	0	0	0	0	0	1	0
D	1	0	0	0	0	0	1	0	0	0	1	1	0	0	0	0	0	1	0
N	1	0	0	0	0	0	1	0	0	0	1	0	1	0	0	0	0	0	1
E	1	0	0	0	0	0	1	0	0	1	0	1	0	0	0	0	1	0	0
Q	0	0	1	0	0	0	1	0	0	1	0	1	0	0	0	0	0	1	0
K	1	0	0	0	0	0	1	0	1	0	0	0	0	1	0	0	0	1	0
R	1	0	0	0	0	0	0	1	1	0	0	0	0	0	1	0	1	0	0
Average	0.40	0.05	0.20	0.35	0.20	0.25	0.30	0.25	0.15	0.25	0.60	0.25	0.35	0.20	0.20	0.15	0.35	0.35	0.15

The score on the scales is given as – (very low), - (low) , + (high) or ++ (very high). For the 3rd scale only 3 classes were used. Also note that scale 1 has been reversed to make it more intuitive (++ is now large instead of the other way around).

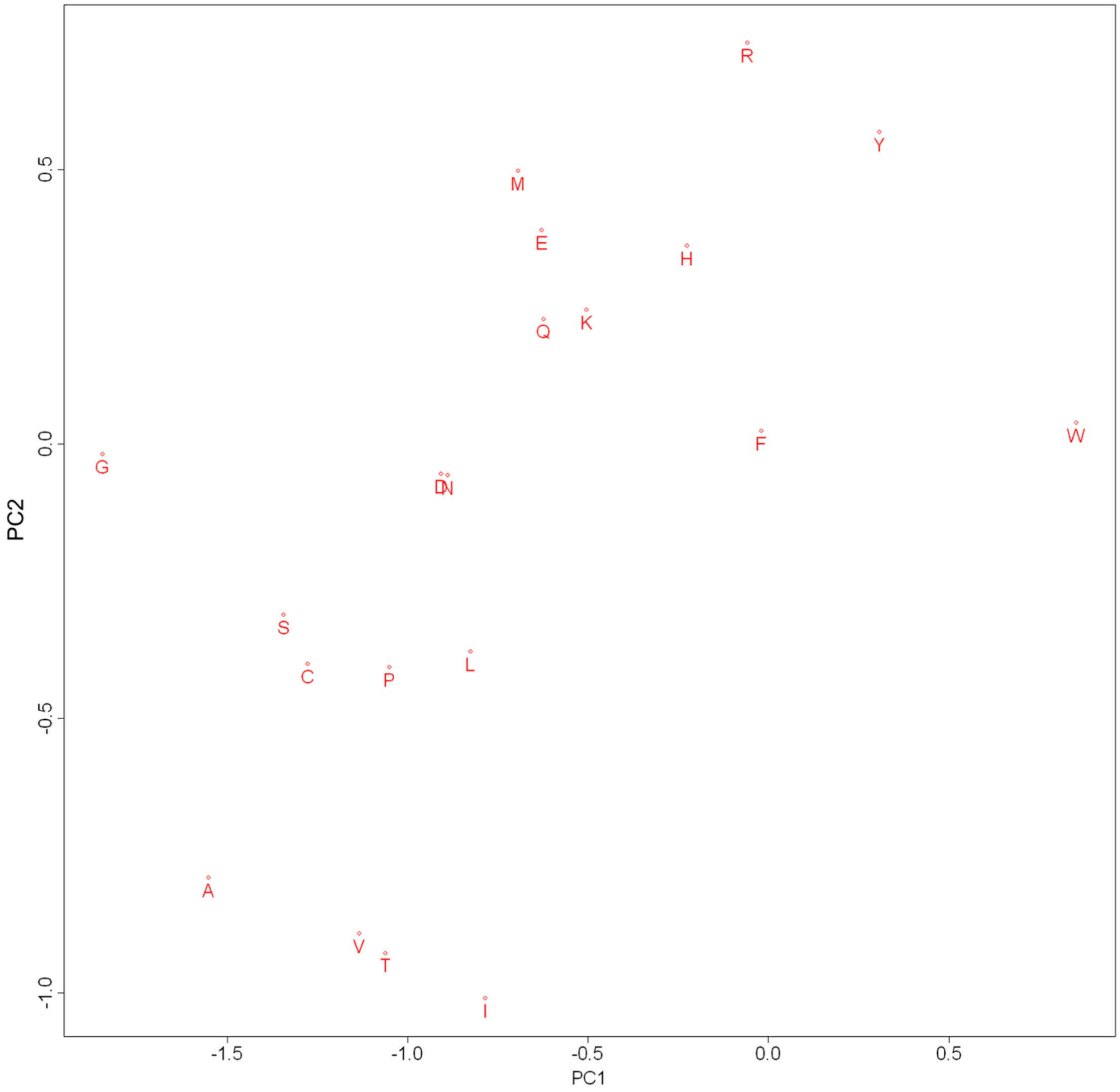

Supporting figure S1. Plot of the first two PCs in the ST-scales descriptor set.

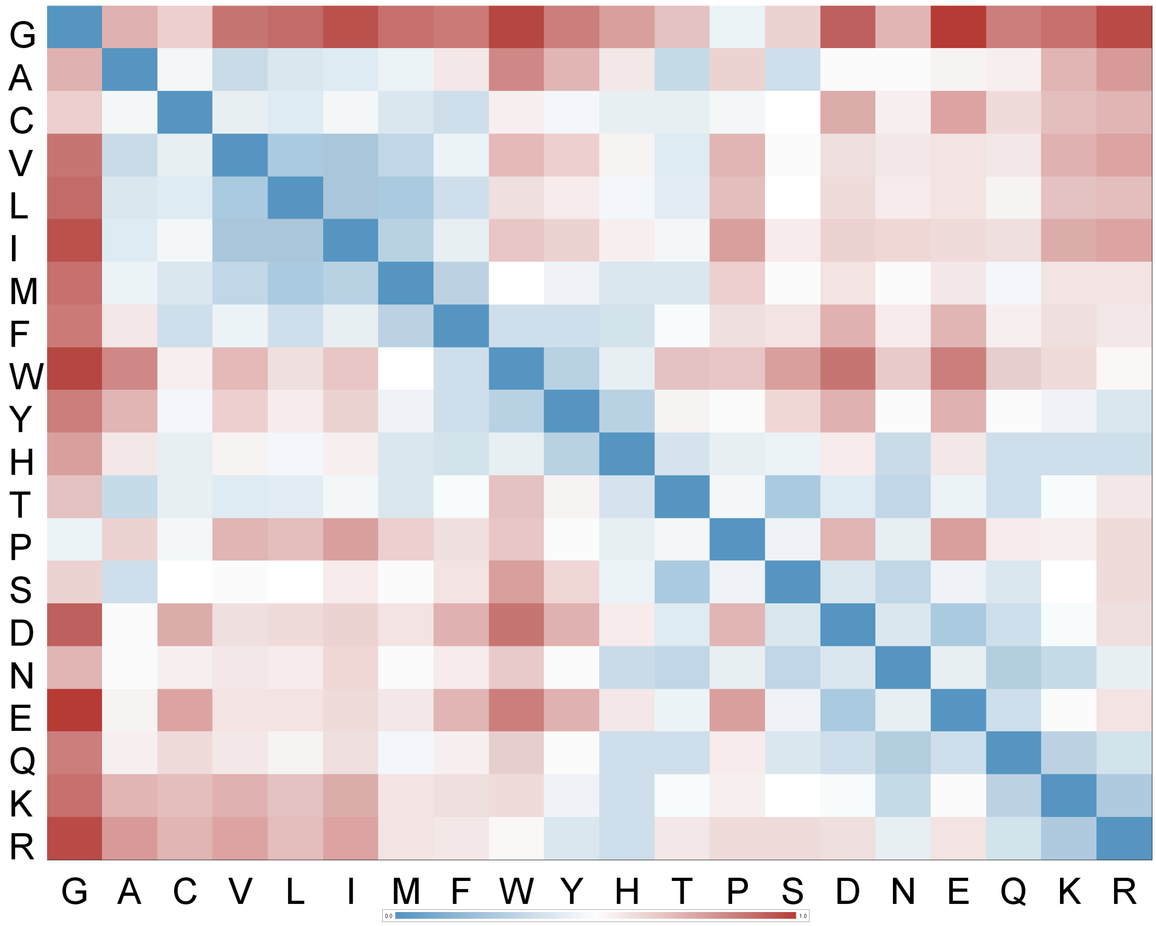

Supporting Figure S2. Heat map of the ProtFP_PCA (3) descriptor set characterizing the inter amino acids differences in.

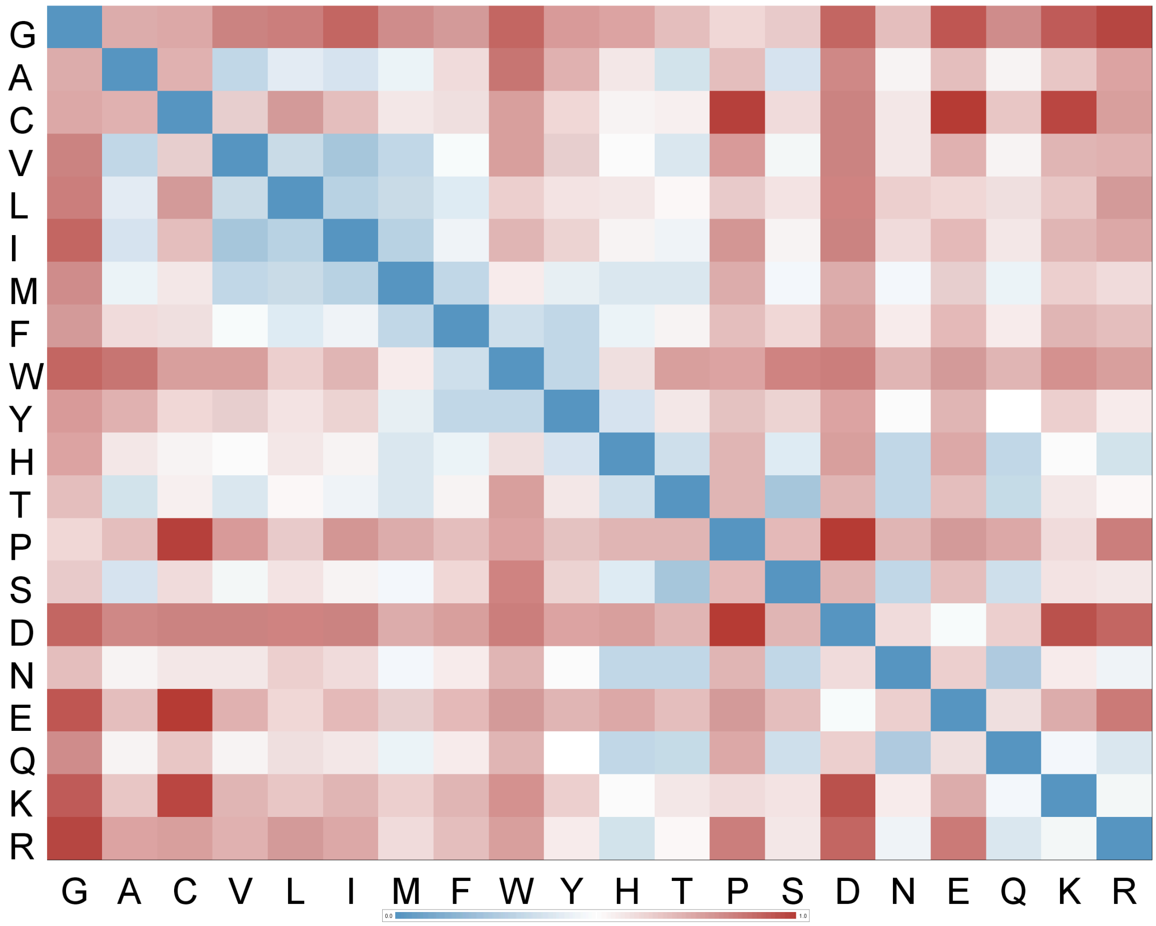

Supporting Figure S3. Heat map of the ProtFP_PCA (5) descriptor set characterizing the inter amino acids differences.

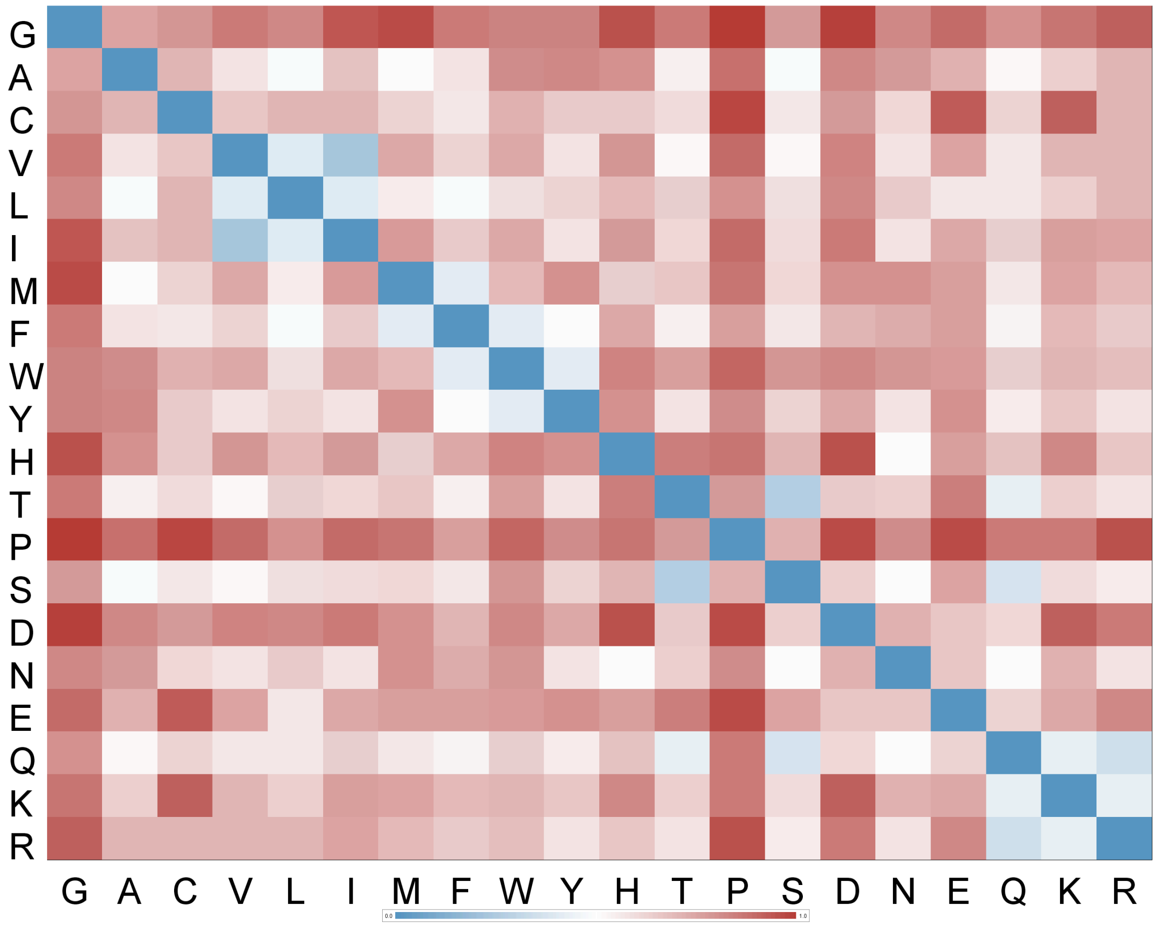

Supporting Figure S4. Heat map of the ProtFP_PCA (8) descriptor set characterizing the inter amino acids differences.

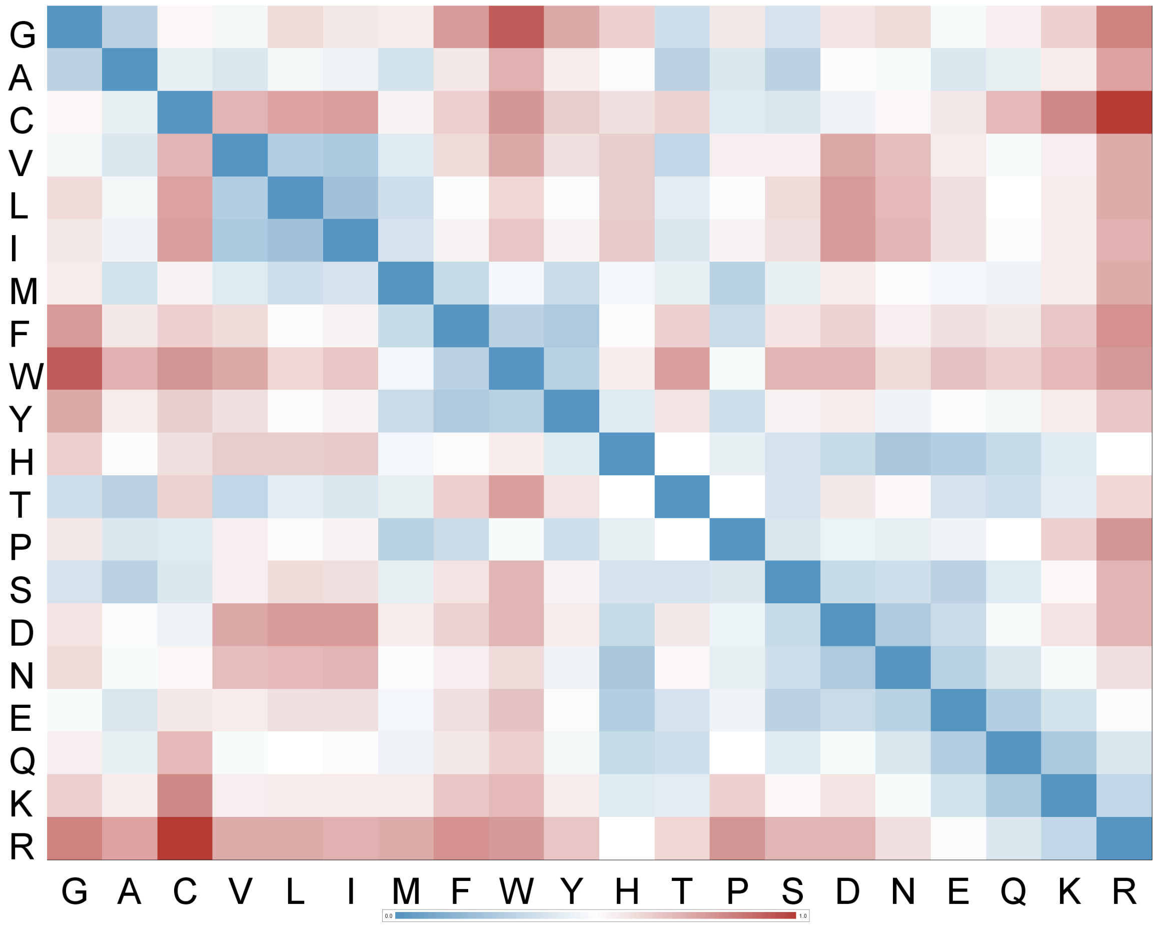

Supporting Figure S5. Heat map of the Z-Scales (3) descriptor set characterizing the inter amino acids differences.

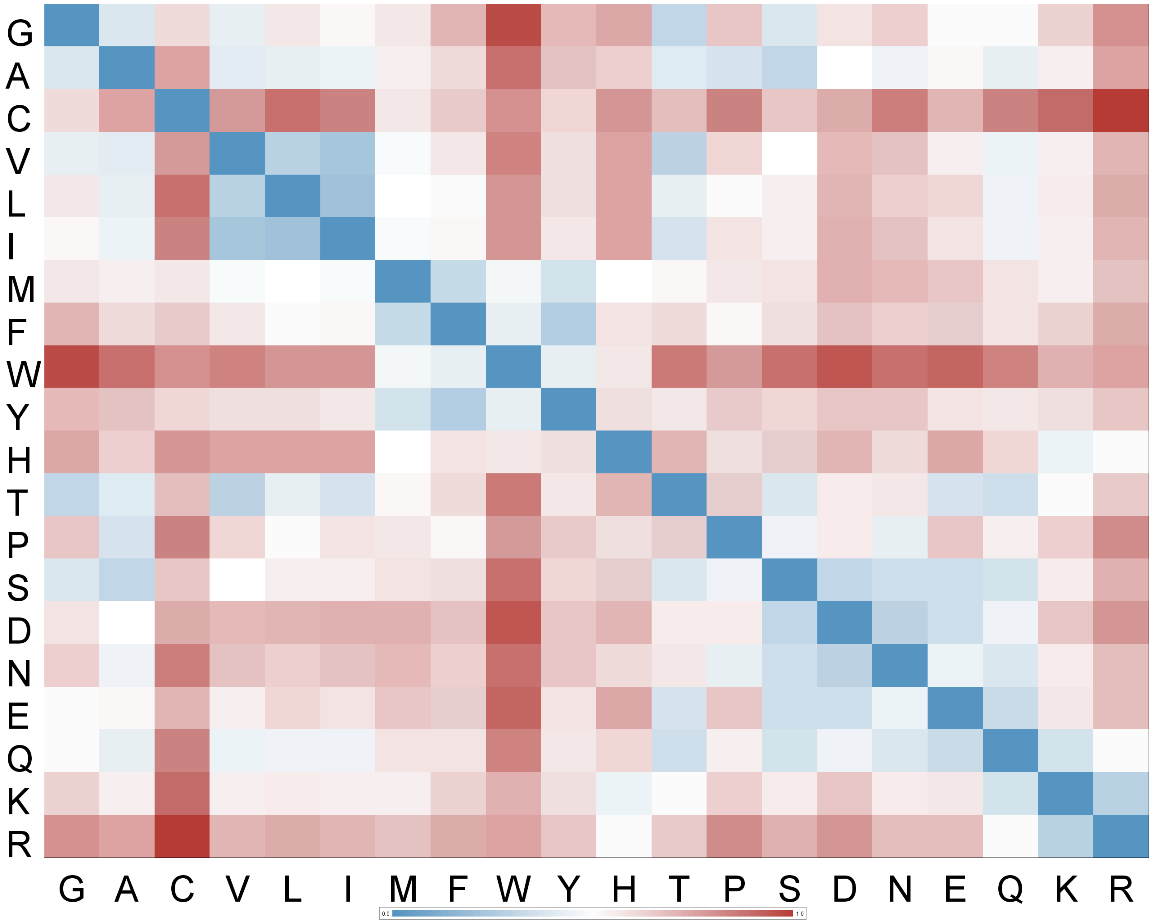

Supporting Figure S6. Heat map of the Z-Scales (5) descriptor set characterizing the inter amino acids differences.

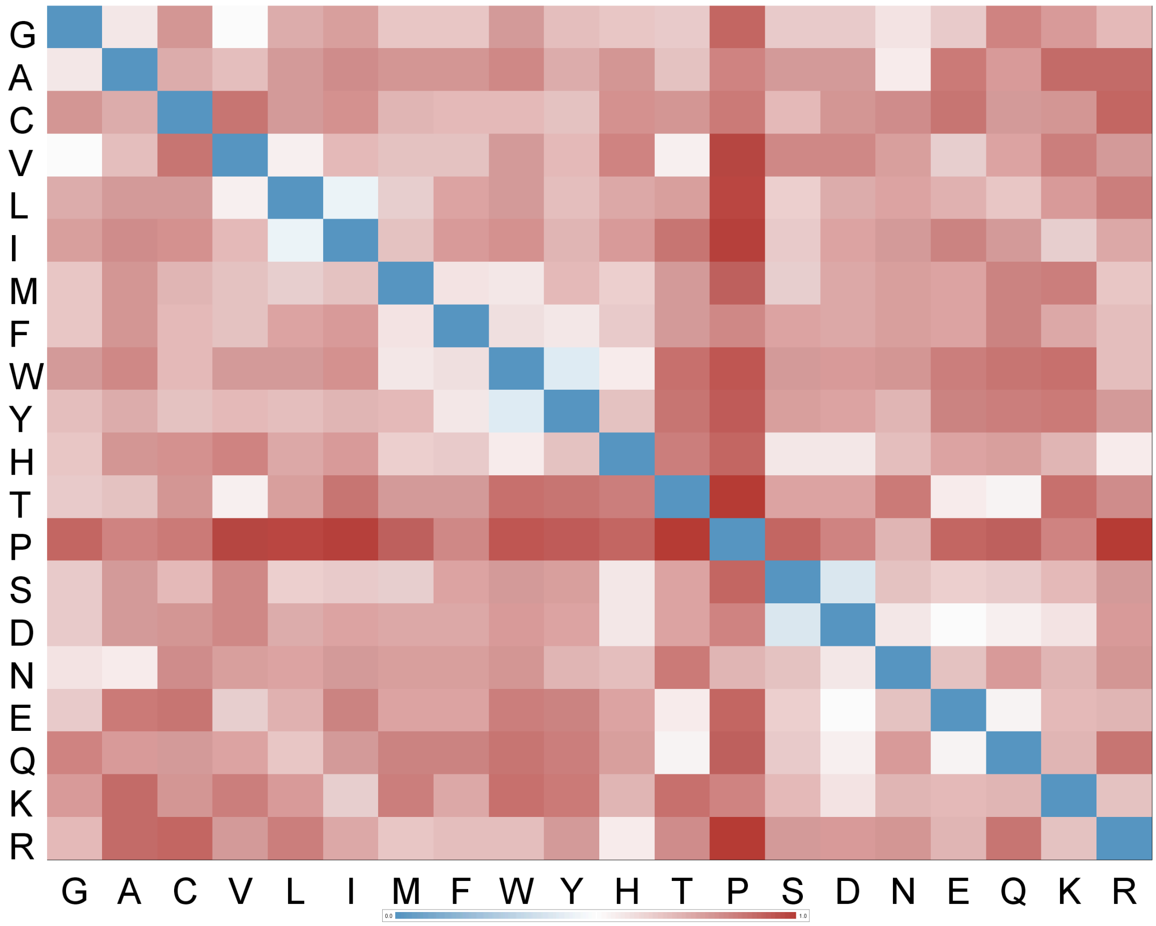

Supporting Figure S7. Heat map of the Z-Scales (Binned) descriptor set characterizing the inter amino acids differences.

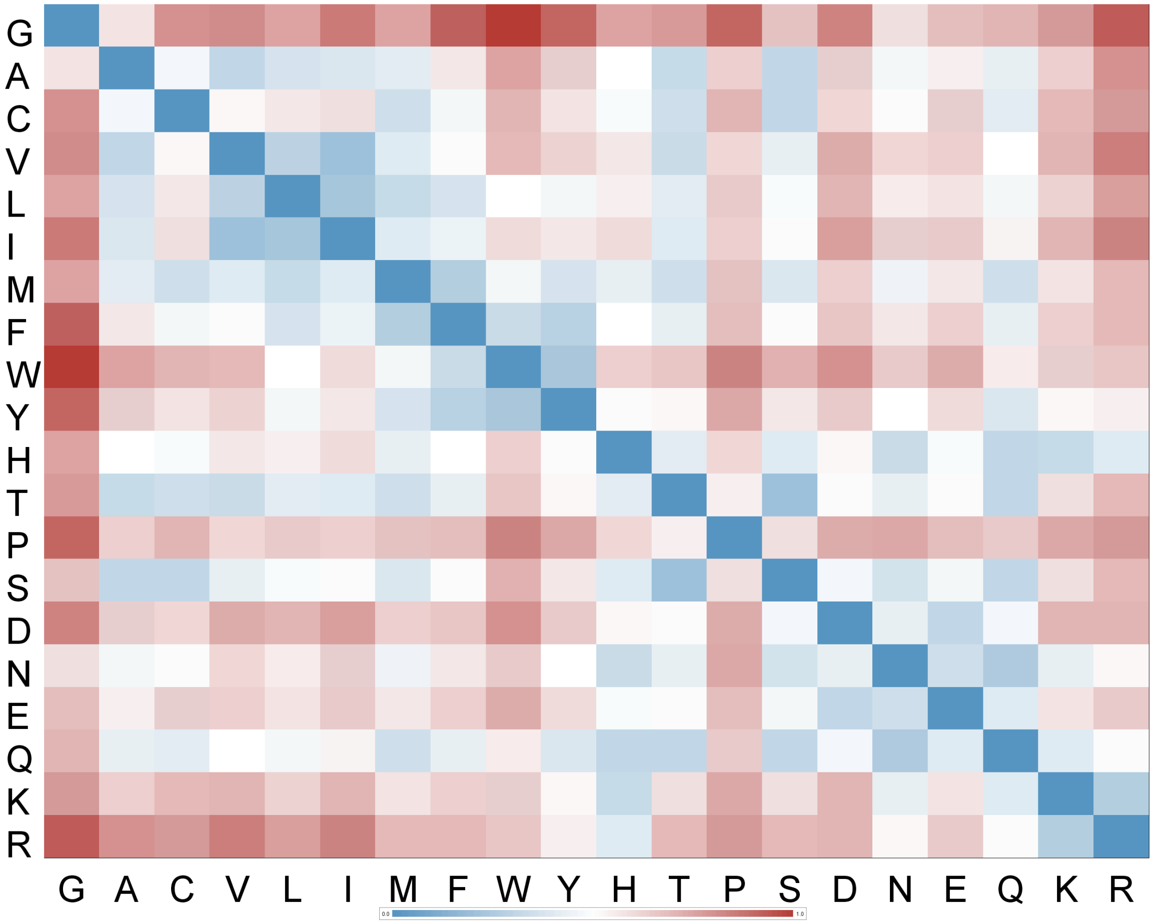

Supporting figure S8. Heat map of the VHSE descriptor set characterizing the inter amino acids differences.

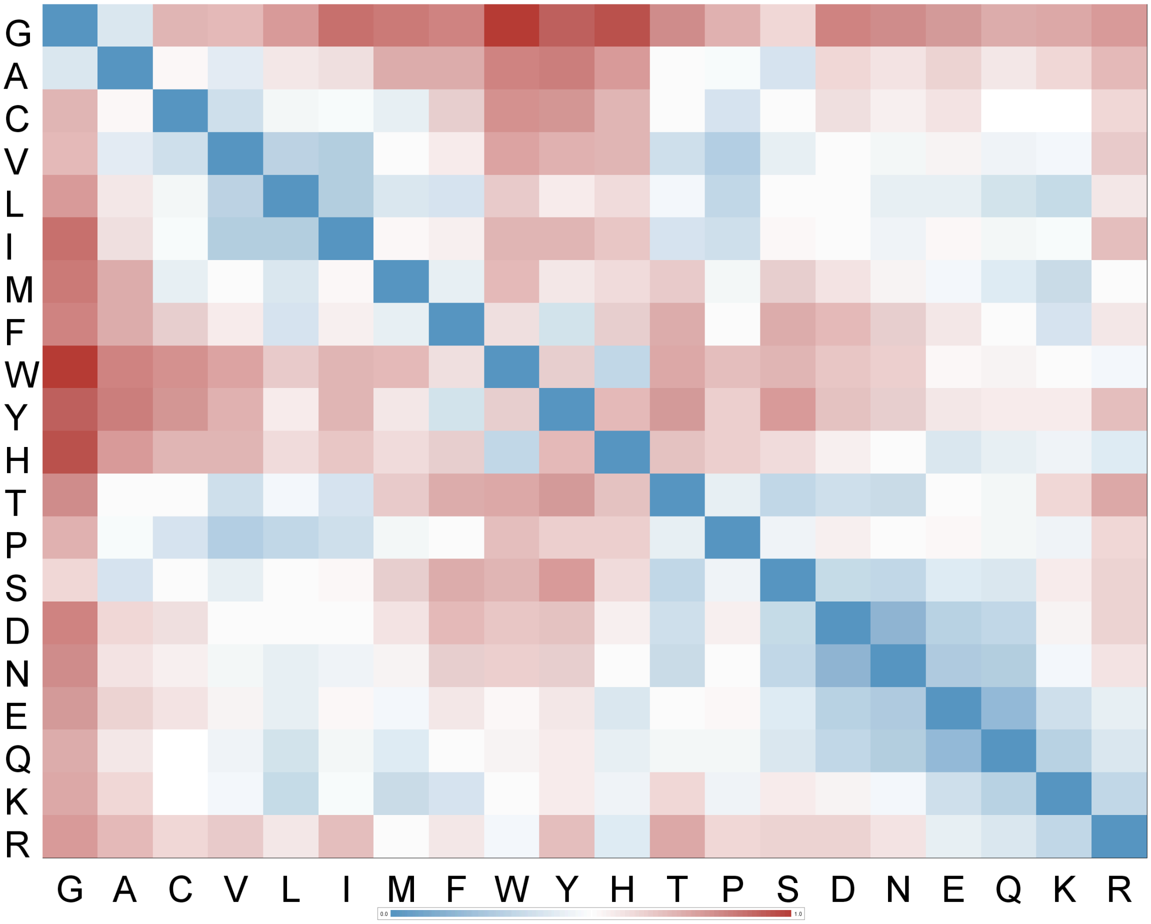

Supporting figure S9. Heat map of the ST-Scales descriptor set characterizing the inter amino acids differences.

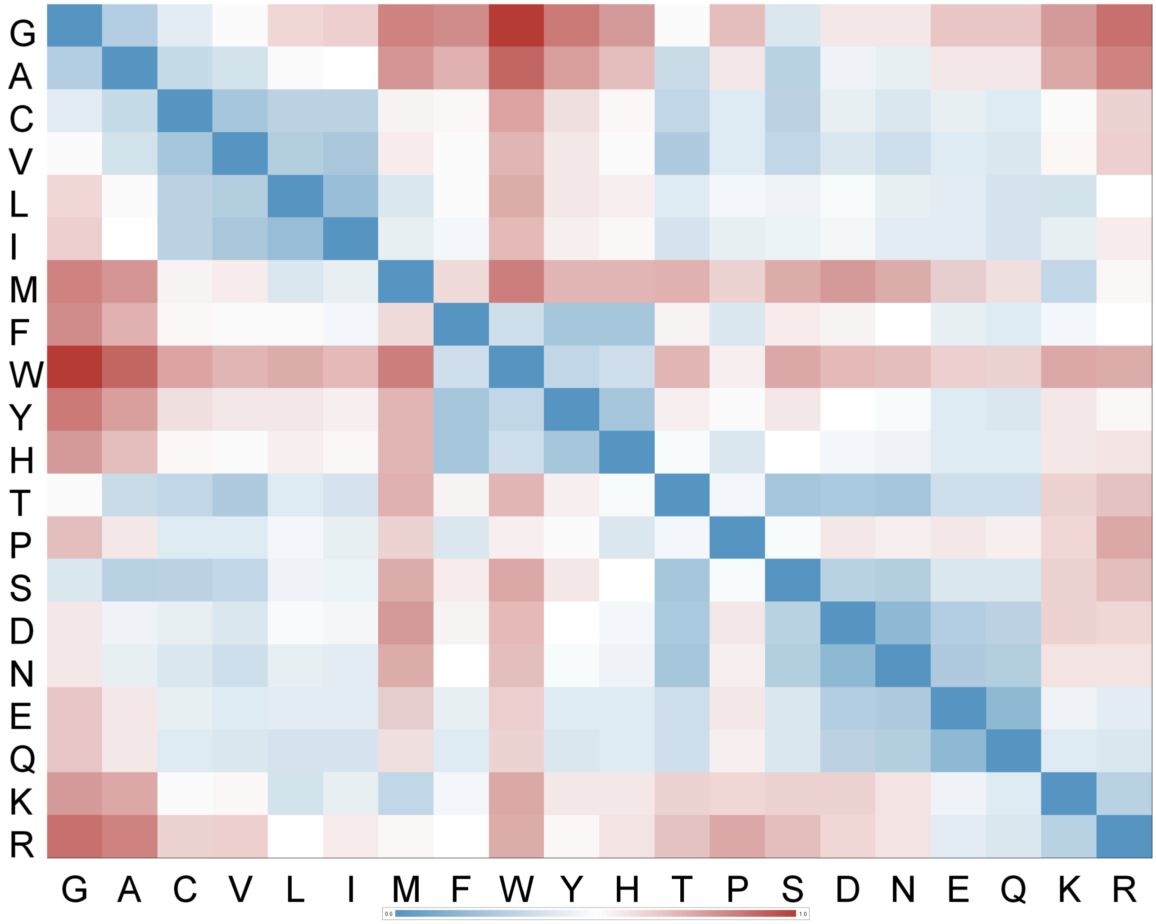

Supporting figure S10. Heat map of the T-Scales descriptor set characterizing the inter amino acids differences.

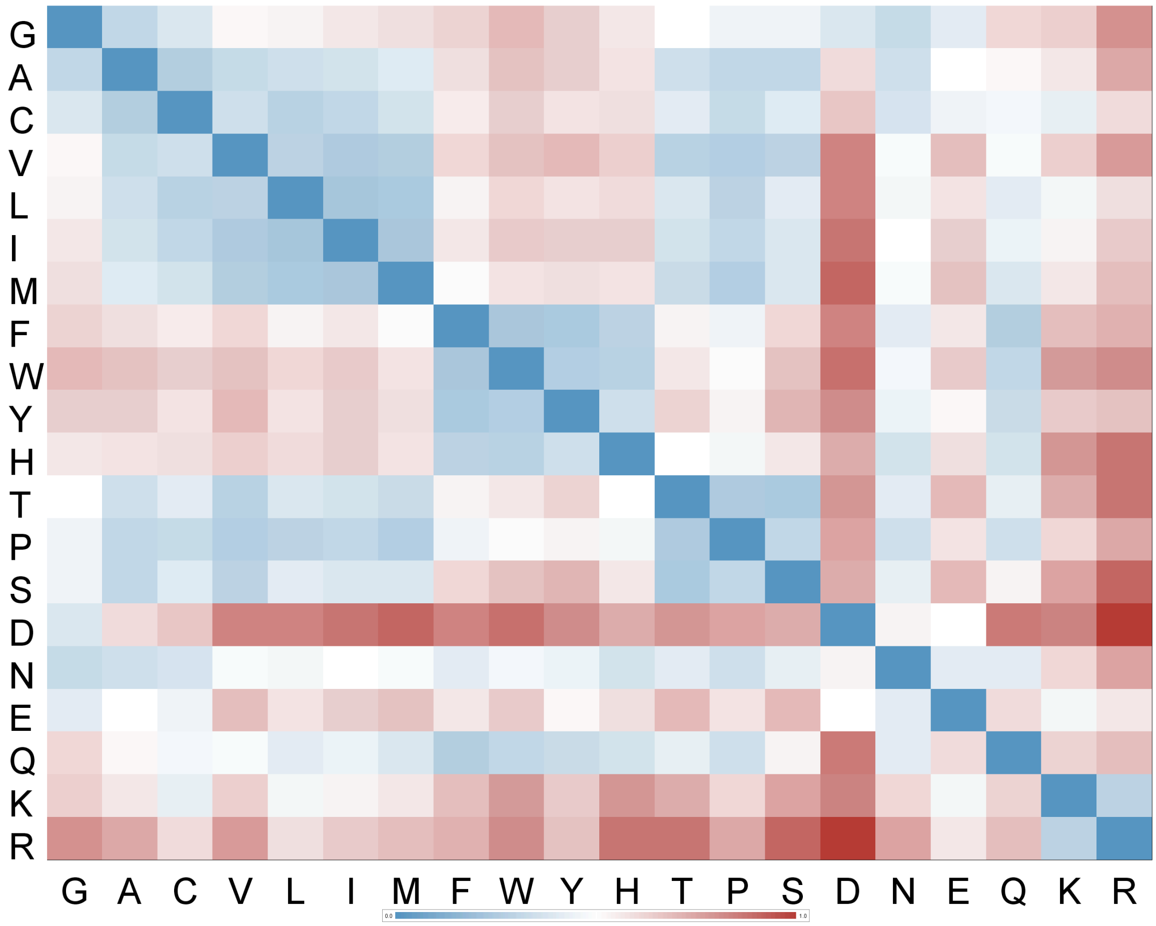

Supporting figure S11. Heat map of the MSWHIM descriptor set characterizing the inter amino acids differences.

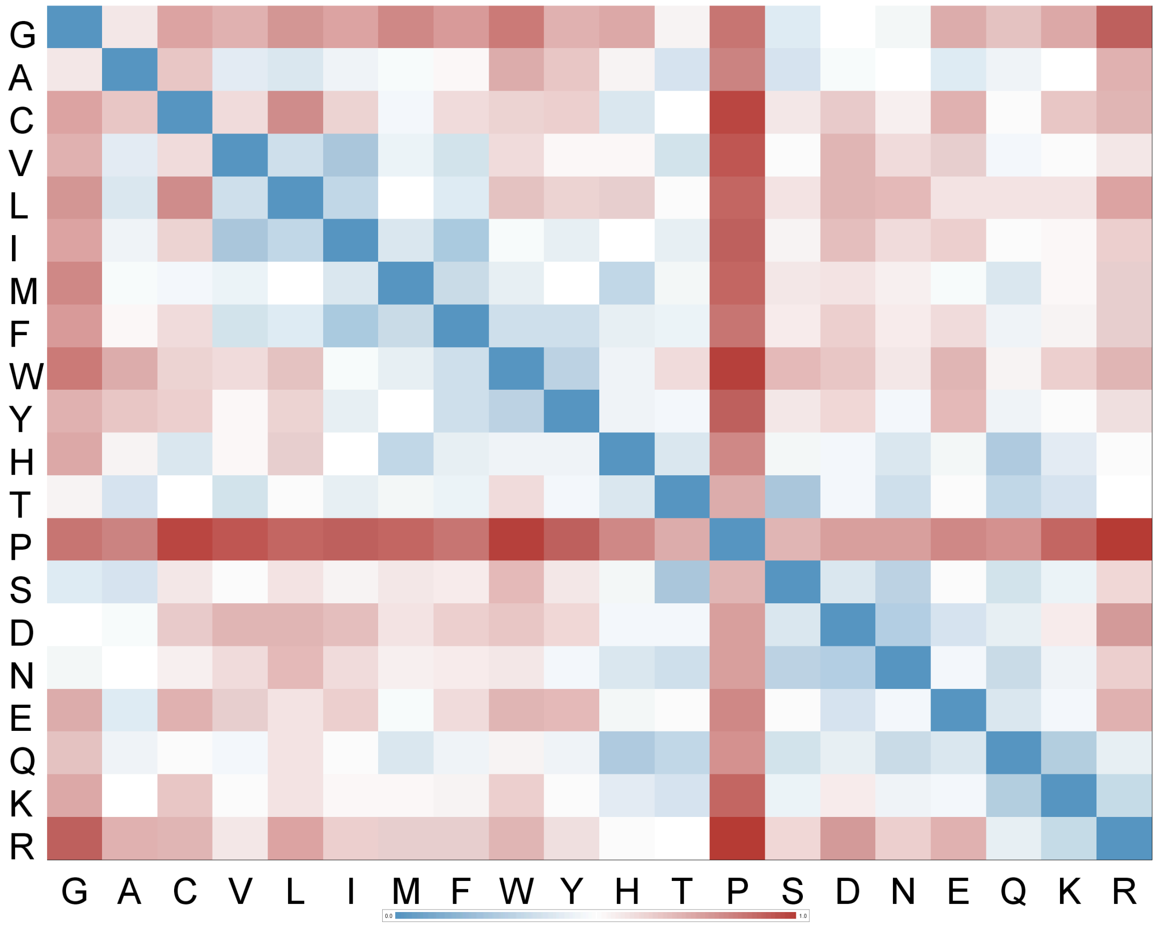

Supporting figure S12. Heat map of the FASGAI descriptor set characterizing the inter amino acids differences.

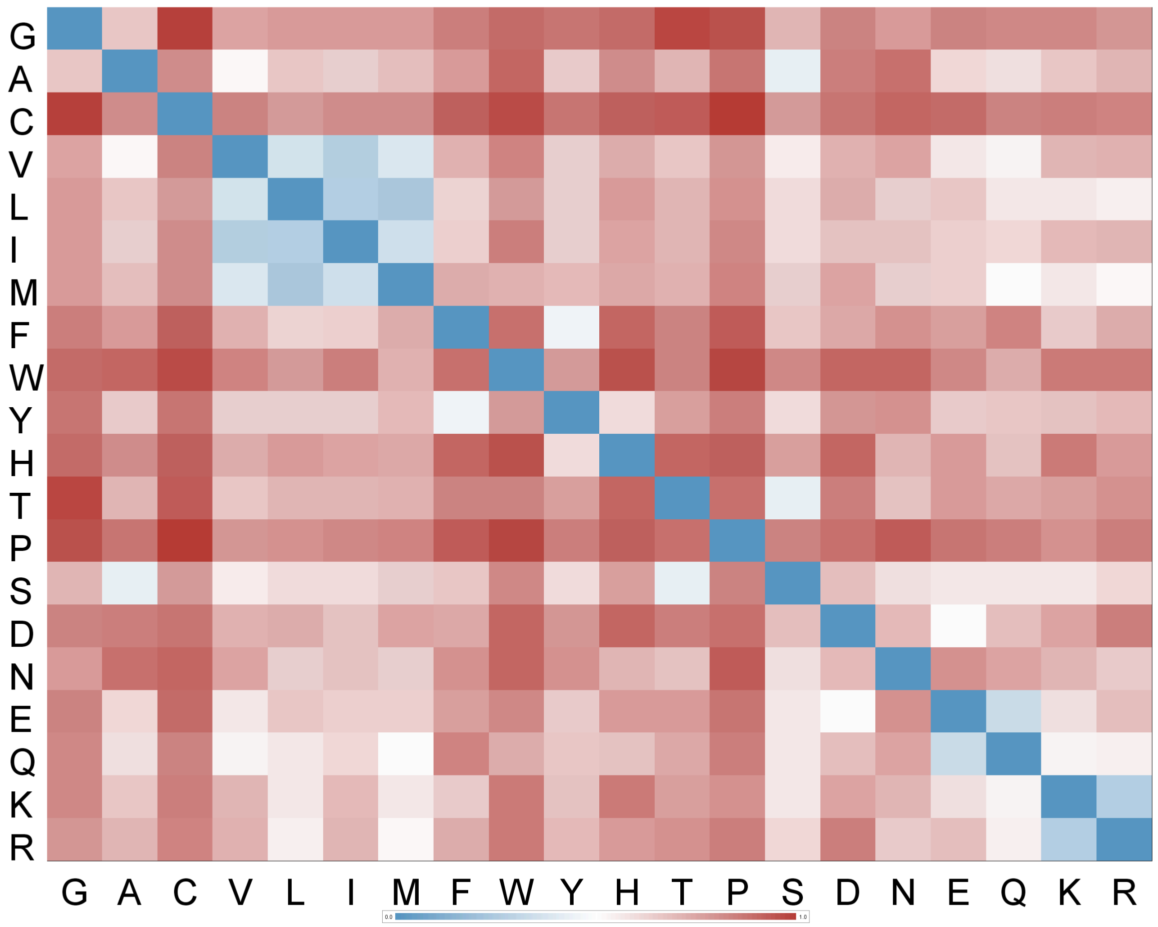

Supporting figure S13. Heat map of the BLOSUM descriptor set characterizing the inter amino acids differences.

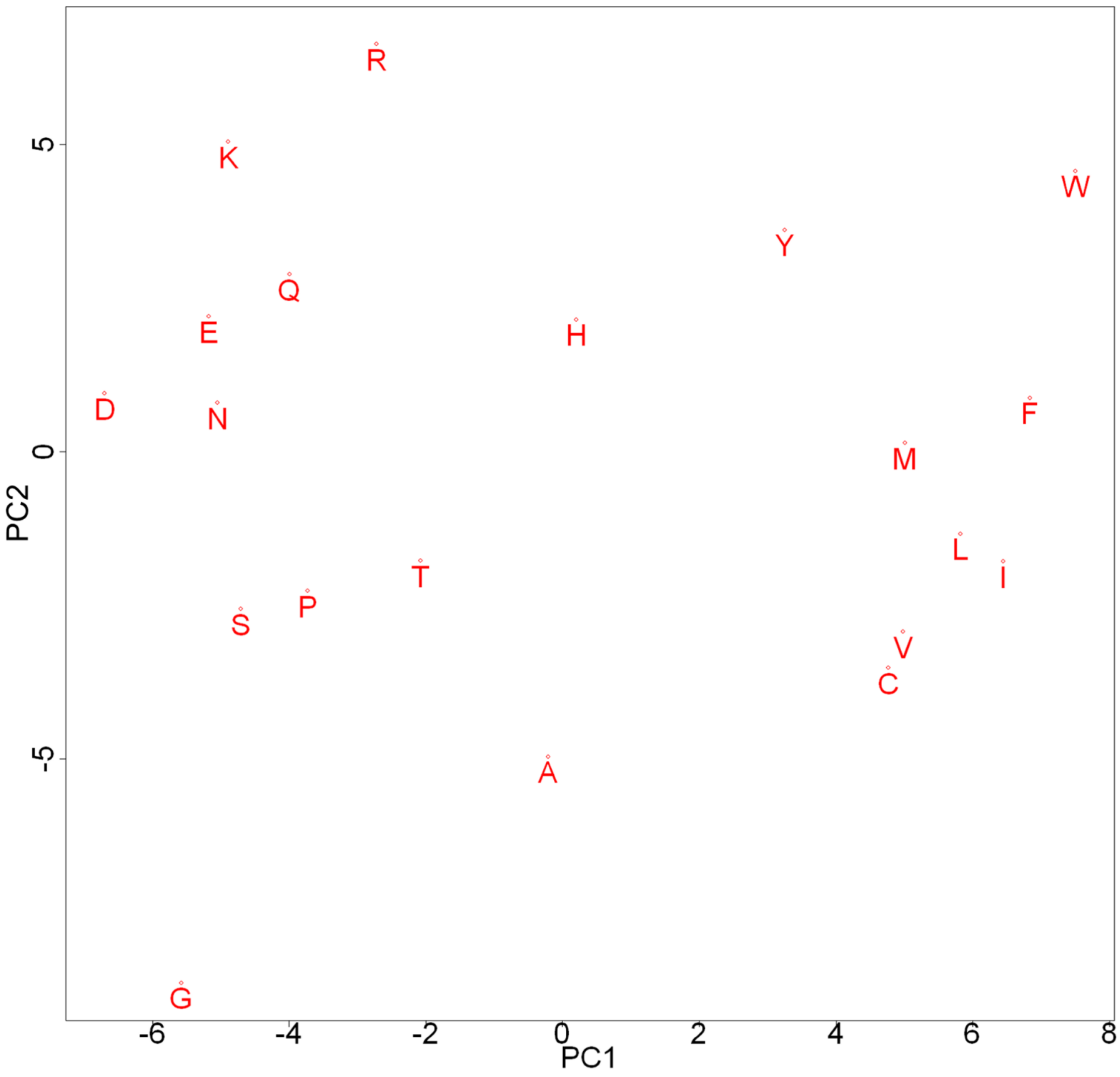

Supporting figure S14. Plot of the first two PCs in the ProtFP_PCA descriptor set.

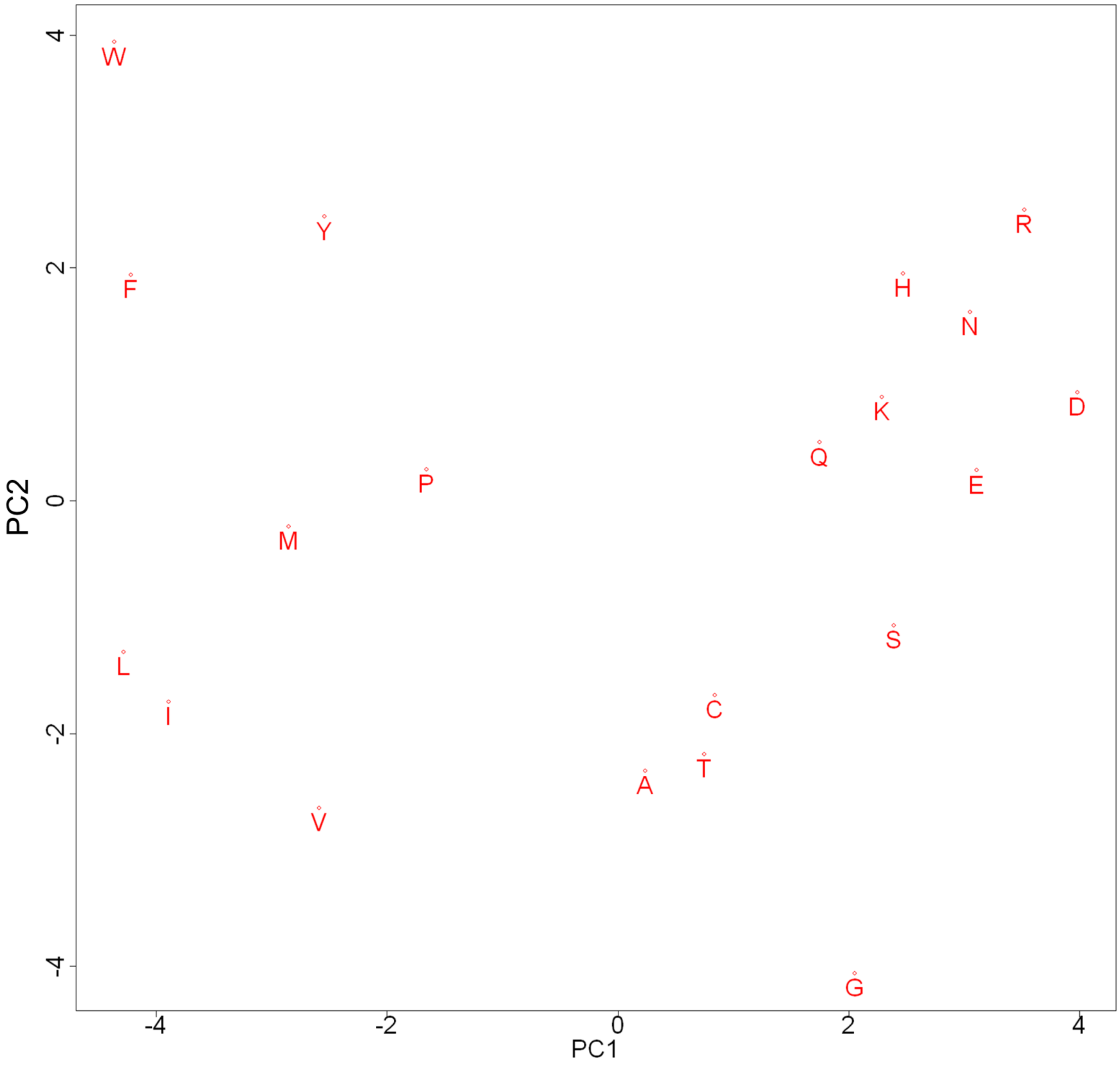

Supporting figure S15. Plot of the first two PCs in the Z-scales descriptor set.

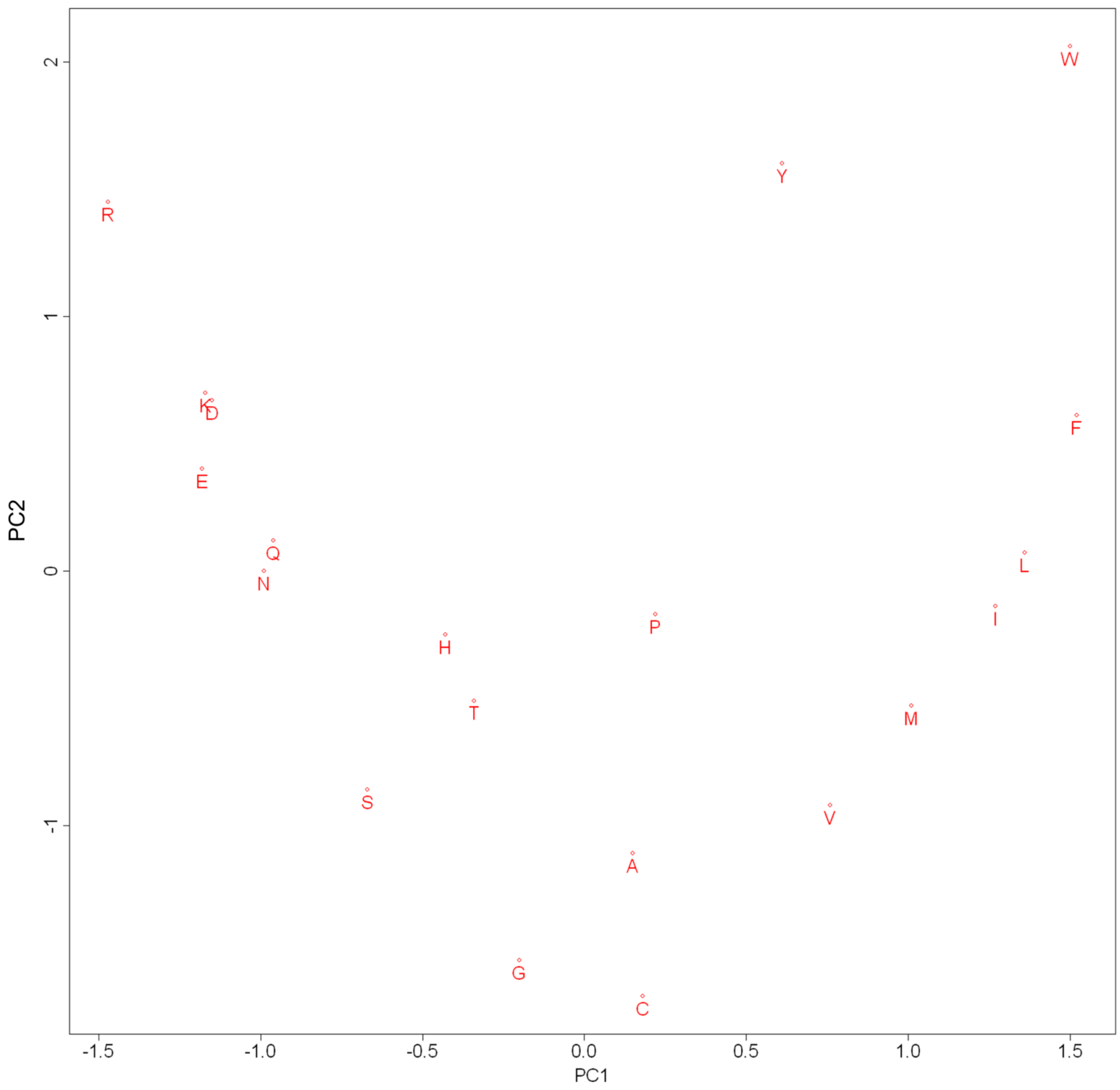

Supporting figure S16. Plot of the first two PCs in the VHSE descriptor set.

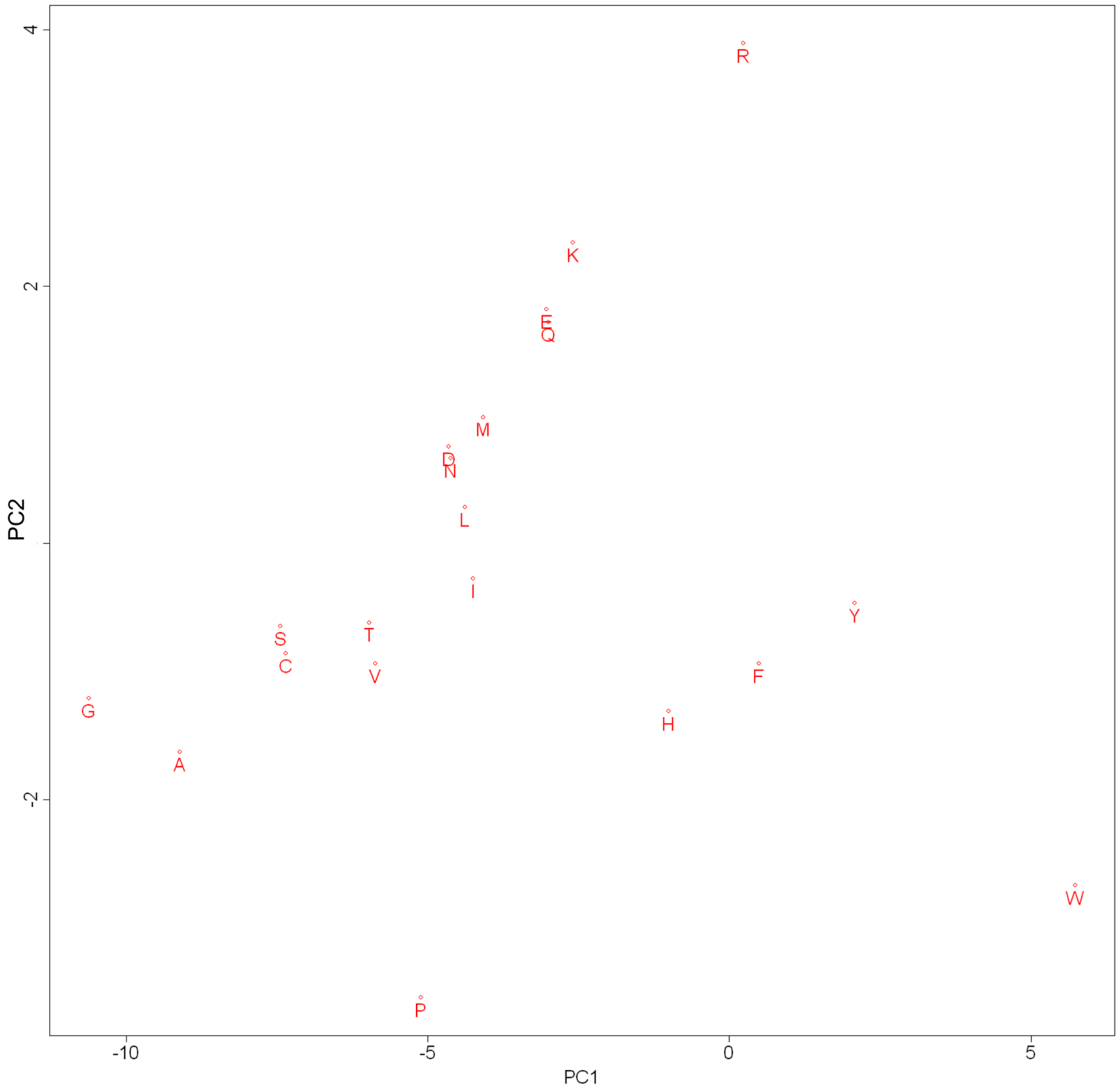

Supporting figure S17. Plot of the first two PCs in the T-scales descriptor set.

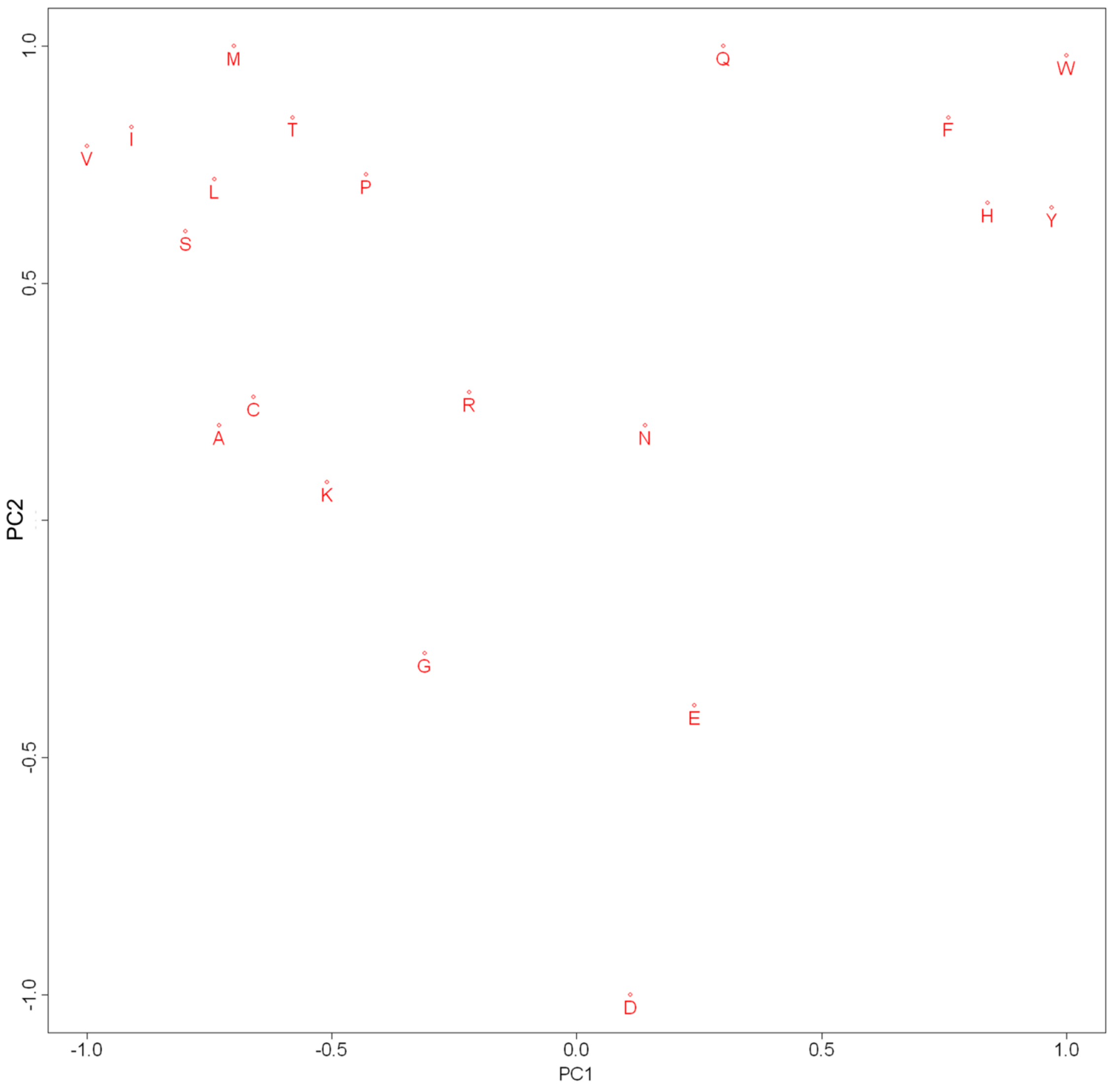

Supporting figure S18. Plot of the first two PCs in the MSWHIM descriptor set.

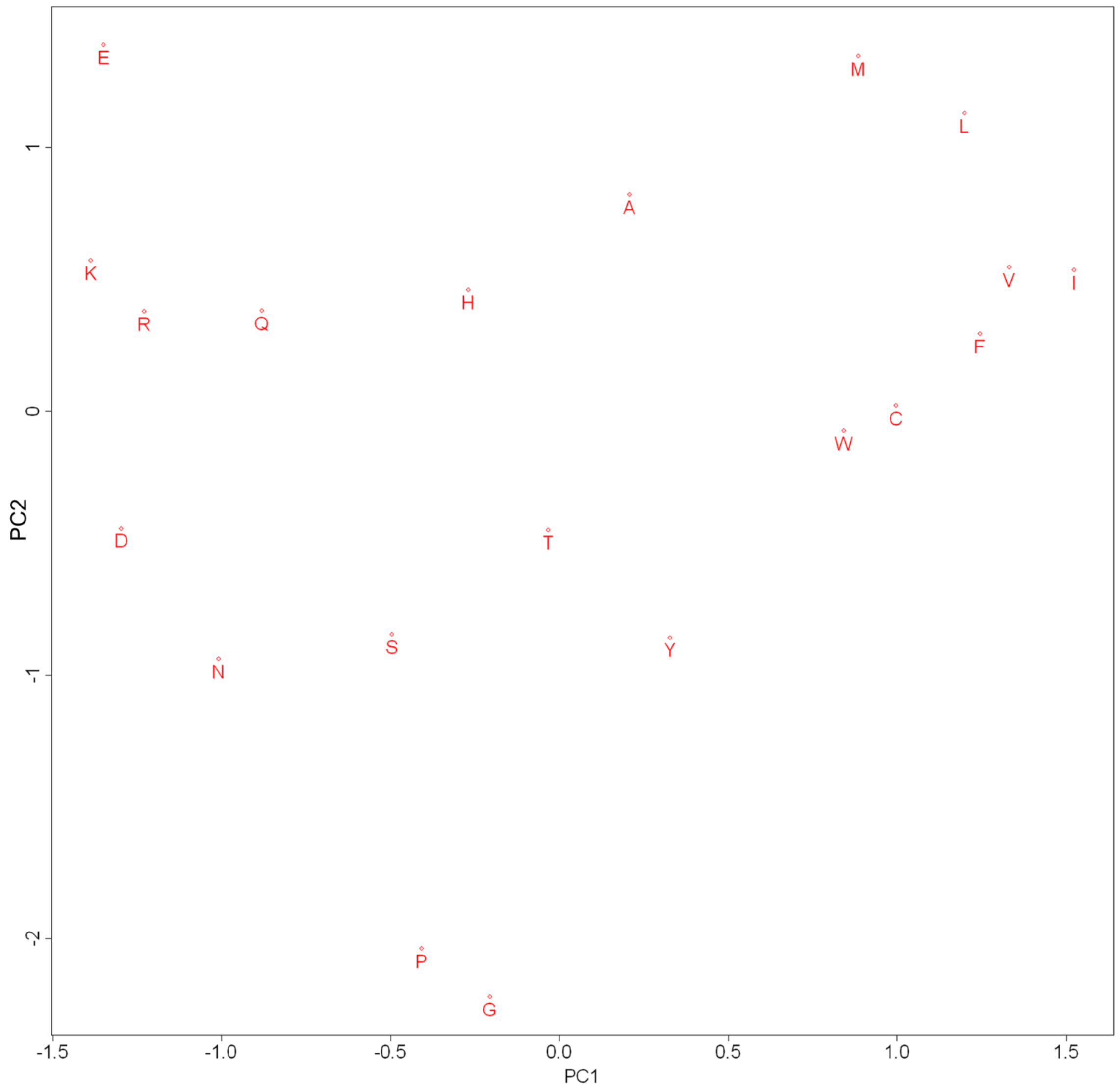

Supporting figure S19. Plot of the first two PCs in the FASGAI descriptor set.

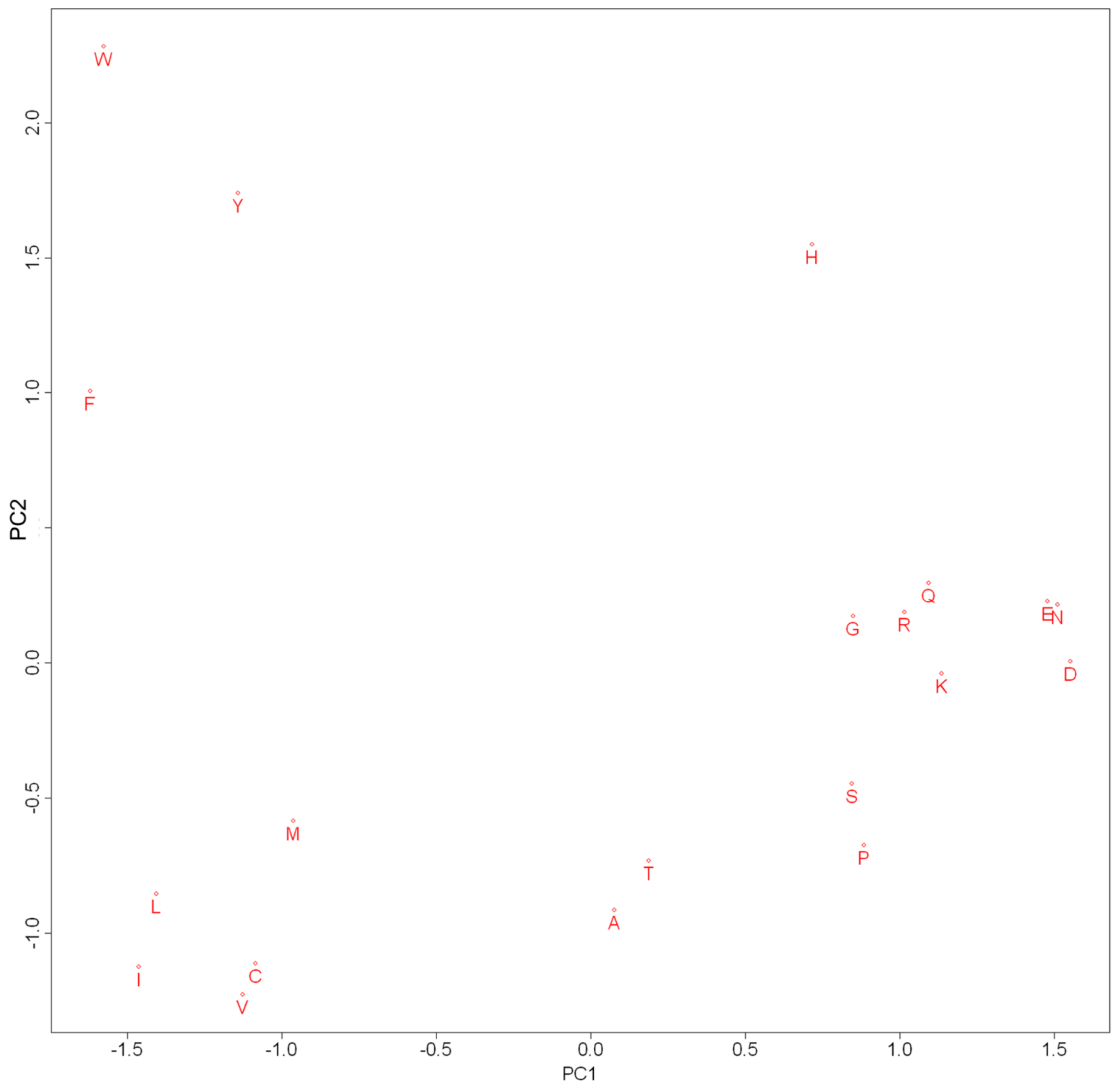

Supporting figure S20. Plot of the first two PCs in the BLOSUM descriptor set.
